# Synthesis and antifungal properties of papulacandin derivatives

**DOI:** 10.3762/bjoc.8.82

**Published:** 2012-05-14

**Authors:** Marjolein van der Kaaden, Eefjan Breukink, Roland J Pieters

**Affiliations:** 1 Department of Medicinal Chemistry and Chemical Biology. Utrecht Institute for Pharmaceutical Sciences, Utrecht University, P.O. Box 80082, 3508 TB Utrecht, The Netherlands; 2 Department of Biochemistry of Membranes, Bijvoet Centre for Biomolecular Research, Utrecht University, Padualaan 8, Utrecht, The Netherlands

**Keywords:** antifungal agents, carbohydrate, papulacandins, spiroketal

## Abstract

Derivatives of an antifungal agent that targets the β-(1,3)-D-glucan synthase, papulacandin D, were synthesized and tested for activity. The papulacandin D structure contains a challenging benzannulated spiroketal unit, which is introduced in a palladium-catalyzed cross-coupling reaction of a glycal silanolate and an aryl iodide followed by an oxidative spiroketalization. Four different variants were made, differing in the nature of the acyl side chain with respect to the length, and in the number and stereochemistry of the double bonds. Moderate biological activity was observed for the derivatives with a side chain based on palmitic acid and linoleic acid.

## Introduction

**Figure 1:**
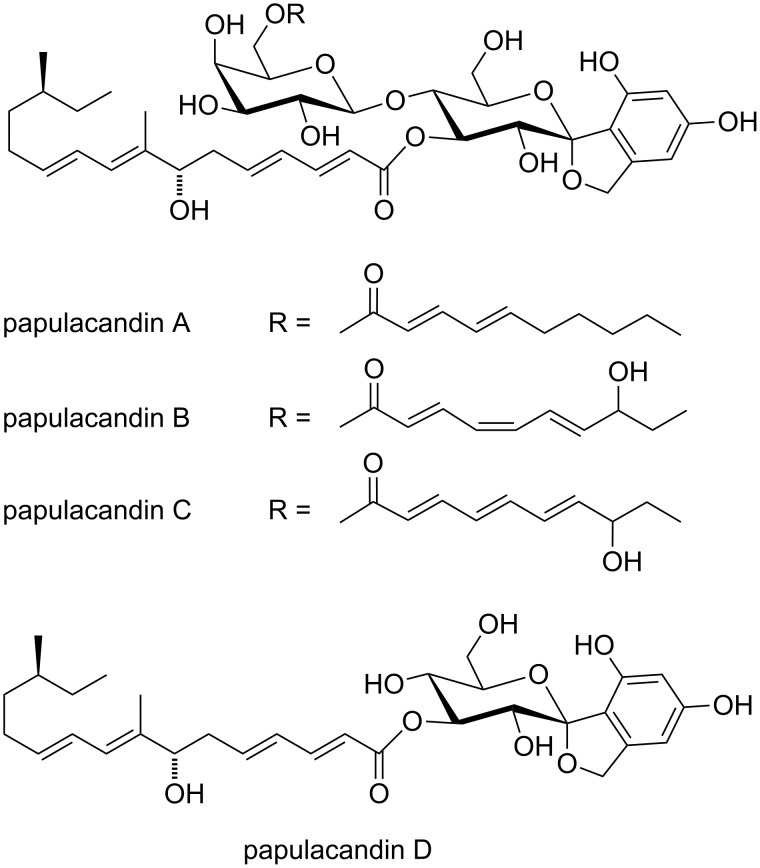
The papulacandins.

In recent years, a steady increase in the incidence of opportunistic fungal infections in immunocompromised patients has been observed [1]. Treatment failure is frequent and mortality is unacceptably high in high-risk patients [2]. Reasons for this include delayed diagnosis, toxicity and low bioavailability of current drugs, and the development of antifungal drug resistance [1]. Clearly, there is a need for new antifungal therapeutics. The papulacandins are a series of naturally occurring antifungal agents whose isolation and characterization were initially reported by Traxler and co-workers in 1977 (Figure 1) [3–4]. They contain a benzannulated spiroketal unit, which has been the signature of a wide series of bioactive natural products [5] and has inspired ample synthetic activity [5–8]. The papulacandins A–E were isolated from the fermentation broths of *Papularia sphaerosperma* [3]. They block the synthesis of β-(1,3)-D-glucan by inhibition of β-(1,3)-D-glucan synthase [9–12]. The β-(1,3)-D-glucan is an integral and essential component of the fungal cell wall [13] and the dominant glucan in the cell wall of most medically important fungi, and therefore it is a target that is being actively pursued [14]. The papulacandins have demonstrated a very high specific activity against several yeasts, but they are largely inactive against filamentous fungi, bacteria and protozoa [3]. Recently, in a direct comparison, papulacandin B was shown to be superior in some aspects compared to the drug caspofungin and a few drug candidates [15]. Its interesting biological activity has stimulated the search for new members of the papulacandin family, and several new compounds that are structurally related have been isolated, such as chaetiacandein [16–17], L-687,781 [18–19], Mer-WF3010 [20–21], BU-4794F [22], fusacandins [23–24], saricandin and PF-1042 [25], corynecandin [26–27] and the F-10748 series [28]. These structures vary mostly with respect to the two partially unsaturated acyl chains on the sugars. Small changes in these fatty acid tails have only small effects on the activity. However, more drastic changes in these tails or the lack of one of the tails drastically reduce activity, in comparison with the most active member of this family: papulacandin B. For the papulacandins it was shown that the substituted galactose is not essential for activity, since papulacandin D is still active, but it does increase the potency. Apparently, the situation is different for the related disaccharide saricandan carrying different acyl chains, for which the monosaccharide analogue was not active [29]. Removing the galactose acyl chain from papulacandin B results in material that can still inhibit the target (in a spheroplasts glucan synthesis assay) but cannot reach the target site of *C. albicans* [12]. The same is true for the hydrogenated form of papulacandin D [12]. Clearly some form of unsaturation is needed to maintain biological activity, but it is not known to what extent unsaturation (and alkylation and/or hydroxylation) needs to be present for the compound to reach the target site.

The papulacandins represent a synthetic challenge and as such the class has stimulated numerous synthetic studies. So far, only the total synthesis of papulacandin D, the simplest member of the family, has been published. This was reported by two different research groups [30–33] and additionally a number of different synthetic routes [34–38] to the spiroketal core structure have been published, including a hetero Diels–Alder reaction [39], a palladium(0)-catalyzed coupling [40–45] and a condensation reaction [46–47]. We herein report our efforts on the synthesis of papulacandin derivatives by total synthesis and the evaluation of their antifungal properties. The synthetic approach is largely based on the Denmark route [31] with a few notable deviations.

## Results

**Scheme 1:**
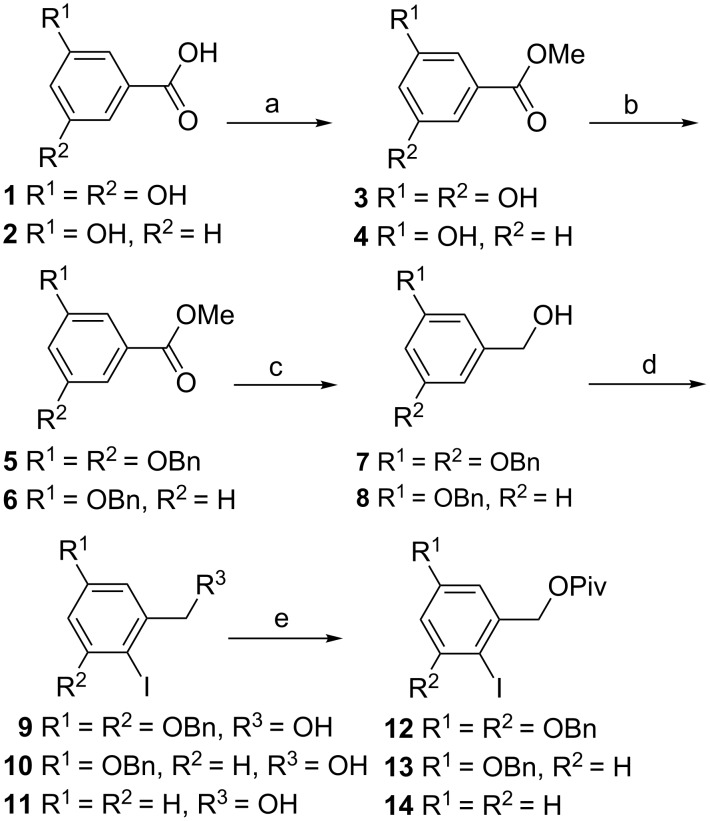
(a) H_2_SO_4_, MeOH, reflux, 18 h, **3**: 98%; (b) BnBr, K_2_CO_3_, acetone, reflux, 18 h; (c) LiAlH_4_, THF, rt, 10–30 min, **7**: 92% over three steps, **8**: 65% over three steps; (d) NIS, CHCl_3_, rt, 18 h, **9** and **11** quant. or I_2_, CF_3_COOAg, CHCl_3_, rt, 1 h, **10**: 96%; (e) pivaloyl chloride, pyridine, CH_2_Cl_2_, rt, 3–18 h, **12**: 95% over 2 steps, **13**: 96%, **14**: 98%.

The synthesis started with the anomeric aromatic group, i.e., the eventual spiroketal group of the glucose, to be introduced as an aryliodide with an ortho hydroxymethylene as in **12**. The commercially available 3,5-dihydroxybenzoic acid (**1**) was esterified, followed by benzylation of the aromatic hydroxy groups and reduction of the ester to the primary alcohol to give **7** (Scheme 1). This compound was iodinated by using *N*-iodosuccinimide (NIS), and finally the free hydroxy group was protected with a pivaloyl group to give aryl iodide **12**. Derivatives **13** and **14**, providing alternative core structures, were prepared similarly.

**Scheme 2:**
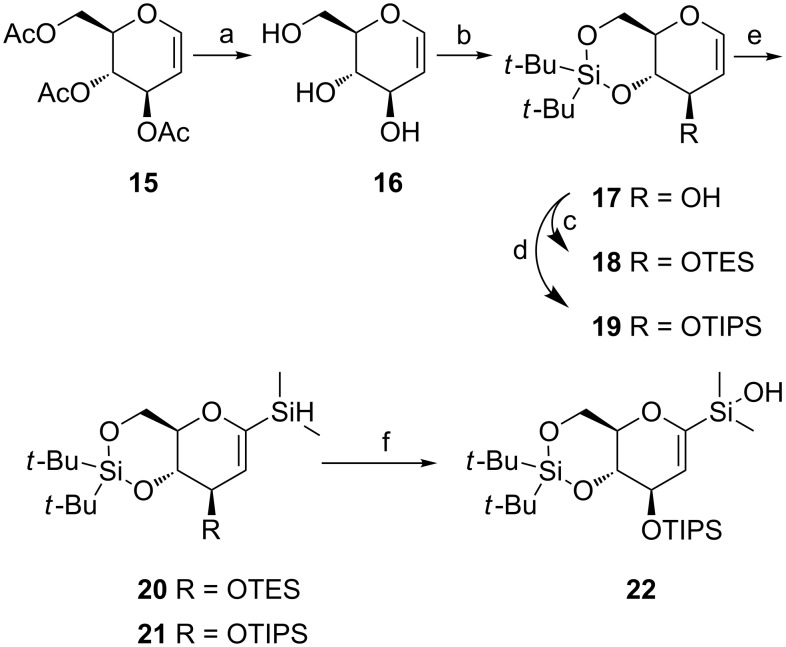
(a) NaOMe in MeOH, MeOH, rt, 1 h, 96%; (b) (*t*-Bu)_2_Si(OTf)_2_, pyridine, DMF, −40 °C to rt, 2 h, 90%; (c) TESCl, pyridine, CH_2_Cl_2_, rt, 18 h, 93%; (d) TIPSCl, imidazole, DMF, 60 °C, 2 d, 74%; (e) *t*-BuLi, Me_2_SiHCl, Et_2_O, −78 °C to rt, 3 h, **20**: no yield, **21**: 99%; (f) [RuCl_2_(*p*-cymene)]_2_, H_2_O, benzene/CH_3_CN, rt, 4 h, 95%.

The glucose moiety at the core of the structure, as present in papulacandin D, was approached from glycal **15**. The aim was to obtain a silanol such as **22** for coupling to an aryl iodide in the palladium-catalyzed cross-coupling reactions of silanolates, as pioneered and applied by the Denmark group (Scheme 2) [48]. The synthesis started with the saponification of **15**, followed by the protection of O-4 and O-6 by using di-*tert*-butylsilyl bis(trifluoromethanesulfonate). Subsequent protection of O-3 with TES-Cl, gave the fully protected glucal **18**. According to Denmark et al. [31], lithiation and silylation of **18** should give **20**, but in our hands a more complicated mixture resulted. We concluded that under our conditions deprotonation of the protecting group (protons α to silicon) may be competitive with the desired α-lithiation next to oxygen. Use of the more substituted TIPS silyl protecting group in **19** indeed solved this problem and yielded **21** in almost quantitative yield. Additional test reactions confirmed this trend as the corresponding 3,4,6-tri-*O*-(triisopropylsilyl)-D-glucal (**52**) yielded a single product after lithiation (Supporting Information File 1) and the 3,4,6-tri-*O*-(*tert*-butyldimethylsilyl)-D-glucal gave a mixture of products. Finally, compound **21** was oxidized to give silanol building block **22**.

**Scheme 3:**
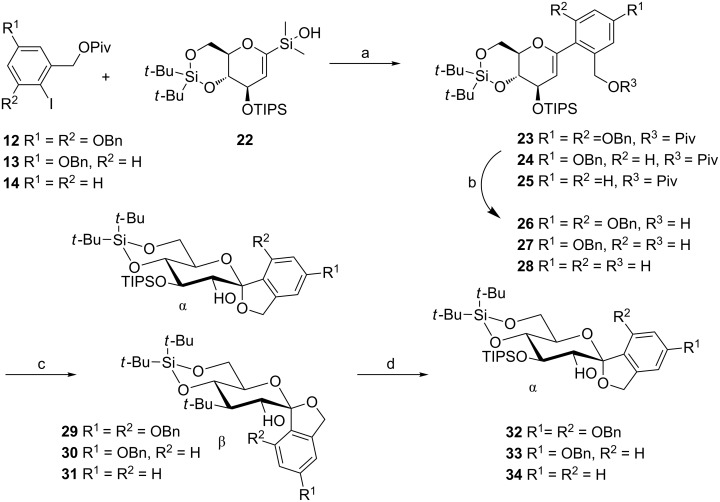
(a) Pd_2_(dba)_3_∙CHCl_3_, NaO*t*-Bu, toluene, 50 °C, 6–20 h, **23**: 72%, **24**: 79%, **25**: 62%; (b) DiBAL-H, CH_2_Cl_2_, −78 °C to rt, 0.5–1 h, **26**: 86%, **27**: 70%, **28**: 75%; (c) *m*-CPBA, NaHCO_3_, CH_2_Cl_2_, 0 °C to rt, 2–2.5 h; (d) HCl, CHCl_3_, rt, 1 h, **32**: 91% over two steps, **33**: 83% over two steps, **34**: 86% over two steps.

With building blocks **13** and **22** in hand, the palladium-catalyzed cross-coupling reaction was performed by using Pd_2_(dba)_3_ to give **23** (Scheme 3). Then the pivaloyl ester was selectively cleaved by using DIBAL-H, followed by oxidative spiroketalization under basic conditions to give the α- and β-anomers **29**, which were separable using column chromatography. However this was not necessary: The β-anomer could readily be converted into the more stable α-anomer **32** by using a solution of 0.1 M HCl in chloroform. Similarly, the related iodides **14** and **15** performed well in this sequence yielding **33** and **34**.

**Scheme 4:**
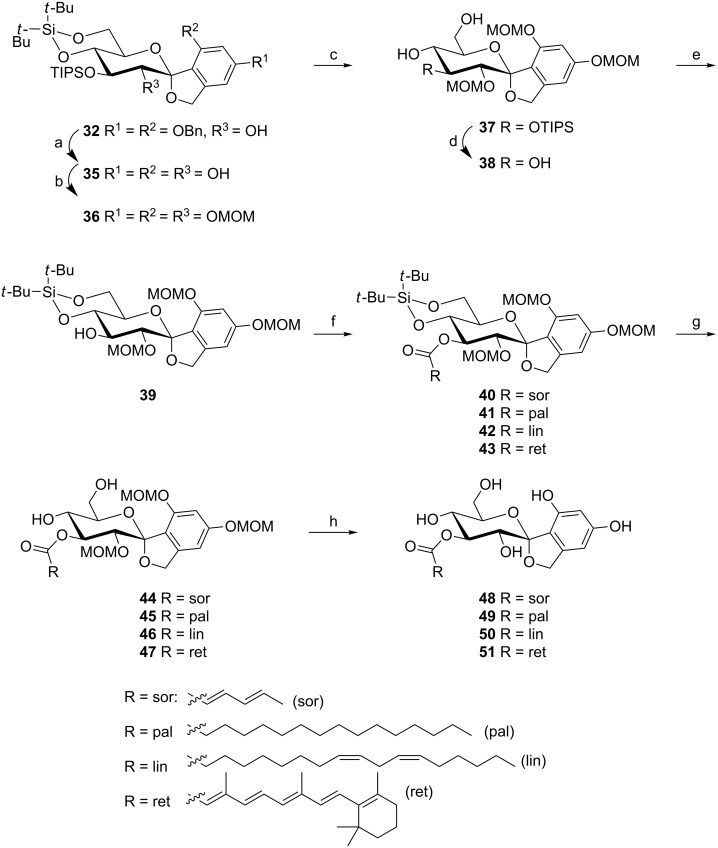
(a) Pd/C, NaHCO_3_, H_2_, THF, rt, 1.5 h, 98%; (b) MOMCl, DiPEA, DMAP, CH_2_Cl_2_, rt, 4 d, 78%; (c) TBAHF, THF, rt, 2 d, 84%; (d) TBAF∙3H_2_O, THF, rt, 3 d; (e) *t-*Bu_2_Si(OTf)_2_, pyridine, DMF, −40°C to rt, 2 h, 84% over two steps; (f) RCOOH, NEt_3_, 2,4,6-trichlorobenzoyl chloride, toluene, rt, 1 h, **39**, DMAP, toluene, rt, 3 h, **40**: 89%, **41**: quant., **42**: 92%, **43**: 90%; (g) TBAHF, THF, rt, 2–3 d, **44**: 25%, **45**: 85%, **46**: 86%, **47**: 39%; (h) Dowex 50 × 8, MeOH, 50 °C, 20 h **48**: 13%, **50**: 43%, **51**: 7% or Sc(OTf)_3_, 1,3-propanediol, CH_3_CN, 50 °C, 3 h, **49**: 9%, isolated yields after preparative HPLC and lyophilization.

The first step in the synthesis of the mimics of papulacandin D was debenzylation of **32**, since these groups cannot be removed in the presence of olefines in the final product side chain (Scheme 4). The liberated hydroxy groups were reprotected with MOM groups by using MOM-Cl to give **36**. Then the glucose O(3) needed to be selectively deprotected for the incorporation of the fatty acid tail. Unfortunately, this was not possible from **36** because of the use of the more stable TIPS group in this synthesis. Therefore, the cyclic silyl protecting group was first removed by using TBAHF in THF, followed by the deprotection of the TIPS group with TBAF and reintroduction of the cyclic silyl protecting group, giving **39**. To circumvent this problem a silanol building block analogue of **22** bearing three TIPS protecting groups was also used (Supporting Information File 1).

At this stage, four commercially available side-chain acids, comprising variations in their degree of unsaturation, length and branching, were coupled: sorbic acid (sor), palmitic acid (pal), linoleic acid (lin) and *trans*-retinoic (ret) acid. Their coupling by 2,4,6-trichlorobenzoyl chloride to **39** gave the desired compounds **40**–**43** in good yields. Following this the cyclic disilyl protecting group was removed by TBAHF yielding **44**–**47**. Finally, removal of the MOM group proved to be a challenge. The use of Dowex (50 × 8) acidic resin in dry MeOH gave the most consistent results, but also the use of Sc(OTf)_3_ in dry acetonitrile in the presence of 1,3-propanediol [49] yielded the deprotected product. The final compounds were purified by preparative HPLC. Due to some degradation during deprotection, the final yields were low. For **51** no clear NMR spectra could be obtained, possibly caused by aggregation. This was not a problem for the other three compounds.

The final products were tested for inhibition of *Candida albicans*. No inhibition was observed for the all-*trans-*retinoic acid derivative **51** and the sorbic acid derivative **48**. The MIC for the linoleic acid derivative **50** was measured as 100 μg/mL. The saturated palmitic acid derivative **49** was slightly more potent with an MIC of 88 μg/mL.

## Discussion

Starting from glycal the synthesis of papulacandin D derivatives was undertaken with an initial focus on the acyl side chain, a clearly sensitive area for activity. The synthesis followed the general approach of Denmark et al. [31] with the palladium-catalyzed cross-coupling reactions of silanolates as its main feature. In our hands the reaction between glycal **18** and *t*-BuLi gave a mixture of products, possibly due to competing deprotonation of the hydrogens α to the Si in the TES group. Moving to the more substituted TIPS group at this position gave a clean reaction and allowed us to proceed, albeit with a more complicated endgame. It should be pointed out that the unplanned synthetic route eventually taken here due to the troublesome lithiation of glycal **18**, led us to compound **37**. This compound opens the way to a total synthesis of disaccharides such as the papulacandins A–C. Selective protection of the hydroxy group of C(6) followed by glycosylation of the hydroxy group at C(4), should accomplish this.

In our biological assay we did not see any activity for the sorbic acid derivative **48**, suggesting that this tail may be too short to be effective. The same was true for the retinoic acid derivative **51**, but its identity was also uncertain due to instability. Some activity was observed for linoleic acid derivative **50**, which contains two *cis* double bonds in contrast to the four *trans* double bonds of papulacandin D. The highest activity was observed for the saturated palmitic acid derivative **49**, whose tail was of the same length as the one from papulacandin D. The fact that we observed some activity with **49** is encouraging, since this was not the case for the hydrogenated form of papulacandin D [12], although that compound was able to block the target in vitro. This could be the case for the compounds described here as well, but this was not investigated. In summary, we synthesized a number of papulacandin D derivatives with differing acyl side chains. Even though the compounds were moderately active at best it is clear that the side chains are of crucial importance. The synthetic route taken will allow future variation of the aromatic core and will extend the synthesis to the more potent disaccharide papulacandins.

## Supporting Information

File 1 Synthetic procedures, the biological assay procedure and spectral data.
