# Supplementary material for: Synthesis and antifungal properties of papulacandin derivatives
Source: Beilstein J Org Chem. 2012 May 14;8:732–7. doi: 10.3762/bjoc.8.82 (PMC3388860; doi:10.3762/bjoc.8.82)

Supporting Information

for

Synthesis and antifungal properties of papulacandin derivatives

Marjolein van der Kaaden[1], Eefjan Breukink[2] and Roland J. Pieters*[1]

Address: [1]Department of Medicinal Chemistry and Chemical Biology. Utrecht Institute for Pharmaceutical Sciences, Utrecht University, P.O. Box 80082, 3508 TB Utrecht, The Netherlands and [2]Department Biochemistry of Membranes, Bijvoet Centre for Biomolecular Research, Utrecht University, Padualaan 8, Utrecht, The Netherlands

Email: Roland J. Pieters* - R.J.Pieters@uu.nl

* Corresponding author

Synthetic procedures, the biological assay procedure and spectral data

General

All chemicals were obtained from commercial sources and used without further purification, unless stated otherwise. THF and Et_2O were freshly distilled from $LiAlH_4$. The reactions were monitored by thin-layer chromatography (TLC) on Merck pre-coated silica gel 60 F_{254} (0.25 mm) plates. Spots were visualized by UV light, H_2SO_4 and/or $K_2CO_3/KMnO_4$. Column chromatography was carried out by using Silicycle Ultrapure silicagel (40–63 μm). ^{1}H NMR spectra were recorded on a Varian G-300 spectrometer or a Varian Unity INOVA-500 spectrometer and chemical shifts (δ) are given in ppm relative to TMS (0.00 ppm). For measurements in CD_3OD, the residual solvent peak (3.31 ppm) was used as a reference. ^{13}C NMR spectra were recorded, in most cases by using the attached proton test (APT) pulse sequence, on a Varian G-300 spectrometer and chemical shifts (δ) are given in ppm relative to $CDCl_3$ (77.0 ppm). For measurements in CD_3OD, the residual solvent peak (49.0 ppm) was used as a reference. HSQC and TOCSY NMR spectra were recorded at 300 K with a Varian Unity INOVA-500 spectrometer. Electrospray ionization mass spectrometry (ESI MS) was performed on a Shimadzu LCMS QP8000 system in positive ionization mode. Analytical HPLC runs were performed on a Shimadzu automated HPLC system with a reversed-phase column that was equipped with an evaporative light-scattering detector (PL-ELS 1000, Polymer Laboratories, Amherst, MA, USA) and a UV–vis detector operating at 220 and 254 nm. Preparative HPLC runs were performed on an Applied Biosystems workstation. Elution was effected by using a linear gradient of 5% CH_3CN/0.1% TFA in H_2O to 5% H_2O/0.1% TFA in CH_3CN.

Methyl 3,5-dihydroxybenzoate (3)

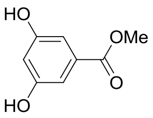

3,5-Dihydroxybenzoic acid (**1**, 7.71 g, 50 mmol) was dissolved in dry MeOH (270 mL), and a catalytic amount of sulfuric acid (500 μL) was added. This reaction mixture was stirred at reflux temperature overnight. After neutralization with 2 N NaOH (aq), the resulting mixture was concentrated and then dissolved in EtOAc and 1 N $KHSO_4$ (aq). The layers were separated and the organic layer was washed once with brine, dried over Na_2SO_4, filtered and concentrated to give the product in 98% yield (8.26 g, 49.12 mmol). ^1H NMR (300 MHz, CD_3OD) δ 3.84 (s, 3H, CH_3), 4.86 (br s, 2H, 2 × OH), 6.48 (t, 1H, J = 2.4 Hz, C_4H), 6.92 (d, 2H, J = 2.4 Hz, C_2H, C_6H); ^{13}C NMR (75 MHz, CD_3OD) δ 52.5 (CH_3), 108.2, 108.6, 108.8 (C_2H, C_4H, C_6H), 133.0 (C_1H), 159.7 (C_3, C_5), 168.7 (C=O).

Methyl 3-hydroxybenzoate (4)

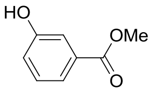

3-Hydroxybenzoic acid (**2**, 6.91 g, 50 mmol) was dissolved in dry MeOH (300 mL), and a catalytic amount of sulfuric acid (500 μL) was added. This reaction mixture was stirred at reflux temperature overnight. After neutralization with 2 N aq NaOH, the resulting mixture was concentrated and then dissolved in EtOAc. The layers were separated and the organic layer was washed once with H_2O, once with brine, dried over Na_2SO_4, filtered and concentrated to give the product in 97% yield (7.35 g, 48.31 mmol). This crude product was directly used in the next reaction.

Methyl 3,5-dibenzyloxybenzoate (5)

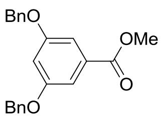

Methyl 3,5-dihydroxybenzoate (**3**, 4.20 g, 25 mmol) was dissolved in acetone (35 mL), and K_2CO_3 (8.64 g, 62.5 mmol) and benzyl bromide (7.44 mL, 62.5 mmol) were added. The resulting suspension was stirred at reflux

temperature overnight. Then the mixture was filtered and the solid material (KBr, which was formed during the reaction) was rinsed with dry Et_2O. The filtrate was concentrated to give crude product **5**, which was directly used in the next reaction.

Methyl 3-benzyloxybenzoate (6)

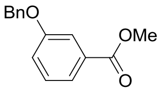

Methyl 3-hydroxybenzoate (**4**, 50 mmol) was dissolved in acetone (70 mL), and K_2CO_3 (8.64 g, 62.5 mmol) and benzyl bromide (7.44 mL, 62.5 mmol) were added. The resulting suspension was stirred at reflux temperature overnight. Then the mixture was filtered and the solid material (KBr, which was formed during the reaction) was rinsed with dry Et_2O. The filtrate was concentrated to give crude product **6**, which was directly used in the next reaction.

3,5-Dibenzyloxybenzyl alcohol (7)

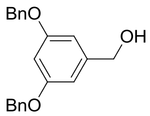

Under an argon atmosphere, $LiAlH_4$ (3.04 g, 80 mmol) was suspended in freshly distilled THF (200 mL). Crude methyl 3,5-dibenzyloxybenzoate (**5**, 25 mmol) was also dissolved in freshly distilled THF (50 mL) and this was added dropwise to the suspension. This reaction mixture was stirred at rt for 30 min and then cooled down to 0 °C. After careful quenching with H_2O (30 mL) and 4 N aqueous NaOH (30 mL), Et_2O was added. The mixture was filtered through hyflo and the hyflo pad was washed with Et_2O. The layers were separated and the organic layer was washed once with brine, dried over Na_2SO_4, filtered and concentrated. Column chromatography using 4/1 to 3/1 hexanes/EtOAC gave product **7** in 92% yield over two steps (7.35 g, 22.94 mmol). ^1H NMR (300 MHz, $CDCl_3$) δ 4.51 (s, 2H, CH_2OH), 4.95 (s, 4H, 2 × OCH_2Ph), 6.51 (t, 1H, J = 2.1 Hz, C_4H), 6.56 (d, 2H, J = 2.1 Hz, C_2H, C_6H), 7.31 (m, 10H, 2 × C_6H_5); ^{13}C NMR (75 MHz, $CDCl_3$) δ 64.9

(CH_2OH), 69.9 (2 × OCH_2Ph), 101.1 (C_4H), 105.6 (C_2H, C_6H), 127.4, 127.9, 128.5 (CH of Ph), 136.7 (C of Ph), 143.4 (C_1), 160.0 ($C_{3,}$ C_5).

3-Benzyloxybenzyl alcohol (8)

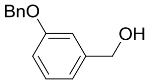

Under an argon atmosphere, $LiAlH_4$ (6.07 g, 160 mmol) was suspended in freshly distilled THF (200 mL). Crude methyl 3-benzyloxybenzoate (**6**, 50 mmol) was also dissolved in freshly distilled THF (100 mL) and this solution was added dropwise to the suspension. This reaction mixture was stirred at rt for 10 min and then cooled down to 0 °C. After careful quenching with H_2O, Et_2O was added. This mixture was filtered through hyflo and the hyflo pad was rinsed with Et_2O. The layers were separated and the organic layer was dried over Na_2SO_4, and filtered and concentrated to give product **8** in 65% yield over three steps (6.97 g, 32.53 mmol). ^1H NMR (300 MHz, $CDCl_3$) δ 4.65 (d, 2H, J = 5.4 Hz, CH_2OH), 5.06 (s, 2H, OCH_2Ph), 6.91 (m, 2H, C_4H, C_6H), 7.00 (s, 1H, C_2H), 7.34 (m, 6H, C_5H, C_6H_5); ^{13}C NMR (75 MHz, $CDCl_3$) δ 65.2 (CH_2OH), 69.9 (OCH_2Ph), 113.2, 114.1 (C_2H, C_4H), 119.3 (C_6H), 127.4, 127.9, 128.5 (CH of Ph), 129.6 (C_5H), 136.9 (C of Ph), 142.6 (C_1), 159.0 ($C_{3,}$).

3,5-Dibenzyloxy-2-iodobenzyl alcohol (9)

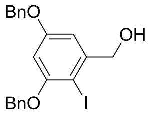

3,5-Dibenzyloxybenzyl alcohol (**7**, 7.35 g, 22.94 mmol) was dissolved in dry $CHCl_3$ (50 mL), and *N*-iodosuccinimide (7.74 g, 34.41 mmol) was added. The round-bottom flask was wrapped in aluminium foil and then the reaction mixture was stirred at rt overnight. Then it was diluted with EtOAc (75 mL) and filtered through hyflo, and the hyflo pad was washed with EtOAc. H_2O was added to the filtrate, and the layers were separated. The aqueous layer was extracted once

with EtOAc. The combined organic layers were washed once with saturated aqueous $Na_2S_2O_5$, dried over Na_2SO_4, filtered and concentrated to give crude product **9**, which was directly used in the next reaction. ^{1}H NMR (300 MHz, $CDCl_3$) δ 4.63 (s, 2H, CH_2OH), 5.00 (s, 2H, OCH_2Ph), 5.06 (s, 2H, OCH_2Ph), 6.46 (d, 1H, *J* = 2.7 Hz, C_4H), 6.80 (d, 1H, *J* = 2.7 Hz, C_6H), 7.39 (m, 10H, 2 × C_6H_5); ^{13}C NMR (75 MHz, $CDCl_3$) δ 69.5, 70.2, 70.9 (CH_2OH, 2 × OCH_2Ph), 78.8 (C_2I), 100.3 (C_4H), 106.5 (C_6H), 126.9, 127.5, 127.8, 128.1, 128.5, 128.6 (CH of Ph), 136.3, 136.4 (2 × C of Ph), 144.8 (C_1), 157.6 (C_5), 160.3 (C_3).

5-Benzyloxy-2-iodobenzyl alcohol (10)

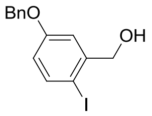

Iodine was dissolved in dry $CHCl_3$ (20 mL). 3-Benzyloxybenzyl alcohol (**8**, 429 mg, 2.0 mmol) and trifluoroacetic acid silver salt were suspended in $CHCl_3$ (7 mL). The iodine solution was then added to the suspension and this reaction mixture was stirred at rt for 1 h, after which it was filtered off over hyflo. The hyflo pad was rinsed with CH_2Cl_2. The filtrate was washed with saturated aqueous $Na_2S_2O_3$, dried over Na_2SO_4, filtered and concentrated. Column chromatography using hexanes, 4/1 hexanes/EtOAc gave product **10** in 96% yield (650 mg, 1.91 mmol). ^{1}H NMR (300 MHz, $CDCl_3$) δ 4.59 (d, 2H, *J* = 4.8 Hz, CH_2OH), 5.05 (s, 2H, OCH_2Ph), 6.65 (dd, 1H, *J* = 3.0, 8.7 Hz, C_4H), 7.13 (d, 1H, *J* = 3.0 Hz, C_6H), 7.37 (m, 5H, C_6H_5), 7.63 (d, 1H, *J* = 8.4 Hz, C_3H); ^{13}C NMR (75 MHz, $CDCl_3$) δ 69.1 (CH_2OH), 70.1 (OCH_2Ph), 85.6 (C_2I), 115.2, 116.1 (C_4H, C_6H), 127.4, 128.1, 128.6 (CH of Ph), 136.5 (C of Ph), 139.6 (C_3H), 143.7 (C_1), 159.0 ($C_{5,}$).

3,5-Dibenzyloxy-2-iodobenzyl pivalate (12)

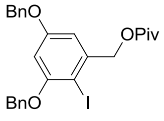

Crude 3,5-dibenzyloxy-2-iodobenzyl alcohol (**9**, 22.94 mmol) was

dissolved in dry CH_2Cl_2 (90 mL), and pyridine (2.78 mL, 34.41 mmol) and pivaloyl chloride (7.06 mL, 57.35 mmol) were added. This reaction mixture was stirred at rt for 3 h and then diluted with H_2O and CH_2Cl_2. The layers were separated and the organic layer was washed once with saturated aqueous $NaHCO_3$, dried over Na_2SO_4, filtered and concentrated. Three × column chromatography using 15/1 hexanes/EtOAC gave product **13** in 95% yield over two steps (11.54 g, 21.76 mmol). ^{1}H NMR (300 MHz, $CDCl_3$) δ 1.25 (s, 9H, Piv), 5.02 (s, 2H, CH_2OPiv), 5.09 (s, 2H, OCH_2Ph), 5.12 (s, 2H, OCH_2Ph), 6.50 (d, 1H, J = 2.7 Hz, C_4H), 6.68 (d, 1H, J = 2.7 Hz, C_6H), 7.37 (m, 10H, 2xC_6H_5); ^{13}C NMR (75 MHz, $CDCl_3$) δ 27.3 (C(CH$_3$)$_3$), 38.9 (C(CH_3)$_3$), 70.2, 71.0 (CH_2OPiv, 2 × OCH_2Ph), 79.8 (C_2I), 100.5 (C_4H), 107.2 (C_6H), 126.9, 127.4, 127.8, 128.1, 128.5, 128.6 (CH from Ph), 136.2, 136.3 (2 × C from Ph), 140.7 (C_1), 157.9 (C_5), 160.1 (C_3), 177.8 (C=O).

5-Benzyloxy-2-iodobenzyl pivalate (13)

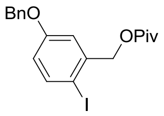

5-Benzyloxy-2-iodobenzyl alcohol (**10**, 5.43 g, 15.96 mmol) was dissolved in dry CH_2Cl_2 (70 mL), and pyridine (1.9 mL, 23.94 mmol) and pivaloyl chloride (4.9 mL, 39.90 mmol) were added. This reaction mixture was stirred at rt for 18 h and then diluted with H_2O. The layers were separated, the aqueous layer was extracted once with CH_2Cl_2 and the combined organic layers were washed once with saturated aqueous $NaHCO_3$ and once with brine, dried over Na_2SO_4, filtered and concentrated. Column chromatography using hexanes, 20/1 hexanes/EtOAC gave product **14** in 96% yield (6.52 g, 15.37 mmol). ^{1}H NMR (300 MHz, $CDCl_3$) δ 1.25 (s, 9H, Piv), 5.04 (s, 4H, CH_2OPiv, OCH_2Ph), 6.68 (dd, 1H, J = 3.0, 8.7 Hz, C_4H), 7.01 (d, 1H, J = 2.7 Hz, C_6H), 7.36 (m, 5H, C_6H_5), 7.68 (d, 1H, J = 8.7 Hz, C_3H); ^{13}C NMR (75 MHz, $CDCl_3$) δ 27.3 (C(CH$_3$)$_3$), 38.9 (C(CH_3)$_3$), 69.8

(CH_2OPiv), 70.1 (OCH_2Ph), 86.4 (C_2I), 116.0, 116.4 (C_4H, C_6H), 127.4, 128.1, 128.6 (CH of Ph), 136.4 (C of Ph), 139.7 (C_1), 139.9 (C_3H), 159.1 ($C_{5,}$), 177.9 (C=O).

2-Iodobenzyl pivalate (14)

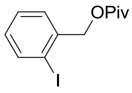

2-Iodobenzyl alcohol (**11**, 2.34 g, 10 mmol) was dissolved in dry CH_2Cl_2 (40 mL), pyridine (1.2 mL, 15 mmol) was added and pivaloyl chloride (3.1 mL, 25 mmol) was added dropwise. This reaction mixture was stirred at rt for 18 h, after which it was diluted with H_2O (70 mL). The aqueous layer was extracted once with CH_2Cl_2 and the combined organic layers were washed once with saturated aqueous $NaHCO_3$ and once with brine, dried over Na_2SO_4, filtered and concentrated. Column chromatography using hexanes, 20/1 hexanes/EtOAc gave product **15** in 98% yield (3.13 g, 9.84 mmol). ^{1}H NMR (300 MHz, $CDCl_3$) δ 1.26 (s, 9H, Piv), 5.10 (s, 2H, CH_2OPiv), 7.02 (m, 1H, C_4H), 7.35 (m, 2H, C_5H, C_6H), 7.84 (d, 1H, J = 7.8 Hz, C_3H); ^{13}C NMR (75 MHz, $CDCl_3$) δ 27.3 ($C(CH_3)_3$), 38.9 ($C(CH_3)_3$), 69.9 (CH_2OPiv), 98.1 (C_2I), 128.3, 129.2, 129.7 (C_4H, C_5H, C_6H), 138.7 (C_1), 139.5 (C_3), 177.8 (C=O).

3,4,6-Tri-*O*-(triisopropylsilyl)-D-glucal (52)

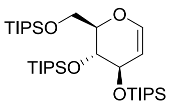

Compound **16** (961 mg, 6.58 mmol) was dissolved in dry DMF (50 mL), and imidazole (4.48 g, 65.8 mmol), triisopropylsilyl chloride (7.0 mL, 32.9 mmol) and a catalytic amount of DMAP were added. This reaction mixture was heated to 60 °C and stirred at this temperature for 44 h, afterwards the reaction mixture was cooled down to rt and stirred for 24 h at rt. The resulting reaction mixture was diluted with EtOAc and washed twice with H_2O, once with brine, dried over Na_2SO_4, filtered and concentrated. Column chromatography using 4/1 hexanes/CH_2Cl_2 gave product **52** in 60% yield (2.43 g, 3.95 mmol). ^{1}H NMR

(300 MHz, $CDCl_3$) δ 1.06 (m, 63H, 3 × TIPS), 3.82 (dd, 1H, *J* = 11.1, 3.6 Hz, C_6H), 3.95 (m, 1H, C_3H), 4.07 (m 2H, C_4H, C_6H), 4.23 (m, 1H, C_5H), 4.80 (m, 1H, C_2H), 6.35 (d, 1H, *J* = 6.3 Hz, C_1H); ^{13}C NMR (75 MHz, $CDCl_3$) δ 12.0, 12.3, 12.5 (Si*C*H(CH_3)$_2$), 18.0, 18.1 (SiCH(*C*H_3)$_2$), 62.1 (C_6H_2), 65.0, 70.3, 80.7 (C_3H, C_4H, C_5H), 100.3 (C_2H), 142.7 (C_1H).

1-*C*-Dimethylsilyl-3,4,6-tri-*O*-(triisopropylsilyl)-D-glucal (53)

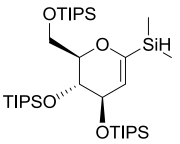

Compound **52** (1.57 g, 2.55 mmol) was dissolved in freshly distilled Et_2O (30 mL) and placed under a N_2 atmosphere. The solution was cooled down to −78 °C and then *t*-BuLi (1.6 M in pentane, 9.6 mL, 15.31 mmol) was added very carefully under an N_2 atmosphere at −78 °C. The reaction mixture was allowed to warm up to 0 °C and stirred at this temperature for 2 h. Extra freshly distilled Et_2O (20 mL) was added, and then the reaction mixture was cooled down to −78 °C again and at this temperature dimethylchlorosilane (1.0 mL, 8.93 mmol) was added, after which the reaction mixture was allowed to warm up to rt and stirred at this temperature for 30 min. Then the reaction mixture was quenched with H_2O, extra Et_2O was added and the layers were separated. The organic layer was washed once with H_2O and brine, dried over Na_2SO_4, filtered and concentrated. Column chromatography using hexanes, 40/1 – 20/1 hexanes/CH_2Cl_2 gave product **53** in 68% yield (1.16 g, 1.72 mmol). ^{1}H NMR (300 MHz, $CDCl_3$) δ 0.17 (d, 6H, *J* = 3.6 Hz, Si(CH_3)$_2$), 1.05 (m, 63H, 3 × TIPS), 3.82 (dd, 1H, *J* = 11.1, 4.5 Hz, C_6H), 3.87 (m, 1H, C_3H), 4.00 (m, 3H, C_4H, C_6H, SiH), 4.18 (m, 1H, C_5H), 5.11 (dd, 1H, *J* = 5.1, 1.8 Hz, C_2H); ^{13}C NMR (75 MHz, $CDCl_3$) δ −5.3 (Si(*C*H_3)$_2$H), 12.1, 12.4, 12.6 (Si*C*H(CH_3)$_2$), 18.0 (SiCH(*C*H_3)$_2$), 62.2 (C_6H_2), 64.9, 70.1, 80.3 (C_3H, C_4H, C_5H), 110.5 (C_2H), 156.7 (C_1).

1-*C*-Dimethylhydroxysilyl-3,4,6-tri-*O*-(triisopropylsilyl)-D-glucal (54)

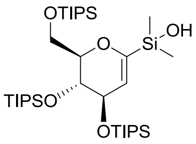

Compound **53** (1.16 g, 1.72 mmol) was dissolved in benzene (6 mL). In another flask di-μ-chlorobis[(*p*-cymene)chlororuthenium(II)] (32 mg, 0.05 mmol) was dissolved in acetonitrile (9 mL) and benzene (3 mL), followed by the addition of H_2O (62 μL, 3.45 mmol). The solution of compound **53** was added dropwise to this solution. This reaction mixture was stirred for 2 h open to air at rt. Then, extra di-μ-chlorobis[(*p*-cymene)chlororuthenium(II)] (16 mg, 0.025 mmol) and H_2O (31 μL, 1.73 mmol) were added. The reaction mixture was stirred for 1 h open to air at rt, after which is was diluted with H_2O and extracted with EtOAc. The organic layer was washed once with brine, dried over Na_2SO_4, filtered and concentrated. Column chromatography using hexanes, 9/1 CH_2Cl_2-hexanes gave product **54** in 80% yield (940 mg, 1.36 mmol). ^{1}H NMR (300 MHz, $CDCl_3$) δ 0.24 (d, 6H, J = 1.2 Hz, $Si(CH_3)_2$), 1.06 (m, 63H, 3 × TIPS), 3.79 (dd, 1H, J = 11.1, 4.2 Hz, C_6H), 3.89 (m, 1H, C_3H), 4.02 (m, 2H, C_4H, C_6H), 4.20 (m, 1H, C_5H), 5.16 (dd, 1H, J = 5.4, 1.8 Hz, C_2H); ^{13}C NMR (75 MHz, $CDCl_3$) δ −1.5, −1.1 ($Si(\mathit{C}H_3)_2H$), 12.0, 12.4, 12.5 ($Si\mathit{C}H(CH_3)_2$), 18.0, 18.1 ($SiCH(\mathit{C}H_3)_2$), 61.9 (C_6H_2), 64.7, 70.2, 80.0 (C_3H, C_4H, C_5H), 109.7 (C_2H), 157.2 (C_1).

Coupling product 55

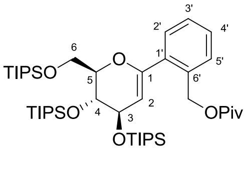

2-Iodobenzyl pivalate (**14**, 636 mg, 2.0 mmol), sodium *tert*-butoxide (385 mg, 4.0 mmol) and $Pd_2(dba)_3 \cdot CHCl_3$ (105 mg, 0.1 mmol) were placed in a flask. This was placed under an argon atmosphere, after which dry and degassed toluene (5 mL) was added to give a red-purple suspension. In another flask, compound **54** (1.38 g, 2.0 mmol) was dissolved in dry and degassed toluene (5 mL) and this was added via syringe to the

suspension. The reaction mixture was stirred for 20 h at 50 °C after which it was diluted with H_2O and EtOAc. The layers were separated and the organic layer was washed once with 10% aq solution of 2-dimethylaminoethanethiol and once with brine, dried over Na_2SO_4, filtered and concentrated. Column chromatography using a small amount of CH_2Cl_2 to dissolve the product and then hexanes, 50/1 to 40/1 hexanes/EtOAc gave product **55** in 53% yield (853 mg, 1.06 mmol). ^{1}H NMR (300 MHz, $CDCl_3$) δ 1.08 (m, 63H, 3 × TIPS), 1.22 (s, 9H, Piv), 3.97 (dd, 1H, *J* = 11.1, 4.5 Hz, C_6H), 4.17 (m, 2H, C_4H, C_6H), 4.43 (m, 1H, C_5H), 5.01 (dd, 1H, *J* = 5.4, 1.5 Hz, C_3H), 5.11 (s, 1H, C_2H), 5.32 (dd, 2H, *J* = 25.5, 13.2 Hz, CH_2OPiv), 7.34 (m, 4H, C_2'H, C_3'H, C_4'H, C_5'H); ^{13}C NMR (75 MHz, $CDCl_3$) δ 12.0, 12.4 (Si*C*H(CH_3)$_2$), 18.0, 18.1 (SiCH($\mathit{C}H_3$)$_2$), 27.2 (C($\mathit{C}H_3$)$_3$ from Piv), 38.8 (*C*(CH_3)$_3$ from Piv), 62.0 (CH_2OPiv), 63.5 (C_6H_2), 66.2, 69.3 (C_3H, C_5H), 81.7 (C_4H), 100.2 (C_2H), 127.3, 127.4, 128.5, 128.9 (C_2'H, C_3'H, C_4'H, C_5'H), 135.3, 136.3 (C_1', C_6'), 151.3 (C_1), 177.9 (C=O).

Numbering scheme used in the NMR assignments of selected compounds (and their close relatives):

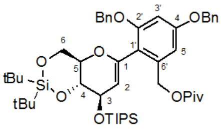

23#

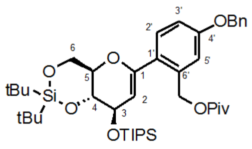

24#

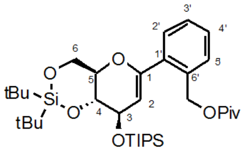

25#

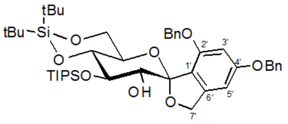

32α#

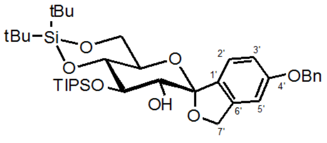

33α#

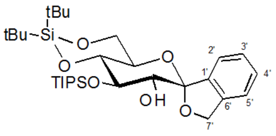

34α#

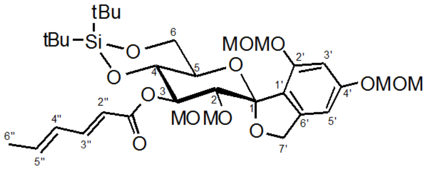

40#

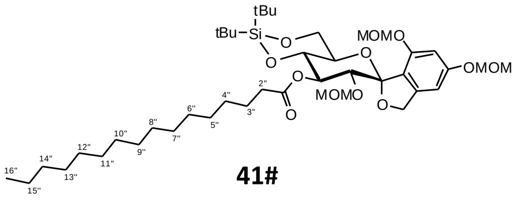

41#

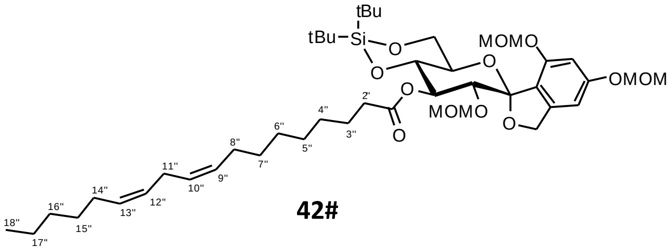

42#

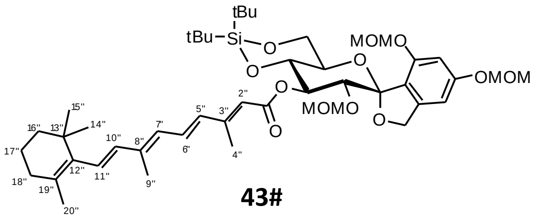

43#

D-Glucal (16)

Tri-*O*-acetyl-D-glucal (**15**, 8.16 g, 30.0 mmol) was dissolved in dry MeOH (85 mL), and NaOMe in MeOH (30% solution, 340 μL) was added. The reaction mixture was stirred at rt for 1 h and then some silica was added. This mixture was concentrated to dryness and placed on a silica column. Column chromatography using 5/1 EtOAc/EtOH gave product **16** in 96% yield (4.23 g, 28.93 mmol). ^{1}H NMR (300 MHz, CD_3OD) δ 3.56 (dd, 1H, J = 9.3, 6.9 Hz, C_4H), 3.79 (m, 3H, C_5H, 2 × C_6H), 4.11 (dt, 1H, J = 6.9, 2.1 Hz, C_3H), 4.68 (dd, 1H, J = 6.0, 2.4 Hz, C_2H), 6.34 (dd, 1H, J = 6.0, 1.5 Hz, C_1H); ^{13}C NMR (75 MHz, CD_3OD) δ 62.2 (C_6H_2), 70.5, 70.9 (C_3H, C_4H), 80.3 (C_5H), 104.5 (C_2H), 144.9 (C_1H).

4,6-*O*-Di-(*tert*-butyl)silanediyl-D-glucal (17)

D-Glucal (**16**, 3.64 g, 24.89 mmol) was dissolved in dry DMF (110 mL) and the solution was cooled down to −40 °C. Then, di-*tert*-butylsilyl bis(trifluoromethanesulfonate) (8.92 mL, 27.38 mmol) was added dropwise and this reaction mixture was stirred for 90 min at −40 °C. Pyridine (2.42 mL, 39.87 mmol) was added and the reaction mixture was allowed to warm up to rt over about 30 min. Then, the reaction mixture was diluted with Et_2O, washed with saturated aqueous $NaHCO_3$ and twice with H_2O, dried over Na_2SO_4, filtered and concentrated. Column chromatography using 15/1 hexanes/EtOAc gave product **17** in 90% yield (6.39 g, 22.31 mmol). ^{1}H NMR (300 MHz, $CDCl_3$) δ 1.00 (s, 9H, *t*-Bu), 1.07 (s, 9H, *t*-Bu), 2.41 (br s, 1H, OH), 3.89 (m, 3H, C_4H, C_5H, C_6H), 4.18 (dd, 1H, J = 9.9, 4.5 Hz, C_6H), 4.30 (m, 1H, C_3H), 4.76 (dd, 1H, J = 6.0, 1.8 Hz, C_2H), 6.27 (dd, 1H, J = 6.0, 1.8 Hz, C_1H); ^{13}C NMR (75 MHz, $CDCl_3$) δ 19.8, 22.7 (2 × $C(CH_3)_3$), 26.9, 27.4 (2 × $C(CH_3)_3$), 65.7 (C_6H_2), 70.2, 72.3 (C_3H, C_5H), 77.4 (C_4H), 103.0 (C_2H), 143.6 (C_1H).

3-*O*-Triethylsilyl-4,6-*O*-di-(*tert*-butyl)silanediyl-D-glucal (18)

Compound **17** (6.89 g, 24.05 mmol) was dissolved in dry CH_2Cl_2 (95 mL) and placed under a N_2 atmosphere. Then triethylsilyl chloride (5.0 mL, 30.06 mmol) and pyridine (2.9 mL, 36.07 mL) were added and this reaction mixture was stirred at rt overnight, after which it was diluted with 5% aq $NaHCO_3$. The aqueous layer was extracted with CH_2Cl_2 and the combined organic layers were washed with brine, dried over Na_2SO_4, filtered and concentrated. Column chromatography using 30/1 hexanes/EtOAC gave product **18** in 93% yield (8.97 g, 22.38 mmol). ^{1}H NMR (300 MHz, $CDCl_3$) δ 0.67 (m, 6H, $SiCH_2$), 0.99 (t, 9H, J = 7.8 Hz, $SiCH_2CH_3$), 1.00 (s, 9H, *t*-Bu), 1.06 (s, 9H, *t*-Bu), 3.80 (m, 1H, C_5H), 3.95 (m, 2H, C_4H, C_6H), 4.15 (dd, 1H, J = 10.5, 5.1 Hz, C_6H), 4.28 (dt, 1H, J = 6.9, 1.8 Hz, C_3H), 4.61 (dd, 1H, J = 6.0, 1.8 Hz, C_2H), 6.23 (dd, 1H, J = 6.0, 1.2 Hz, C_1H); ^{13}C NMR (75 MHz, $CDCl_3$) δ 4.8 ($SiCH_2$), 6.8 ($SiCH_2\mathit{C}H_3$), 19.8, 22.7 (2 × $\mathit{C}(CH_3)_3$), 26.9, 27.4 (2 × $C(\mathit{C}H_3)_3$), 65.9 (C_6H_2), 70.6 (C_3H), 72.8 (C_5H), 77.2 (C_4H), 105.2 (C_2H), 143.0 (C_1H).

3-*O*-Triisopropylsilyl-4,6-*O*-di-(*tert*-butyl)silanediyl-D-glucal (19)

Compound **17** (6.30g, 22.0 mmol) was dissolved in dry DMF (165 mL), and imidazole (3.75 g, 55.0 mmol) and triisopropylsilyl chloride (8.5 mL, 39.6 mmol) were added. This reaction mixture was heated to 60 °C and stirred at this temperature for 44 h. Then, the reaction mixture was cooled down to rt and stirred for an additional 24 h at rt. The resulting solution was concentrated and redissolved in Et_2O and H_2O. The layers were separated, the aqueous layer was extracted once with Et_2O, and the combined organic layers were washed with H_2O and brine, dried over Na_2SO_4, filtered and concentrated. Column chromatography twice using 40/1 hexanes/EtOAC gave product **19** in 74% yield (7.20 g, 16.26 mmol). ^{1}H NMR (300 MHz, $CDCl_3$) δ

1.05 (m, 39H, TIPS, 2 × *t*-Bu), 3.80 (m, 1H, C_5H), 3.98 (m, 2H, C_4H, C_6H), 4.16 (dd, 1H, *J* = 10.2, 5.1 Hz, C_6H), 4.42 (dt, 1H, *J* = 6.9, 1.8 Hz, C_3H), 4.67 (dd, 1H, *J* = 6.3, 1.8 Hz, C_2H), 6.22 (dd, 1H, *J* = 6.0, 1.2 Hz, C_1H); ^{13}C NMR (75 MHz, $CDCl_3$) δ 12.4 (Si*C*H(CH_3)$_2$), 18.1 (SiCH(*C*H_3)$_2$), 19.8, 22.8 (2 × *C*(CH_3)$_3$), 26.9, 27.5 (2 × C(*C*H_3)$_3$), 66.0 (C_6H_2), 70.8, 72.8 (C_3H, C_5H), 77.4 (C_4H), 105.4 (C_2H), 142.7 (C_1H).

1-*C*-Dimethylsilyl-3-*O*-triisopropylsilyl-4,6-*O*-di-(*tert*-butyl)silanediyl-D-glucal (21)

Compound **19** (5.85 g, 13.22 mmol) was dissolved in freshly distilled Et_2O (150 mL) and placed under an argon atmosphere. The solution was cooled down to −78 °C, and then *t*-BuLi (1.6 M in pentane, 50 mL, 79.27 mmol) was added very carefully under an argon atmosphere at −78 °C. The reaction mixture was allowed to warm up to 0 °C and stirred at this temperature for 2 h. Then, the reaction mixture was cooled down to −78° C again, and at this temperature dimethylchlorosilane (5.1 mL, 46.24 mmol) was added, after which the reaction mixture was allowed to warm up to rt and stirred at this temperature for 1 h. Then, the reaction mixture was quenched with H_2O, extra Et_2O was added and the layers were separated. The organic layer was washed once with H_2O, brine, dried over Na_2SO_4, filtered and concentrated. Column chromatography using hexanes, 50/1 hexanes/EtOAC gave product **21** in 99% yield (6.56 g, 13.10 mmol). ^{1}H NMR (300 MHz, $CDCl_3$) δ 0.16 (d, 6H, *J* = 3.6 Hz, Si(*C*H_3)$_2$H), 0.98 (s, 9H, *t*-Bu), 1.06 (s, 9H, *t*-Bu), 1.11 (m, 21H, TIPS), 3.74 (m, 1H, C_5H), 3.96 (m, 3H, C_4H, C_6H, SiH), 4.16 (dd, 1H, *J* = 10.2, 4.8 Hz, C_6H), 4.39 (dt, 1H, *J* = 7.8, 1.2 Hz, C_3H), 4.95 (d, 1H, *J* = 2.1 Hz, C_2H); ^{13}C NMR (75 MHz, $CDCl_3$) δ −5.5 (Si(*C*H_3)$_2$H) 12.5 (Si*C*H(CH_3)$_2$), 18.1 (SiCH(*C*H_3)$_2$), 19.8, 22.7 (2 × *C*(CH_3)$_3$), 26.9, 27.5 (2 × C(*C*H_3)$_3$), 66.2 (C_6H_2), 71.3, 73.1 (C_3H, C_5H), 115.8 (C_2H), 157.1 (C_1).

1-*C*-Dimethylhydroxysilyl-3-*O*-triisopropylsilyl-4,6-*O*-di-(*tert*-butyl)silanediyl-D-glucal (22)

Compound **21** (16.19 g, 32.32 mmol) was dissolved in benzene (125 mL). In another flask di-μ-chloro-bis[(*p*-cymene)chlororuthenium(II)] (595 mg, 0.97 mmol) was dissolved in acetonitrile (185 mL) and benzene (60 mL), followed by the addition of H_2O (1.16 mL, 64.64 mmol). The solution of compound **21** was added dropwise to this solution. This reaction mixture was stirred for 1 h while open to air at rt. Then, extra di-μ-chloro-bis[(*p*-cymene)chlororuthenium(II)] (198 mg, 0.32 mmol) and H_2O (1.16 mL, 64.64 mmol) were added. The reaction mixture was stirred for 3 h open to air at rt, after which is was diluted with H_2O and extracted with EtOAc. The organic layer was washed with brine, dried over Na_2SO_4, filtered and concentrated. Column chromatography using 9/1 hexanes/EtOAC gave product **22** in 95% yield (15.91 g, 30.78 mmol). ^{1}H NMR (300 MHz, $CDCl_3$) δ 0.23 (s, 6H, Si(CH_3)$_2$OH), 0.99 (s, 9H, *t*-Bu), 1.06 (s, 9H, *t*-Bu), 1.11 (m, 21H, TIPS), 1.78 (s, 1H, SiOH), 3.74 (m, 1H, C_5H), 3.95 (m, 2H, C_4H, C_6H), 4.17 (dd, 1H, *J* = 10.2, 4.8 Hz, C_6H), 4.40 (dd, 1H, *J* = 6.9, 1.8 Hz, C_3H), 5.01 (d, 1H, *J* = 1.8 Hz, C_2H); ^{13}C NMR (75 MHz, $CDCl_3$) δ −1.3 (Si(CH$_3$)$_2$OH), 12.4 (SiCH(CH_3)$_2$), 18.1 (SiCH(CH$_3$)$_2$), 19.8, 22.7 (2 × C(CH_3)$_3$), 26.9, 27.5 (2 × C(CH$_3$)$_3$), 66.2 (C_6H_2), 71.2, 73.0 (C_3H, C_5H), 115.2 (C_2H), 157.9 (C_1).

Coupling product 23

3,5-Dibenzyloxy-2-iodobenzyl pivalate (**12**, 1.06 g, 2.0 mmol), sodium *tert*-butoxide (385 mg, 4.0 mmol) and $Pd_2(dba)_3{\cdot}CHCl_3$ (105 mg, 0.1 mmol) were placed in a flask. This was placed under an argon atmosphere, after which dry and degassed toluene (5 mL) was added to give a red-purple suspension. In another flask, compound **22** (1.03 g, 2.0 mmol) was dissolved in dry and degassed toluene (5 mL) and this

solution was added via syringe to the suspension. The reaction mixture was stirred for 6 h at 50 °C after which it was stored at 4 °C overnight. The next day, the reaction mixture was filtered through hyflo and the hyflo pad was washed with EtOAc. The filtrate was washed with H_2O, twice with brine, dried over Na_2SO_4, filtered and concentrated. Column chromatography using a small amount of CH_2Cl_2 to dissolve the product and then hexanes, 20/1 hexanes/EtOAc gave product **23** in 72% yield (1.21g, 1.43 mmol). ^{1}H NMR (300 MHz, $CDCl_3$) δ 1.05 (s, 9H, *t*-Bu), 1.14 (m, 30H, TIPS, *t*-Bu), 1.26 (s, 9H, Piv), 4.01 (m, 2H, C_5H, C_6H), 4.18 (m, 2H, C_4H, C_6H), 4.59 (dd, 1H, J = 6.9, 2.4 Hz, C_3H), 4.85 (d, 1H, J = 2.4 Hz, C_2H), 5.06, 5.08 (2 × s, 2 × 2H, 2 × OCH_2Ph), 5.20 (d, 2H, J = 4.5 Hz, CH_2OPiv), 6.60 (d, 1H, J = 2.4 Hz, C_3'H or C_5'H), 6.65 (d, 1H, J = 2.4 Hz, C_3'H or C_5'H), 7.39 (m, 10H, 2 × C_6H_5); ^{13}C NMR (75 MHz, $CDCl_3$) δ 12.4 (Si*C*H(CH_3)$_2$), 18.2 (SiCH(*C*H_3)$_2$), 19.8, 22.7 (2 × *C*(CH_3)$_3$ from *t*-Bu), 27.0, 27.2, 27.5 (2 × C(*C*H_3)$_3$ from *t*-Bu, C(*C*H_3)$_3$ from Piv), 38.8 (*C*(CH_3)$_3$ from Piv), 63.6 (CH_2OPiv), 66.0 (C_6H_2), 70.1, 70.4 (2 × OCH_2Ph), 71.8 (C_3H), 73.1 (C_5H), 77.7 (C_4H), 100.5, 105.4 (C_3'H, C_5'H), 107.2 (C_2H), 117.5 (C_1'), 126.9, 127.4, 127.7, 128.0, 128.4, 128.6 (CH from Ph), 136.6, 136.9 (2 × C from Ph), 137.7 (C_6'), 146.7 (C_1), 158.0, 160.0 (C_3', C_5'), 178.0 (C=O).

Coupling product 24

Sodium *tert*-butoxide (385 mg, 4.0 mmol), 5-benzyloxy-2-iodobenzyl pivalate (**13**, 849 mg, 2.0 mmol), and $Pd_2(dba)_3 \cdot CHCl_3$ (105 mg, 0.1 mmol) were placed in a flask. This was placed under an argon atmosphere, after which dry and degassed toluene (5 mL) was added to give a red-purple suspension. In another flask, compound **22** (1.03 g, 2.0 mmol) was dissolved in dry and degassed toluene (5 mL) and this was added via syringe to the suspension. The reaction mixture was stirred for 20 h at

50 °C after which it was diluted with H_2O and EtOAc. The layers were separated and the organic layer was washed with 10% aqueous solution of 2-(dimethylamino)ethanethiol and with brine, dried over Na_2SO_4, filtered and concentrated. Column chromatography using 15/1 hexanes/EtOAc gave product **24** in 79% yield (1165 mg, 1.58 mmol). ^{1}H NMR (300 MHz, $CDCl_3$) δ 1.01 (s, 9H, *t*-Bu), 1.08 (s, 9H, *t*-Bu), 1.12 (m, 21H, TIPS), 1.22 (s, 9H, Piv), 4.10 (m, 4H, C_4H, C_5H, C_6H_2), 4.53 (dd, 1H, J = 6.9, 1.2 Hz, C_3H), 4.83 (d, 1H, J = 1.2 Hz, C_2H), 5.06 (s, 2H, OCH_2Ph), 5.16 (d, 2H, J = 7.8 Hz, CH_2OPiv), 6.87 (dd, 1H, J = 8.7, 2.1 Hz, C_3'H), 6.98 (d, 1H, J = 1.8 Hz, C_5'H), 7.35 (m, 6H, C_2'H, C_6H_5); ^{13}C NMR (75 MHz, $CDCl_3$) δ 12.4 (Si*C*H(CH_3)$_2$), 18.1 (SiCH(*C*H_3)$_2$), 19.8, 22.7 (2 × *C*(CH_3)$_3$ from *t*-Bu), 26.9, 27.3, 27.5 (2 × C(*C*H_3)$_3$ from *t*-Bu, C(*C*H_3)$_3$ from Piv), 38.9 (*C*(CH_3)$_3$ from Piv), 64.1 (CH_2OPiv), 66.0 (C_6H_2), 69.9 (OCH_2Ph), 71.6, 73.1 (C_3H, C_5H), 104.6 (C_2H), 113.6, 114.6 (C_3'H, C_5'H), 126.8 (C_1'), 127.4, 128.0, 128.6 (CH from Ph), 130.7 (C_2'H), 136.4, 136.6 (C from Ph, C_6'), 151.9 (C_1), 159.2, (C_4'), 178.1 (C=O).

Coupling product 25

2-Iodobenzyl pivalate (**14**, 636 mg, 2.0 mmol), sodium *tert*-butoxide (385 mg, 4.0 mmol) and $Pd_2(dba)_3 \cdot CHCl_3$ (105 mg, 0.1 mmol) were placed in a flask. This was placed under an argon atmosphere, after which dry and degassed toluene (5 mL) was added to give a red-purple suspension. In another flask, compound **22** (1.03 g, 2.0 mmol) was dissolved in dry and degassed toluene (5 mL) and this was added via syringe to the suspension. The reaction mixture was stirred for 20 h at 50 °C after which it was diluted with H_2O and EtOAc. The layers were separated and the organic layer was washed with 10% aqueous solution of 2-(dimethylamino)ethanethiol and with brine, dried over Na_2SO_4, filtered and concentrated. Column chromatography

using a small amount of CH_2Cl_2 to dissolve the product and then hexanes, 50/1 to 40/1 hexanes/EtOAc gave product **25** in 62% yield (780 mg, 1.23 mmol). ^{1}H NMR (300 MHz, $CDCl_3$) δ 1.02 (s, 9H, *t*-Bu), 1.08 (s, 9H, *t*-Bu), 1.12 (m, 21H, TIPS, *t*-Bu), 1.23 (s, 9H, Piv), 4.12 (m, 4H, C_4H, C_5H, C_6H_2), 4.56 (dd, 1H, *J* = 6.6, 2.4 Hz, C_3H), 4.89 (d, 1H, *J* = 2.4 Hz, C_2H), 5.20 (d, 2H, *J* = 5.7 Hz, CH_2OPiv), 7.32 (m, 4H, C_2'H, C_3'H, C_4'H, C_5'H); ^{13}C NMR (75 MHz, $CDCl_3$) δ 12.5 (Si*C*H(CH_3)$_2$), 18.2 (SiCH(CH_3)$_2$), 19.9, 22.8 (2 × *C*(CH_3)$_3$ from *t*-Bu), 27.0, 27.3, 27.5 (2 × C(CH_3)$_3$ from *t*-Bu, C(CH_3)$_3$ from Piv), 38.9 (*C*(CH_3)$_3$ from Piv), 64.3 (CH_2OPiv), 66.0 (C_6H_2), 71.6, 73.3 (C_3H, C_5H), 105.3 (C_2H), 127.9, 128.4, 129.0, 129.3 (C_2'H, C_3'H, C_4'H, C_5'H), 134.2, 134.8 (C_1', C_6'), 152.2 (C_1), 178.2 (C=O).

Product 26

Compound **23** (8.37 g, 9.90 mmol) was dissolved in dry CH_2Cl_2 (175 mL) and this solution was cooled down to −78 °C and placed under an argon atmosphere. DIBAL-H (1.0 M in hexanes, 20.8, mL, 20.80 mmol) was added and the reaction mixture was stirred for 5 min at −78 °C and then for 1 h at rt. Then, it was cooled down to 0 °C and carefully quenched with H_2O. This mixture was vigorously stirred at rt for 10 min after which a gel was formed. This gel was filtered through hyflo and the hyflo pad was washed with CH_2Cl_2. The layers were separated and the organic layer was dried over Na_2SO_4, filtered and concentrated. Column chromatography using 15/1 hexanes/EtOAc to 10/1 hexanes/EtOAc to 5/1 hexanes/EtOAc gave product **26** in 86% yield (6.47 g, 8.50 mmol). ^{1}H NMR (300 MHz, $CDCl_3$) δ 1.00 (s, 9H, *t*-Bu), 1.09 (m, 30H, TIPS, *t*-Bu), 3.97 (m, 2H, C_5H, C_6H), 4.13 (m, 2H, C_4H, C_6H), 4.55 (dd, 1H, *J* = 6.6, 2.1 Hz, C_3H), 4.65 (m, 2H, CH_2OH), 4.85 (d, 1H, *J* = 2.1 Hz, C_2H), 5.01, 5.04 (2 × s, 2 × 2H, 2 × OCH_2Ph), 6.53 (d, 1H, *J* = 2.1 Hz, C_3'H or C_5'H), 6.73 (d, 1H, *J* =

2.7 Hz, C_3'H or C_5'H), 7.35 (m, 10H, 2 × C_6H_5); ^{13}C NMR (75 MHz, $CDCl_3$) δ 12.4 (Si*C*H(CH_3)$_2$), 18.1 (SiCH(*C*H_3)$_2$), 19.8, 22.7 (2 × *C*(CH_3)$_3$), 27.0, 27.4 (2 × C(*C*H_3)$_3$), 63.3 (CH_2OH), 66.0 (C_6H_2), 70.1, 70.4 (2 × OCH_2Ph), 71.8 (C_3H), 73.2 (C_5H), 77.8 (C_4H), 100.3, 105.4 (C_3'H, C_5'H), 107.4 (C_2H), 116.8 (C_1'), 126.8, 127.4, 127.7, 128.0, 128.4, 128.6 (CH from Ph), 136.6, 136.9 (2 × C from Ph), 142.3 (C_6'), 147.0 (C_1), 157.9, 160.3 (C_2', C_4').

Product 27

Compound **24** (1165 mg, 1.58 mmol) was dissolved in dry CH_2Cl_2 (25 mL) and this solution was cooled down to −78 °C and placed under an argon atmosphere. DIBAL-H (1.0 M in hexanes, 3.31 mL, 3.31 mmol) was added and the reaction mixture was stirred for 5 min at −78 °C and then for 30 min at rt. Then, it was cooled down to 0 °C and hyflo (5 g) together with CH_2Cl_2 (7 mL) was added to the reaction mixture. Then, H_2O (1.8 mL) were added very carefully. This mixture was vigorously stirred at rt for 10 min after which a gel was formed. This gel was filtered through hyflo and the hyflo pad was washed with EtOAc. The filtrate was dried over Na_2SO_4, filtered and concentrated. Column chromatography using 15/1 to 10/1 hexanes/EtOAc gave product **27** in 70% yield (730 mg, 1.11 mmol). ^{1}H NMR (300 MHz, $CDCl_3$) δ 1.01 (s, 9H, *t*-Bu), 1.08 (s, 9H, *t*-Bu), 1.13 (m, 21H, TIPS), 4.09 (m, 4H, C_4H, C_5H, C_6H_2), 4.58 (m, 3H, CH_2OH, C_3H), 4.86 (s, 1H, C_2H), 5.08 (s, 2H, OCH_2Ph), 6.86 (d, 1H, J = 5.7 Hz, C_3'H), 7.09 (s, 1H, C_5'H), 7.34 (m, 6H, C_2'H, C_6H_5); ^{13}C NMR (75 MHz, $CDCl_3$) δ 12.4 (Si*C*H(CH_3)$_2$), 18.2 (SiCH(*C*H_3)$_2$), 19.9, 22.8 (2 × *C*(CH_3)$_3$ from *t*-Bu), 26.9, 27.4 (2 × C(*C*H_3)$_3$ from *t*-Bu), 63.8 (CH_2OH), 65.9 (C_6H_2), 70.0 (OCH_2Ph), 71.5, 73.2 (C_3H, C_5H), 104.9 (C_2H), 113.7, 115.0 (C_3'H, C_5'H), 126.7 (C_1'), 127.4, 128.0,

128.6 (CH from Ph), 130.7 (C_2'H), 136.6 (C from Ph), 140.8 (C_6'), 152.1 (C_1), 159.5 (C_4').

Product 28

Compound **25** (740 mg, 1.17 mmol) was dissolved in dry CH_2Cl_2 (19 mL) and this solution was cooled down to −78 °C and placed under an argon atmosphere. DIBAL-H (1.0 M in hexanes, 2.45, mL, 2.45 mmol) was added and the reaction mixture was stirred for 5 min at −78 °C and then for 30 min at rt. Then, it was cooled down to 0 °C and hyflo (4 g) together with CH_2Cl_2 (5 mL) was added to the reaction mixture. Then, H_2O (1.2 mL) was added very carefully. This mixture was vigorously stirred at rt for 10 min after which a gel was formed. This gel was filtered through hyflo and the hyflo pad was washed with EtOAc. The filtrate was dried over Na_2SO_4, filtered and concentrated, redissolved in hexanes and concentrated again. Column chromatography using hexanes, 15/1 hexanes/EtOAc gave product **28** in 75% yield (480 mg, 0.87 mmol). ^{1}H NMR (300 MHz, $CDCl_3$) δ 1.02 (s, 9H, *t*-Bu), 1.08 (s, 9H, *t*-Bu), 1.13 (m, 21H, TIPS, *t*-Bu), 4.13 (m, 4H, C_4H, C_5H, C_6H_2), 4.57 (dd, 1H, *J* = 6.6, 2.1 Hz, C_3H), 4.64 (m, 2H, CH_2OH), 4.93 (d, 1H, *J* = 2.1 Hz, C_2H), 7.36 (m, 4H, C_2'H, C_3'H, C_4'H, C_5'H); ^{13}C NMR (75 MHz, $CDCl_3$) δ 12.5 (Si*C*H($CH_3)_2$), 18.2 (SiCH($CH_3)_2$), 19.9, 22.8 (2 × *C*($CH_3)_3$ from *t*-Bu), 26.9, 27.5 (2 × C($CH_3)_3$ from *t*-Bu), 63.9 (CH_2OH), 65.9 (C_6H_2), 71.5, 73.4 (C_3H, C_5H), 105.6 (C_2H), 127.8, 129.0, 129.2, 129.4 (C_2'H, C_3'H, C_4'H, C_5'H), 134.2 (C_1'), 139.1 (C_6'), 152.4 (C_1).

Ring-closing products 29α and 29β

Compound **26** (1.79 g, 2.35 mmol) was dissolved in dry CH_2Cl_2 (55 mL) and $NaHCO_3$ (593 mg, 7.06 mmol) was added. This reaction mixture was cooled down to 0 °C and placed under an argon atmosphere. In another flask 3-chloroperoxybenzoic acid (70%, 696 mg, 2.82 mmol) was dissolved in dry CH_2Cl_2 (25 mL). This solution was dried by using Na_2SO_4 and filtered, and the filtrate was added via cannula to the reaction mixture. The reaction mixture was stirred for 5 min at 0 °C and then for 2 h at rt after which it was diluted with H_2O. The layers were separated, the aqueous layer was extracted with CH_2Cl_2 and the combined organic layers were washed with 1 M aq NaOH and brine, dried over Na_2SO_4, filtered and concentrated. This mixture of two anomers was directly used in the next reaction.

Ring-closing products 30α and 30β

Compound **27** (730 mg, 1.11 mmol) was dissolved in dry CH_2Cl_2 (25 mL) and $NaHCO_3$ (280 mg, 3.34 mmol) was added. This reaction mixture was cooled down to 0 °C and placed under an argon atmosphere. In another flask 3-chloroperoxybenzoic acid (70%, 330 mg, 1.34 mmol) was dissolved in dry CH_2Cl_2 (10 mL). This solution was dried by using Na_2SO_4 and filtered, and the filtrate was added via cannula to the reaction mixture. The reaction mixture was stirred for 5 min at 0 °C and then for 2 h at rt after which it was diluted with H_2O. The layers were separated, the aqueous layer was extracted 1 × CH_2Cl_2 and the combined organic layers were washed with 1 M aq NaOH, brine, dried over Na_2SO_4, filtered and concentrated. This mixture of two anomers was directly used in the next reaction.

Ring-closing products 31α and 31β

Compound **28** (480 mg, 0.87 mmol) was dissolved in dry CH_2Cl_2 (20 mL), and $NaHCO_3$ (220 mg, 2.62 mmol) was added. This reaction mixture was cooled down to 0 °C and put under an argon atmosphere. In another flask 3-chloroperoxybenzoic acid (70%, 260 mg, 1.05 mmol) was dissolved in dry CH_2Cl_2 (7 mL). This solution was dried by using Na_2SO_4 and filtered, and the filtrate was added via cannula to the reaction mixture. The reaction mixture was stirred for 5 min at 0 °C and then for 2.5 h at rt after which it was diluted with H_2O. The layers were separated, the aqueous layer was extracted with CH_2Cl_2 and the combined organic layers were washed with 1 M aq NaOH, brine, dried over Na_2SO_4, filtered and concentrated. This mixture of two anomers was directly used in the next reaction.

Isomerization product 32α

The crude mixture of two anomers (**29α**, **29β**, 2.35 mmol) was dissolved in dry $CHCl_3$ (30 mL), and hydrochloric acid (37% solution, 300 μL) was added. This reaction mixture was stirred at rt for 1 h after which it was diluted with H_2O. The layers were separated, the aqueous layer was extracted with CH_2Cl_2 and the combined organic layers were washed with brine, dried over Na_2SO_4, filtered and concentrated. Column chromatography using 9/1 hexanes/EtOAC gave product **32α** in 91% yield over two steps (1.66 g, 2.14 mmol). ^1H NMR (300 MHz, $CDCl_3$) δ 1.02 (s, 9H, *t*-Bu), 1.10 (m, 30H, TIPS, *t*-Bu), 3.82 (m, 2H, C_4H, C_6H), 3.97 (m, 2H, C_3H, C_5H), 4.11 (dd, 1H, J = 9.3, 4.5 Hz, C_6H), 4.34 (t, 1H, J = 8.4 Hz, C_2H), 5.09 (m, 6H, 2 × OCH_2Ph, $C_7'H_2$), 6.39 (d, 1H, J = 1.5 Hz, $C_3'H$ or $C_5'H$), 6.47 (d, 1H, J = 1.8 Hz, $C_3'H$ or $C_5'H$), 7.34 (m, 10H, 2 × C_6H_5); ^{13}C NMR (75 MHz, $CDCl_3$) δ 12.9 ($SiCH(CH_3)_2$), 18.4 ($SiCH(CH_3)_2$), 19.9, 22.8 (2 × $C(CH_3)_3$), 27.0, 27.5 (2 × $C(CH_3)_3$), 67.1 (C_6H_2), 68.8 (C_5H), 69.7,

70.4 (2 × OCH_2Ph), 73.1 ($C_7'H_2$), 73.6 (C_2H), 77.2 (C_3H), 78.2 (C_4H), 98.3, 100.4 (C_3'H, C_5'H), 110.8 (C_1), 118.2 (C_1'), 126.7, 127.3, 127.8, 128.0, 128.4, 128.6 (CH from Ph), 136.6 (2 × C from Ph), 143.3 (C_6'), 154.6, 162.0 (C_2', C_4'); ESIMS *m*/*z*: 777.65 $[M + H]^+$.

Isomerization product 33α

The crude mixture of two anomers (**30α**, **30β**, 1.11 mmol) was dissolved in dry $CHCl_3$ (15 mL), and hydrochloric acid (37% solution, 150 μL) was added. This reaction mixture was stirred at rt for 1 h after which it was diluted with H_2O. The layers were separated, the aqueous layer was extracted with CH_2Cl_2 and the combined organic layers were washed with brine, dried over Na_2SO_4, filtered and concentrated. Column chromatography using hexanes, 25/1 hexanes/EtOAC gave product **33α** in 83% yield over two steps (620 mg, 0.92 mmol). ^{1}H NMR (300 MHz, $CDCl_3$) δ 1.02 (s, 9H, *t*-Bu), 1.07 (s, 9H, *t*-Bu), 1.14 (m, 18H, $SiCH(CH_3)_2$), 1.21 (m, 3H, $SiCH(CH_3)_2$), 3.94 (m, 6H, C_2H, C_3H, C_4H, C_5H, C_6H_2), 5.05 (s, 2H, OCH_2Ph), 5.12 (dd, 2H, J = 30.9, 12.6 Hz, $C_7'H_2$), 6.82 (s, 1H, C_5'H), 6.95 (dd, 1H, J = 8.4, 2.1 Hz, C_3'H), 7.23 (d, 1H, J = 8.4 Hz, C_2'H), 7.36 (m, 5H, C_6H_5). ^{13}C NMR (75 MHz, $CDCl_3$) δ 12.9 ($SiCH(CH_3)_2$), 18.4, 18.5 ($SiCH(CH_3)_2$), 19.9, 22.8 (2 × $C(CH_3)_3$), 27.0, 27.5 (2 × $C(CH_3)_3$), 66.9 (C_6H_2), 69.0 (C_5H), 70.3 (OCH_2Ph), 72.6 ($C_7'H_2$), 75.1 (C_2H), 77.2 (C_3H), 78.2 (C_4H), 107.1 (C_5'H), 110.0 (C_1), 115.3 (C_3'H), 122.8 (C_2'H), 127.4, 128.0, 128.6 (CH from Ph), 130.3 (C_1'), 136.7 (C from Ph), 141.8 (C_6'), 160.3 (C_4').

Isomerization product 34α

The crude mixture of two anomers (**31α**, **31β**, 0.87 mmol) was dissolved in dry $CHCl_3$ (15 mL), and hydrochloric acid (37% solution, 150 μL) was added. This reaction

mixture was stirred at rt for 1 h after which it was diluted with H_2O. The layers were separated, the aqueous layer was extracted with CH_2Cl_2 and the combined organic layers were washed with brine, dried over Na_2SO_4, filtered and concentrated. Column chromatography using hexanes to 15/1 hexanes/EtOAC gave product **34α** in 86% yield over two steps (424 mg, 0.75 mmol). ^{1}H NMR (300 MHz, $CDCl_3$) δ 1.02 (s, 9H, *t*-Bu), 1.08 (s, 9H, *t*-Bu), 1.14 (m, 21H, TIPS), 3.95 (m, 6H, C_2H, C_3H, C_4H, C_5H, C_6H_2), 5.19 (dd, 2H, J = 27.9, 12.6 Hz, $C_7'H_2$), 7.31 (m, 4H, $C_2'H$, $C_3'H$, $C_4'H$, $C_5'H$); ^{13}C NMR (75 MHz, $CDCl_3$) δ 13.0 (Si*C*H(CH_3)$_2$), 18.4, 18.5 (SiCH(*C*H_3)$_2$), 20.0, 22.8 (2 × *C*(CH_3)$_3$), 27.0, 27.6 (2 × C(*C*H_3)$_3$), 66.8 (C_6H_2), 69.1 (C_5H), 73.0 ($C_7'H_2$), 75.1(C_2H), 77.2 (C_3H), 78.2 (C_4H), 110.2 (C_1), 121.1, 121.9, 128.1, 129.6 ($C_2'H$, $C_3'H$, $C_4'H$, $C_5'H$), 137.8 (C_1'), 139.9 (C_6').

Product of debenzylation, 35

Compound **32α** (734 mg, 0.94 mmol) was dissolved in dry THF (35 mL), then 10% palladium on carbon (365 mg, 50% w/w) and $NaHCO_3$ (515 mg, 6.14 mmol) were added and this reaction mixture was placed under a hydrogen atmosphere and stirred at rt for 1.5 h. The resulting mixture was filtered through hyflo and the hyflo pad was washed with CH_2Cl_2 and MeOH. The filtrate was concentrated and column chromatography using 2/1 hexanes/EtOAc gave product **35** in 98% yield (549 mg, 0.92 mmol). ^{1}H NMR (300 MHz, $CDCl_3$) δ 1.02 (s, 9H, *t*-Bu), 1.06 (s, 9H, *t*-Bu), 1.14 (m, 21H, TIPS), 2.32 (br s, 1H, C_2OH), 3.95 (m, 5H, C_3H, C_4H, C_5H, C_6H_2), 4.33 (t, 1H, J = 8.4 Hz, C_2H), 4.94 (d, 1H, J = 13.2 Hz, $C_7'H$), 5.06 (d, 1H, J = 12.9 Hz, $C_7'H$), 5.75 (s, 1H, $C_3'H$ or $C_5'H$), 5.98 (s, 1H, $C_3'H$ or $C_5'H$), 6.82 (br s, 1H, $C_2'OH$ or $C_4'OH$), 6.99 (br s, 1H, $C_2'OH$ or $C_4'OH$); ^{13}C NMR (75 MHz, $CDCl_3$) δ 13.0 (Si*C*H(CH_3)$_2$), 18.4 (SiCH(*C*H_3)$_2$), 19.9, 22.7 (2 × *C*(CH_3)$_3$), 27.0, 27.5 (2 × C(*C*H_3)$_3$),

66.7 (C_6H_2), 69.0 (C_5H), 73.0 (C_7'H_2), 73.7 (C_2H), 78.0 (C_4H), 100.0, 102.9 (C_3'H, C_5'H), 110.2 (C_1), 115.5 (C_1'), 143.4 (C_6'), 152.3, 158.4 (C_2', C_4'); ESIMS *m*/*z*: 597.40 $[M + H]^+$.

Product of MOM-protection, 36

Compound **35** (302 mg, 0.51 mmol) was dissolved in dry CH_2Cl_2 (7.5 mL) and methyl chloromethyl ether (770 µL, 10.1 mmol), DiPEA (2.64 mL, 15.2 mmol) and DMAP (20 mg, 0.15 mmol) were added. This reaction mixture was stirred at rt for 4 d, after which it was quenched with a half-saturated NH_4Cl solution (50 mL). The layers were separated, the H_2O layer was extracted twice with CH_2Cl_2, the combined organic layers were washed with brine, dried over Na_2SO_4, filtered and concentrated. Column chromatography using 9/1 hexanes/EtOAC gave product **36** in 78% yield (287 mg, 0.39 mmol). ^{1}H NMR (300 MHz, $CDCl_3$) δ 1.02 (s, 9H, *t*-Bu), 1.07 (s, 9H, *t*-Bu), 1.19 (m, 21H, TIPS), 2.53 (s, 3H, CH_2OCH_3), 3.43 (s, 3H, CH_2OCH_3), 3.52 (s, 3H, CH_2OCH_3), 3.80 (m, 2H, C_4H, C_6H), 3.97 (m, 1H, C_5H), 4.09 (m, 2H, C_3H, C_6H), 4.20 (t, 1H, *J* = 8.4 Hz, C_2H), 4.44 (d, 1H, *J* = 6.6 Hz, CH_2OCH_3), 4.71 (d, 1H, *J* = 6.6 Hz, CH_2OCH_3), 5.15 (m, 6H, 2 × CH_2OCH_3, C_7'H_2), 6.54 (s, 1H, C_3'H or C_5'H), 6.70 (s, 1H, C_3'H or C_5'H); ^{13}C NMR (75 MHz, $CDCl_3$) δ 13.7 ($SiCH(CH_3)_2$), 18.5 ($SiCH(CH_3)_2$), 19.9, 22.8 (2 × $C(CH_3)_3$), 27.1, 27.5 (2 × $C(CH_3)_3$), 54.5, 55.8, 56.2 (3 × CH_2OCH_3), 67.0 (C_6H_2), 68.4 (C_5H), 73.0 (C_7'H_2), 78.7 (C_4H), 80.8 (C_3H), 94.5, 94.7, 98.4 (3 × CH_2OCH_3), 100.8, 103.1 (C_3'H, C_5'H), 110.6 (C_1), 120.1 (C_1'), 143.5 (C_6'), 154.1, 160.1 (C_2', C_4'); ESIMS *m*/*z*: 729.30 $[M + H]^+$.

Deprotection with TBAHF, 37

Compound **36** (1.23 g, 1.69 mmol) was dissolved in TBAHF (1.0 M in THF, 100 mL). This reaction mixture was stirred at rt for 2 d after which it was diluted with Et_2O, washed twice with 1 M aq NaOH, dried over Na_2SO_4, filtered and concentrated. Column chromatography using hexanes, 2/1 to 1/1 hexanes/EtOAc gave product **37** in 84% yield (835 mg, 1.42 mmol). ^{1}H NMR (300 MHz, $CDCl_3$) δ 1.13 (m, 21H, TIPS), 2.56 (s, 3H, CH_2OCH_3), 3.45 (s, 3H, CH_2OCH_3), 3.51 (s, 3H, CH_2OCH_3), 3.79 (m, 4H, C_4H, C_5H, C_6H_2), 4.10 (d, 1H, *J* = 9.3 Hz, C_3H), 4.23 (t, 1H, *J* = 9.3 Hz, C_2H), 4.45 (d, 1H, *J* = 6.6 Hz, CH_2OCH_3), 4.67 (d, 1H, *J* = 6.6 Hz, CH_2OCH_3), 5.17 (m, 6H, 2 × CH_2OCH_3, C_7'H_2), 6.56 (s, 1H, C_3'H or C_5'H), 6.72 (s, 1H, C_3'H or C_5'H); ^{13}C NMR (75 MHz, $CDCl_3$) δ 13.3 (SiCH(CH_3)$_2$), 18.2 (SiCH(CH_3)$_2$), 54.5, 55.8, 56.2 (3 × CH_2OCH_3), 62.2 (C_6H_2), 71.7 (C_4H or C_5H), 72.9 (C_7'H_2), 73.1 (C_4H or C_5H), 76.5 (C_2H), 80.2 (C_3H), 94.5, 94.9, 98.2 (3 × CH_2OCH_3), 100.9, 103.0 (C_3'H, C_5'H), 110.6 (C_1), 120.3 (C_1'), 143.4 (C_6'), 154.1, 160.0 (C_2', C_4'); ESIMS *m/z*: 611.20 $[M + Na]^+$.

Deprotection with TBAF, 38

Compound **37** (1.15 g, 1.95 mmol) was dissolved in THF (20 mL). A solution of TBAF·$3H_2O$ in THF (1.0 M, 25 mL) was added and this reaction mixture was stirred at rt for 3 d. Concentration, followed by two times column chromatography using CH_2Cl_2, 5% MeOH in CH_2Cl_2 to 10% MeOH in CH_2Cl_2 gave product **38**, which still contained a small amount of TBAF. ^{1}H NMR (300 MHz, $CDCl_3$) δ 3.09 (s, 3H, CH_2OCH_3), 3.46 (s, 3H, CH_2OCH_3), 3.49 (s, 3H, CH_2OCH_3), 3.71 (m, 1H, C_6H), 3.81 (m, 2H, C_4H, C_5H), 3.89 (m, 1H, C_6H), 3.98 (t, 1H, *J* = 9.3 Hz, C_3H), 4.22 (d, 1H, *J* = 9.3 Hz, C_2H), 4.49 (d, 1H, *J* = 6.6 Hz, CH_2OCH_3), 4.61 (d, 1H, *J* = 6.6 Hz, CH_2OCH_3), 5.11 (m, 6H, 2 × CH_2OCH_3, C_7'H_2), 6.58 (s, 1H, C_3'H or C_5'H), 6.70 (s, 1H, C_3'H or

C_5'H); ^{13}C NMR (75 MHz, $CDCl_3$) δ 55.6, 56.1, 56.4 (3 × CH_2OCH_3), 62.3 (C_6H_2), 71.0 (C_5H), 72.8 (C_7'H_2), 73.0, 73.8 (C_3H, C_4H), 80.6 (C_2H), 94.6, 94.7, 97.6 (3 × CH_2OCH_3), 101.3, 103.0 (C_3'H, C_5'H), 109.8 (C_1), 119.6 (C_1'), 143.7 (C_6'), 153.3, 160.2 (C_2', C_4'); ESIMS *m*/*z*: 455.10 $[M + Na]^+$

Product of *t*-Bu_2Si protection, 39

Compound **38** (1.95 mmol) was dissolved in dry DMF (9 mL). This solution was cooled down to −40 °C, then di-*tert*-butylsilyl bis(trifluoromethanesulfonate) (700 μL, 2.15 mmol) was added dropwise. This reaction mixture was stirred for 90 min at −40 °C. Pyridine (190 μL, 2.34 mmol) was added slowly at this temperature after which the reaction mixture was allowed to warm up to rt over about 30 min. Afterwards, the reaction mixture was diluted with Et_2O, washed with saturated aqueous $NaHCO_3$ and twice with H_2O, dried over Na_2SO_4, filtered and concentrated. Column chromatography using 2/1 hexanes/EtOAc gave product **39** in 84% yield over two steps (943 mg, 1.65 mmol). ^1H NMR (300 MHz, $CDCl_3$) δ 1.02 (s, 9H, *t*-Bu), 1.08 (s, 9H, *t*-Bu), 3.11 (s, 3H, CH_2OCH_3), 3.45 (s, 3H, CH_2OCH_3), 3.48 (s, 3H, CH_2OCH_3), 3.82 (m, 2H, C_4H, C_6H), 3.99 (m, 2H, C_3H, C_5H), 4.09 (m, 1H, C_6H), 4.31 (d, 1H, J = 9.6 Hz, C_2H), 4.49 (d, 1H, J = 6.9 Hz, CH_2OCH_3), 4.60 (d, 1H, J = 6.9 Hz, CH_2OCH_3), 5.12 (m, 6H, 2 × CH_2OCH_3, C_7'H_2), 6.57 (s, 1H, C_3'H or C_5'H), 6.69 (s, 1H, C_3'H or C_5'H); ^{13}C NMR (75 MHz, $CDCl_3$) δ 20.0, 22.7 (2 × $C(CH_3)_3$), 27.1, 27.5 (2 × $C(CH_3)_3$), 55.5, 56.0 56.4 (3 × CH_2OCH_3), 66.9 (C_6H_2), 68.0 (C_5H), 73.1 (C_7'H_2), 74.1 (C_3H), 77.6 (C_4H), 79.6 (C_2H), 94.5, 97.5 (3 × CH_2OCH_3), 101.2, 102.9 (C_3'H, C_5'H), 110.0 (C_1), 119.2 (C_1'), 143.7 (C_6'), 153.3, 160.3 (C_2', C_4'); ESIMS *m*/*z*: 595.20 $[M + Na]^+$.

Coupling with sorbic acid, 40

A Schlenk flask was dried in the oven at 150 °C and cooled down under an argon atmosphere. To this Schlenk flask was added sorbic acid (45 mg, 0.40 mmol) and this was dissolved in dry toluene (6 mL). To this solution was added NEt_3 (474 µL, 3.40 mmol) and 2,4,6-trichlorobenzoyl chloride (135 µL, 0.87 mmol) and this reaction mixture was stirred at rt for 1 h. Compound **39** (177 mg, 0.31 mmol) and DMAP (98 mg, 0.80 mmol) were dissolved in dry toluene (5 mL) and this solution was added to the reaction mixture in the Schlenk flask, which gave a white suspension. This suspension was stirred at rt for 3 h after which it turned yellow. Afterwards the reaction mixture was diluted with toluene and saturated aqueous $NaHCO_3$. The layers were separated, the aqueous layer was extracted once with toluene and the combined organic layers were washed once with H_2O, once with brine, dried over Na_2SO_4, filtered and concentrated. Column chromatography using 4/1 hexanes/EtOAc gave product **40** in 89% yield (183 mg, 0.27 mmol). ^{1}H NMR (300 MHz, $CDCl_3$) δ 1.00 (s, 9H, *t*-Bu), 1.02 (s, 9H, *t*-Bu), 1.85 (d, 3H, *J* = 5.7 Hz, C_6''H_3), 2.85 (s, 3H, CH_2OCH_3), 3.45 (s, 3H, CH_2OCH_3), 3.52 (s, 3H, CH_2OCH_3), 3.87 (m, 2H, C_4H, C_6H), 4.11 (m, 2H, C_5H, C_6H), 4.38 (m, 3H, CH_2OCH_3, C_2H), 5.17 (m, 6H, 2 × CH_2OCH_3, C_7'H_2), 5.56 (t, 1H, *J* = 9.6 Hz, C_3H), 5.84 (d, 1H, *J* = 15.6 Hz, C_2''H), 6.17 (m, 2H, C_4''H, C_5''H), 6.57 (s, 1H, C_3'H or C_5'H), 6.70 (s, 1H, C_3'H or C_5'H), 7.32 (m, 1H, C_3''H); ^{13}C NMR (75 MHz, $CDCl_3$) δ 18.6 (C_6''H_3), 20.0, 22.6 (2 × $C(CH_3)_3$), 26.9, 27.4 (2 × $C(CH_3)_3$), 55.2, 56.0 56.3 (3 × CH_2OCH_3), 66.9 (C_6H_2), 68.4 (C_5H), 73.3 (C_7'H_2), 74.6 (C_3H), 75.8 (C_2H), 76.4 (C_4H), 94.5, 96.7 (3 × CH_2OCH_3), 101.1, 102.9 (C_3'H, C_5'H), 110.3 (C_1), 119.0 (C_2''H), 119.0 (C_1'), 129.9 (C_4''H), 139.0 (C_5''H), 143.8 (C_6'), 144.9 (C_3''H), 153.5, 160.4 (C_2', C_4'), 166.2 (C=O); ESIMS *m*/*z*: 667.80 $[M + H]^+$.

Coupling with palmitic acid, 41

A Schlenk flask was dried in the oven at 150 °C and cooled down under an argon atmosphere. To this Schlenk flask was added palmitic acid (110 mg, 0.43 mmol) and this was dissolved in dry toluene (6 mL). To this solution was added NEt_3 (505 μL, 3.62 mmol) and 2,4,6 trichlorobenzoyl chloride (144 μL, 0.92 mmol) and this reaction mixture was stirred at rt for 1 h. Compound **39** (189 mg, 0.33 mmol) and DMAP (105 mg, 0.86 mmol) were dissolved in dry toluene (5 mL) and this solution was added to the reaction mixture in the Schlenk flask, which gave a white suspension. This suspension was stirred at rt for 3 h after which it turned yellow. Afterwards the reaction mixture was diluted with toluene and saturated aqueous $NaHCO_3$. The layers were separated, the aqueous layer was extracted with toluene and the combined organic layers were washed with H_2O and with brine, dried over Na_2SO_4, filtered and concentrated. Column chromatography using 4/1 hexanes/EtOAc gave product **41** in quantitative yield (267 mg, 0.33 mmol). ^{1}H NMR (300 MHz, $CDCl_3$) δ 0.87 (t, 3H, *J* = 6.9 Hz, C_{16}''H_3), 1.00 (s, 9H, *t*-Bu), 1.03 (s, 9H, *t*-Bu), 1.25 (s, 24H, 12 × CH_2 4''-15''), 1.66 (m, 2H, C_3''H_2), 2.35 (m, 2H, C_2''H_2), 2.85 (s, 3H, CH_2OCH_3), 3.44 (s, 3H, CH_2OCH_3), 3.50 (s, 3H, CH_2OCH_3), 3.84 (m, 2H, C_4H, C_6H), 4.09 (m, 2H, C_5H, C_6H), 4.40 (m, 3H, CH_2OCH_3, C_2H), 5.16 (m, 6H, 2 × CH_2OCH_3, C_7'H_2), 5.50 (t, 1H, *J* = 9.6 Hz, C_3H), 6.56 (s, 1H, C_3'H or C_5'H), 6.70 (s, 1H, C_3'H or C_5'H); ^{13}C NMR (75 MHz, $CDCl_3$) δ 14.1 (C_{16}''H_3), 20.0 ($C(CH_3)_3$), 22.6 ($C(CH_3)_3$, C_{15}''H_2), 25.3 (C_3''H_2), 26.9, 27.4 (2 × $C(CH_3)_3$), 29.3, 29.6 (10 × CH_2 4''-13''), 31.9 (C_{14}''H_2), 34.7 (C_2''H_2), 55.1, 56.0 56.3 (3 × CH_2OCH_3), 66.8 (C_6H_2), 68.4 (C_5H), 73.2 (C_7'H_2), 74.5 (C_3H), 75.8 (C_2H), 76.5 (C_4H), 94.5, 96.9 (3 × CH_2OCH_3), 101.1, 102.9 (C_3'H, C_5'H), 110.3 (C_1), 119.0 (C_1'), 143.8 (C_6'), 153.6, 160.4 (C_2', C_4'), 172.5 (C=O); ESIMS *m*/*z*: 811.85 $[M + H]^+$, 833.25 $[M + Na]^+$.

Coupling with linoleic acid, 42

A Schlenk flask was dried in the oven at 150 °C and cooled down under an argon atmosphere. To this Schlenk flask was added linoleic acid (129 µL, 0.42 mmol) and this was dissolved in dry toluene (6 mL). To this solution was added NEt_3 (490 µL, 3.52 mmol) and 2,4,6-trichlorobenzoyl chloride (140 µL, 0.90 mmol) and this reaction mixture was stirred at rt for 1 h. Compound **39** (183 mg, 0.32 mmol) and DMAP (102 mg, 0.83 mmol) were dissolved in dry toluene (5 mL) and this solution was added to the reaction mixture in the Schlenk flask, which gave a white suspension. This suspension was stirred at rt for 3 h after which the suspension had become yellow. After 1 h, extra toluene (5 mL) was added to keep the reaction mixture soluble. Afterwards the reaction mixture was diluted with toluene and saturated aqueous $NaHCO_3$. The layers were separated, the aqueous layer was extracted once with toluene and the combined organic layers were washed once with H_2O, once with brine, dried over Na_2SO_4, filtered and concentrated. Column chromatography using 4/1 hexanes/EtOAc gave product **42** in 92% yield (247 mg, 0.30 mmol). ^{1}H NMR (300 MHz, $CDCl_3$) δ 0.89 (t, 3H, J = 6.6 Hz, C_{18}''H_3), 1.00 (s, 9H, *t*-Bu), 1.04 (s, 9H, *t*-Bu), 1.30 (m, 14H, 7 × CH_2 4''-7'', 15''-17''), 1.67 (m, 2H, C_3''H_2), 2.04 (m, 4H, C_8''H_2, C_{14}''H_2), 2.35 (m, 2H, C_2''H_2), 2.77 (t, 2H, J = 6.0 Hz, C_{11}''H_2), 2.85 (s, 3H, CH_2OCH_3), 3.44 (s, 3H, CH_2OCH_3), 3.50 (s, 3H, CH_2OCH_3), 3.86 (m, 2H, C_4H, C_6H), 4.09 (m, 2H, C_5H, C_6H), 4.39 (m, 3H, CH_2OCH_3, C_2H), 5.16 (m, 6H, 2 × CH_2OCH_3, C_7'H_2), 5.35 (m, 4H, C_9''H, C_{10}''H, C_{12}''H, C_{13}''H), 5.50 (t, 1H, J = 9.6 Hz, C_3H), 6.57 (s, 1H, C_3'H or C_5'H), 6.70 (s, 1H, C_3'H or C_5'H); ^{13}C NMR (75 MHz, $CDCl_3$) δ 14.0 (C_{18}''H_3), 19.9 ($C(CH_3)_3$), 22.5, 22.6 ($C(CH_3)_3$, C_{17}''H_2), 25.3, 25.5 (C_3''H_2,C_{11}''H_2), 26.8 ($C(CH_3)_3$), 27.1 (C_8''H_2, C_{14}''H_2), 27.4 ($C(CH_3)_3$), 29.0, 29.2, 29.3, 29.6 (C_4''H_2, C_5''H_2, C_6''H_2, C_7''H_2, C_{15}''H_2), 31.4 (C_{16}''H_2), 34.6 (C_2''H_2), 55.1,

56.0 56.2 (3 × CH_2OCH_3), 66.8 (C_6H_2), 68.4 (C_5H), 73.2 ($C_7'H_2$), 74.5 (C_3H), 75.8 (C_2H), 76.5 (C_4H), 94.5, 96.8 (3 × CH_2OCH_3), 101.1, 102.9 ($C_3'H$, $C_5'H$), 110.3 (C_1), 119.0 (C_1'), 127.8, 128.0 ($C_{10}''H$, $C_{12}''H$), 130.0, 130.1 ($C_9''H$, $C_{13}''H$), 143.8 (C_6'), 153.5, 160.4 (C_2', C_4'), 172.5 (C=O); ESIMS *m/z*: 836.15 $[M + H]^+$, 857.60 $[M + Na]^+$.

Coupling with all trans-retinoic acid, 43

A Schlenk flask was dried in the oven at 150 °C and cooled down under an argon atmosphere. To this Schlenk flask was added all-trans retinoic acid (103 mg, 0.34 mmol) and this was dissolved in dry toluene (5 mL). To this solution was added NEt_3 (403 μL, 2.89 mmol) and 2,4,6-trichlorobenzoyl chloride (115 μL, 0.74 mmol), and this reaction mixture was stirred at rt for 1 h. Compound **39** (150 mg, 0.26 mmol) and DMAP (83 mg, 0.68 mmol) were dissolved in dry toluene (4 mL) and this solution was added to the reaction mixture in the Schlenk flask, which gave directly a yellow suspension. This suspension was stirred at rt for 3 h. Afterwards the reaction mixture was diluted with toluene and saturated aqueous $NaHCO_3$. The layers were separated, the aqueous layer was extracted with toluene and the combined organic layers were washed with H_2O, with brine, dried over Na_2SO_4, filtered and concentrated. Column chromatography using 4/1 hexanes/EtOAc gave product **43** in 90% yield (201 mg, 0.24 mmol). ^1H NMR (300 MHz, $CDCl_3$) δ 1.02 (m, 24H, 2 × *t*-Bu, $C_{14}''H_3$, $C_{15}''H_3$), 1.47 (m, 2H, $C_{16}''H_2$), 1.61 (m, 2H, $C_{17}''H_2$), 1.71 (s, 3H, $C_{20}''H_3$), 2.00 (m, 5H, $C_9''H_3$, $C_{18}''H_2$), 2.36 (s, 3H, $C_4''H_3$), 2.86 (s, 3H, CH_2OCH_3), 3.44 (s, 3H, CH_2OCH_3), 3.51 (s, 3H, CH_2OCH_3), 3.87 (m, 2H, C_4H, C_6H), 4.11 (m, 2H, C_5H, C_6H), 4.40 (m, 3H, CH_2OCH_3, C_2H), 5.17 (m, 6H, 2 × CH_2OCH_3, $C_7'H_2$), 5.55 (t, 1H, J = 9.6 Hz, C_3H), 5.88 (s, 1H, $C_2''H$), 6.13, 6.29 (2 × m, 2 × 2H, $C_5''H$, $C_7''H$, $C_{10}''H$, $C_{11}''H$), 6.57 (s, 1H, $C_3'H$ or $C_5'H$), 6.70 (s, 1H, $C_3'H$ or $C_5'H$), 6.99 (m, 1H, $C_6''H$); ^{13}C

NMR (75 MHz, $CDCl_3$) δ 12.8 (C_4''H_3), 13.8 (C_9''H_3), 19.2 (C_{17}''H_2), 20.0 ($C(CH_3)_3$), 21.7 (C_{20}''H_3), 22.6 ($C(CH_3)_3$), 26.9, 27.3 (2 × $C(CH_3)_3$), 28.9 (C_{14}''H_3, C_{15}''H_3), 33.0 (C_{18}''H_2), 34.2 (C_{13}''), 39.5 (C_{16}''H_2), 55.1, 56.0 56.3 (3 × CH_2OCH_3), 66.9 (C_6H_2), 68.4 (C_5H), 73.2 (C_7'H_2), 73.9 (C_3H), 75.9 (C_2H), 76.5 (C_4H), 94.5, 96.8 (3 × CH_2OCH_3), 101.1, 102.9 (C_3'H, C_5'H), 110.3 (C_1), 118.6 (C_2''H), 119.0 (C_1'), 128.5 (C_6''H), 129.4 (C_{11}''H), 129.9 (C_{19}''), 130.7 (C_7''H), 135.1 (C_5''H), 137.2 (C_{10}''H), 137.6 (C_8''), 139.4 (C_{12}''), 143.8 (C_6'), 152.4 (C_3''), 153.5, 160.3 (C_2', C_4'), 165.9 (C=O); ESIMS *m*/*z*: 855.75 $[M + H]^+$.

Deprotection with TBAHF, 44

Compound **40** (183 mg, 0.27 mmol) was dissolved in TBAHF (1.0 M in THF, 16.5 mL). This reaction mixture was stirred at rt for 2 d after which it was diluted with Et_2O, washed twice with 1 M aq NaOH, dried over Na_2SO_4, filtered and concentrated. Fourfold column chromatography using 2/1 to 1/1 to 1/2 to 1/3 to 1/4 hexanes/EtOAc to remove all tributylamine and impurities gave product **44** in 25% yield (35 mg, 0.066 mmol). ^{1}H NMR (300 MHz, $CDCl_3$) δ 1.85 (d, 3H, *J* = 5.1 Hz, C_6''H_3), 3.17 (s, 3H, CH_2OCH_3), 3.46 (s, 6H, 2 × CH_2OCH_3), 4.01 (m, 2H, C_4H, C_6H), 4.28 (m, 2H, C_5H, C_6H), 4.55 (m, 3H, CH_2OCH_3, C_2H), 5.13 (m, 7H, 2 × CH_2OCH_3, C_7'H_2, C_3H), 5.71 (d, 1H, *J* = 15.3 Hz, C_2''H), 6.16 (m, 2H, C_4''H, C_5''H), 6.59 (s, 1H, C_3'H or C_5'H), 6.67 (s, 1H, C_3'H or C_5'H), 7.24 (m, 1H, C_3''H); ^{13}C NMR (75 MHz, $CDCl_3$) δ 18.6 (C_6''H_3), 55.6, 56.0 56.3 (3 × CH_2OCH_3), 63.1 (C_6H_2), 70.3 (C_5H), 71.8 (C_3H), 72.8 (C_7'H_2), 73.4 (C_2H), 80.4 (C_4H), 94.4, 94.5, 97.6 (3 × CH_2OCH_3), 101.4, 103.1 (C_3'H, C_5'H), 109.8 (C_1), 118.2 (C_2''H), 119.7 (C_1'), 129.6 (C_4''H), 139.9 (C_5''H), 143.7 (C_6'), 145.8 (C_3''H), 153.0, 160.1 (C_2', C_4'), 167.8 (C=O); ESIMS *m*/*z*: 527.45 $[M + H]^+$, 549.35 $[M + Na]^+$.

Deprotection with TBAHF, 45

Compound **41** (267 mg, 0.33 mmol) was dissolved in TBAHF (1.0 M in THF, 21 mL). This reaction mixture was stirred at rt for 2 d after which it was diluted with Et_2O, washed twice with 1 M aq NaOH, dried over Na_2SO_4, filtered and concentrated. Column chromatography using hexanes, 2/1 to 1/1 to 1/2 hexanes/EtOAc gave product **45** in 85% yield (188 mg, 0.28 mmol). ^{1}H NMR (300 MHz, $CDCl_3$) δ 0.88 (t, 3H, *J* = 6.6 Hz, C_{16}''H_3), 1.25 (s, 24H, 12 × CH_2 4''-15''), 1.65 (m, 2H, C_3''H_2), 2.39 (m, 2H, C_2''H_2), 2.80 (s, 3H, CH_2OCH_3), 3.45 (s, 3H, CH_2OCH_3), 3.51 (s, 3H, CH_2OCH_3), 3.80 (m, 3H, C_4H, C_5H, C_6H), 3.92 (m, 1H, C_6H), 4.39 (m, 2H, CH_2OCH_3, C_2H), 4.51 (d, 1H, *J* = 6.6 Hz, CH_2OCH_3), 5.16 (m, 6H, 2 × CH_2OCH_3, C_7'H_2), 5.37 (t, 1H, *J* = 9.6 Hz, C_3H), 6.58 (s, 1H, C_3'H or C_5'H), 6.72 (s, 1H, C_3'H or C_5'H); ^{13}C NMR (75 MHz, $CDCl_3$) δ 14.0 (C_{16}''H_3), 22.6 (C_{15}''H_2), 24.8 (C_3''H_2), 29.1, 29.3, 29.4, 29.6 (10 × CH_2 4''-13''), 31.8 (C_{14}''H_2), 34.4 (C_2''H_2), 55.0, 55.9, 56.3 (3 × CH_2OCH), 62.0 (C_6H_2), 70.1 (C_5H), 73.1 (C_7'H_2), 73.6 (C_3H), 76.2 (C_2H), 76.7 (C_4H), 94.5, 94.8, 97.0 (3 × CH_2OCH_3), 101.1, 103.0 (C_3'H, C_5'H), 109.9 (C_1), 119.1 (C_1'), 143.6 (C_6'), 153.7, 160.3 (C_2', C_4'), 175.0 (C=O); ESIMS *m*/*z*: 671.60 $[M + H]^+$.

Deprotection with TBAHF, 46

Compound **42** (247 mg, 0.30 mmol) was dissolved in TBAHF (1.0 M in THF, 18 mL). This reaction mixture was stirred at rt for 2 d after which it was diluted with Et_2O, washed twice with 1 M aq NaOH, dried over Na_2SO_4, filtered and concentrated. Column chromatography using hexanes, 2/1 to 1/1 to 1/2 hexanes/EtOAc gave product **46** in 86% yield (179 mg, 0.26 mmol). ^{1}H NMR (300 MHz, $CDCl_3$) δ 0.89 (t, 3H, *J* = 6.6 Hz, C_{18}''H_3), 1.31 (m, 14H, 7 × CH_2 4''-7'', 15''-17''), 1.65 (m, 2H, C_3''H_2), 2.04 (m, 4H, C_8''H_2, C_{14}''H_2), 2.38 (m, 2H, C_2''H_2), 2.77 (t, 2H, *J* = 6.0 Hz, C_{11}''H_2),

2.80 (s, 3H, CH_2OCH_3), 3.45 (s, 3H, CH_2OCH_3), 3.51 (s, 3H, CH_2OCH_3), 3.80 (m, 3H, C_4H, C_5H, C_6H), 3.93 (m, 1H, C_6H), 4.39 (m, 2H, CH_2OCH_3, C_2H), 4.51 (d, 1H, J = 6.9 Hz, CH_2OCH_3), 5.16 (m, 6H, 2 × CH_2OCH_3, $C_7'H_2$), 5.34 (m, 5H, $C_9''H$, $C_{10}''H$, $C_{12}''H$, $C_{13}''H$, C_3H), 6.58 (s, 1H, $C_3'H$ or $C_5'H$), 6.73 (s, 1H, $C_3'H$ or $C_5'H$); ^{13}C NMR (75 MHz, $CDCl_3$) δ 14.0 ($C_{18}''H_3$), 22.5 ($C_{17}''H_2$), 24.8, 25.5 ($C_3''H_2$,$C_{11}''H_2$), 27.1 ($C_8''H_2$, $C_{14}''H_2$), 29.0, 29.2, 29.5 ($C_4''H_2$, $C_5''H_2$, $C_6''H_2$, $C_7''H_2$, $C_{15}''H_2$), 31.4 ($C_{16}''H_2$), 34.4 ($C_2''H_2$), 55.0, 55.9, 56.3 (3 × CH_2OCH_3), 62.0 (C_6H_2), 70.1 (C_5H), 73.1 ($C_7'H_2$), 73.6 (C_3H), 76.2 (C_2H), 76.7 (C_4H), 94.5, 94.8, 97.0 (3 × CH_2OCH_3), 101.1, 102.9 ($C_3'H$, $C_5'H$), 109.9 (C_1), 119.1 (C_1'), 127.8, 127.9 ($C_{10}''H$, $C_{12}''H$), 129.9, 130.1 ($C_9''H$, $C_{13}''H$), 143.6 (C_6'), 153.7, 160.3 (C_2', C_4'), 174.9 (C=O); ESIMS m/z: 695.55 $[M + H]^+$, 857.60 $[M + Na]^+$.

Deprotection with TBAHF, 47

Compound **43** (186 mg, 0.22 mmol) was dissolved in TBAHF (1.0 M in THF, 14 mL). This reaction mixture was stirred at rt for 3 d after which it was diluted with Et_2O, washed twice with 1 M aq NaOH, dried over Na_2SO_4, filtered and concentrated. Fourfold column chromatography using 2/1 to 1/1 to 1/2 hexanes/EtOAc to remove all tributylamine and impurities gave product **47** in 39% yield (61 mg, 0.085 mmol). ^1H NMR (300 MHz, $CDCl_3$) δ 1.03 (m, 6H, $C_{14}''H_3$, $C_{15}''H_3$), 1.47 (m, 2H, $C_{16}''H_2$), 1.63 (m, 2H, $C_{17}''H_2$), 1.71 (s, 3H, $C_{20}''H_3$), 2.01 (m, 6H, $C_9''H_3$, $C_4''H_2$), 2.37 (s, 2H, $C_{18}''H_3$), 2.83 (s, 3H, CH_2OCH_3), 3.46 (s, 3H, CH_2OCH_3), 3.51 (s, 3H, CH_2OCH_3), 3.81 (m, 3H, C_4H, C_5H, C_6H), 3.97 (m, 1H, C_6H), 4.44 (m, 3H, CH_2OCH_3, C_2H), 5.15 (m, 6H, 2 × CH_2OCH_3, $C_7'H_2$), 5.39 (t, 1H, J = 9.6 Hz, C_3H), 5.84 (s, 1H, $C_2''H$), 6.19 (m, 4H, $C_5''H$, $C_7''H$, $C_{10}''H$, $C_{11}''H$), 6.58 (s, 1H, $C_3'H$ or $C_5'H$), 6.72 (s, 1H, $C_3'H$ or $C_5'H$), 7.05 (m, 1H, $C_6''H$); ^{13}C NMR (75 MHz, $CDCl_3$) δ 12.9 ($C_4''H_3$), 14.0 ($C_9''H_3$),

19.2 (C_{17}''H_2), 21.7 (C_{20}''H_3), 28.9 (C_{14}''H_3, C_{15}''H_3), 33.1 (C_{18}''H_2), 34.2 (C_{13}''), 39.6 (C_{16}''H_2), 55.3, 56.1 56.4 (3 × CH_2OCH_3), 62.3 (C_6H_2), 70.5 (C_5H), 73.2 (C_7'H_2), 73.6 (C_3H), 76.0 (C_2H or C_4H), 94.6, 94.8 97.0 (3 × CH_2OCH_3), 101.2, 103.0 (C_3'H, C_5'H), 110.0 (C_1), 117.2 (C_2''H), 119.2 (C_1'), 129.1, 129.3 (C_6''H, C_{11}''H), 129.9 (C_{19}''), 131.9 (C_7''H), 133.1 (C_5''H), 134.7 (C_{10}''H), 137.7 (C_8''), 140.3 (C_{12}''), 143.8 (C_6'), 155.1 (C_3''), 153.7, 160.4 (C_2', C_4'), 168.5 (C=O); ESIMS *m*/*z*: 715.60 $[M + H]^+$.

Sorbic acid mimic, 48

Compound **44** (35 mg, 0.066 mmol) was dissolved in dry MeOH (2.5 mL) and a small amount of Dowex 50 × 8 was added. This reaction mixture was stirred at 50 °C for 18 h after which it was filtered and concentrated. Compound **48** was obtained after preparative HPLC and lyophilization in 13% isolated yield (3.5 mg, 0.0089 mmol). ^1H NMR (300 MHz, CD_3OD) δ 1.85 (d, 3H, J = 5.7 Hz, C_6''H_3), 3.48 (t, 1H, J = 9.6 Hz, C_4H), 3.73 (t, 1H, J = 9.6 Hz, C_3H), 3.99 (m, 1H, C_5H), 4.25 (m, 2H, C_2H, C_6H), 4.41 (dd, 1H, J = 2.1, 11.7 Hz, C_6H), 4.99 (m, 2H, C_7'H_2), 5.82 (d, 1H, J = 15.6 Hz, C_2''H), 6.19 (m, 4H, C_3'H, C_5'H, C_4''H, C_5''H), 7.25 (m, 1H, C_3''H), HSCQ (125 MHz, CD_3OD) δ 18.3 (C_6''H_3), 65.0 (C_6H_2), 71.9 (C_4H), 73.3 (C_2H), 73.4 (C_5H), 73.6 (C_7'H_2), 76.3 (C_3H), 99.6, 102.8 (C_3'H, C_5'H), 119.7 (C_2''H). Additionally from 1D ^{13}C NMR (75 MHz, CD_3OD) δ 112.1 (C_1), 116.7 (C_1'), 131.0 (C_4''H), 140.0 (C_5''H), 145.4 (C_6'), 146.9 (C_3''H), 155.2, 161.4 (C_2', C_4'), 169.0 (C=O); HRMS: Calcd for $C_{19}H_{22}O_9Na$ $[M + Na]^+$ 417.1162, found 417.1132.

Palmitic acid mimic, 49

Compound **45** (50 mg, 0.075 mmol) was dissolved in dry acetonitrile (0.5 mL) and 1,3-propanediol (11 µL, 0.149 mmol) was added. This solution was added to a flask,

which contained $Sc(OTf)_3$ (6 mg, 0.011 mmol) and was put under an argon atmosphere. This reaction mixture was stirred at 50 °C for 3 h, after which it was concentrated. Compound **49** was obtained after preparative HPLC and lyophilization in 9% isolated yield (3.5 mg, 6.49 × 10^{-3} mmol). ^1H NMR (500 MHz, CD_3OD) δ 0.90 (t, 3H, *J* = 6.5 Hz, C_{16}''H_3), 1.29 (s, 24H, 12 × CH_2 4''-15''), 1.65 (m, 2H, C_3''H_2), 2.41 (m, 2H, C_2''H_2), 3.65 (m, 1H, C_4H), 3.75 (m, 2H, C_6H_2), 3.86 (m, 1H, C_5H), 4.29 (d, 1H, *J* = 10.0 Hz, C_2H), 5.04 (m, 2H, C_7'H_2), 5.29 (t, 1H, *J* = 10.0 Hz, C_3H), 6.20 (m, 2H, C_3'H, C_5'H); ^{13}C NMR (75 MHz, CD_3OD) δ 14.4 (C_{16}''H_3), 24.2 (C_{15}''H_2), 26.1 (C_3''H_2), 30.7 (10 × CH_2 4''-13''), 33.1 (C_{14}''H_2), 35.3 (C_2''H_2), 62.5 (C_6H_2), 69.7 (C_4H), 71.9 (C_2H), 73.8 (C_7'H_2), 75.8 (C_5H), 78.1 (C_3H), 99.9, 102.9 (C_3'H, C_5'H), 112.0 (C_1), 116.6 (C_1'), 145.5 (C_6'), 154.7, 161.5 (C_2', C_4'), 175.6 (C=O); HRMS: Calcd for $C_{29}H_{46}O_9Na$ $[M + Na]^+$ 561.3040, found 561.3054.

Linoleic acid mimic, 50

Compound **46** (79 mg, 0.11 mmol) was dissolved in dry MeOH (2.5 mL) and a small amount of Dowex 50 × 8 was added. This reaction mixture was stirred at 50 °C for 20 h after which it was filtered and concentrated. Compound **50** was obtained after preparative HPLC and lyophilization in 43% isolated yield (27 mg, 0.048 mmol). ^1H NMR (300 MHz, CD_3OD) δ 0.91 (t, 3H, *J* = 6.9 Hz, C_{18}''H_3), 1.35 (m, 14H, 7 × CH_2 4''-7'', 15''-17''), 1.66 (m, 2H, C_3''H_2), 2.06 (m, 4H, C_8''H_2, C_{14}''H_2), 2.42 (t, 2H, *J* = 7.2 Hz, C_2''H_2), 2.78 (t, 2H, *J* = 5.7 Hz, C_{11}''H_2), 3.65 (t, 1H, *J* = 9.6 Hz, C_4H), 3.75 (m, 2H, C_6H_2), 3.87 (m, 1H, C_5H), 4.29 (d, 1H, *J* = 9.9 Hz, C_2H), 5.02 (dd, 2H, *J* = 12.3, 20.7 Hz, C_7'H_2), 5.33 (m, 5H, C_9''H, C_{10}''H, C_{12}''H, C_{13}''H, C_3H), 6.20 (m, 2H, C_3'H, C_5'H); ^{13}C NMR (75 MHz, CD_3OD) δ 14.4 (C_{18}''H_3), 23.6 (C_{17}''H_2), 24.2 (C_5''H_2), 26.0 (C_3''H_2), 26.5 (C_{11}''H_2), 28.1 (C_8''H_2), 30.2, 30.4, 30.5, 30.7 (C_4''H_2, C_6''H_2,

$C_7''H_2$, $C_{14}'H_2$, $C_{15}''H_2$), 32.6 ($C_{16}''H_2$), 35.3 ($C_2''H_2$), 62.5 (C_6H_2), 69.7 (C_4H), 71.8 (C_2H), 73.8 ($C_7'H_2$), 75.8 (C_5H), 78.1 (C_3H), 99.9, 102.9 ($C_3'H$, $C_5'H$), 112.0 (C_1), 116.6 (C_1'), 129.0 ($C_{10}''H$, $C_{12}''H$), 130.9 ($C_9''H$, $C_{13}''H$), 145.5 (C_6'), 154.7, 161.5 (C_2', C_4'), 175.6 (C=O); HRMS: Calcd for $C_{31}H_{47}O_9$ $[M + H]^+$ 563.3220, found 563.3199; Calcd for $C_{31}H_{46}O_9Na$ $[M + Na]^+$ 585.3040, found 585.3024.

All trans-retinoic acid mimic, 51

Compound **47** (61 mg, 0.085 mmol) was dissolved in dry MeOH (2.5 mL) and a small amount of Dowex 50 × 8 was added. This reaction mixture was stirred at 50 °C for 20 h after which it was filtered and concentrated. Compound **51** was obtained after preparative HPLC and lyophilization in 7% isolated yield (3.6 mg, 0.0062 mmol). Due to degradation/aggregation of the compound during the measurements, no ^1H and ^{13}C NMR and MS spectra could be obtained.

Biological assay

Minimum inhibitory concentrations (MIC's) were determined by serial dilution in medium. The medium used in this assay was yeast extract peptone dextrose (YPD) containing 1% yeast extract, 2% peptone, 1% dextrose in distilled water. In Microtiter plates 1:1 dilution series of the appropriate antifungal agent were made and each one was well inoculated with a fresh culture of *Candida Albicans* (CBS 9975) obtained from the CBS-KNAW Fungal Biodiversity Centre (Utrecht, The Netherlands). The total volume per well was 200 μL. The plates were incubated overnight at 30 °C prior to the recording of the MIC's. The experiments were performed in duplicate.

1.0 mg/mL compound in DMSO

5 × diluted with medium → stock solution 0.2 mg/mL

**Starting concentrations (μg/mL)**	**Stock solution (μL)**	**Medium (μL)**
200	200	0
175	175	25
150	150	50
125	125	75

Microtiter plates:

1) 100 μL of medium was added to each of the wells.
2) 100 μL of the starting concentrations was added to the first column.
3) 100 μL of each concentration in column 1 is diluted across each row, except for the last column, this should contain no antifungal (final volume in each well is 100 μL).

Final concentrations in µg/mL

	1	2	3	4	5	6	7	8	9	10	11	12
**A**	100	50	25	12.5	6.25	3.125	1.563	0.781	0.391	0.195	0.098	0
**B**	87.5	43.75	21.88	10.94	5.469	2.734	1.367	0.684	0.342	0.171	0.085	0
**C**	75	37.5	18.75	9.375	4.688	2.344	1.172	0.586	0.293	0.146	0.073	0
**D**	62.5	31.25	15.63	7.813	3.906	1.953	0.978	0.488	0.244	0.122	0.061	0
**E**	100	50	25	12.5	6.25	3.125	1.563	0.781	0.391	0.195	0.098	0
**F**	87.5	43.75	21.88	10.94	5.469	2.734	1.367	0.684	0.342	0.171	0.085	0
**G**	75	37.5	18.75	9.375	4.688	2.344	1.172	0.586	0.293	0.146	0.073	0
**H**	62.5	31.25	15.63	7.813	3.906	1.953	0.978	0.488	0.244	0.122	0.061	0

4) Plates are then ready for *Candida Albicans* to be added, 100 µL per well (final volume in each well is then 200 µL).

Plates were placed in the oven at 30 °C for 20 h, before determination of MIC’s. MIC’s are determined by locating the last well before fungal growth occurs.

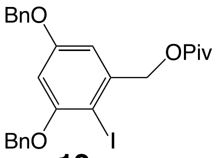
BnO
OPiv
BnO
I

12

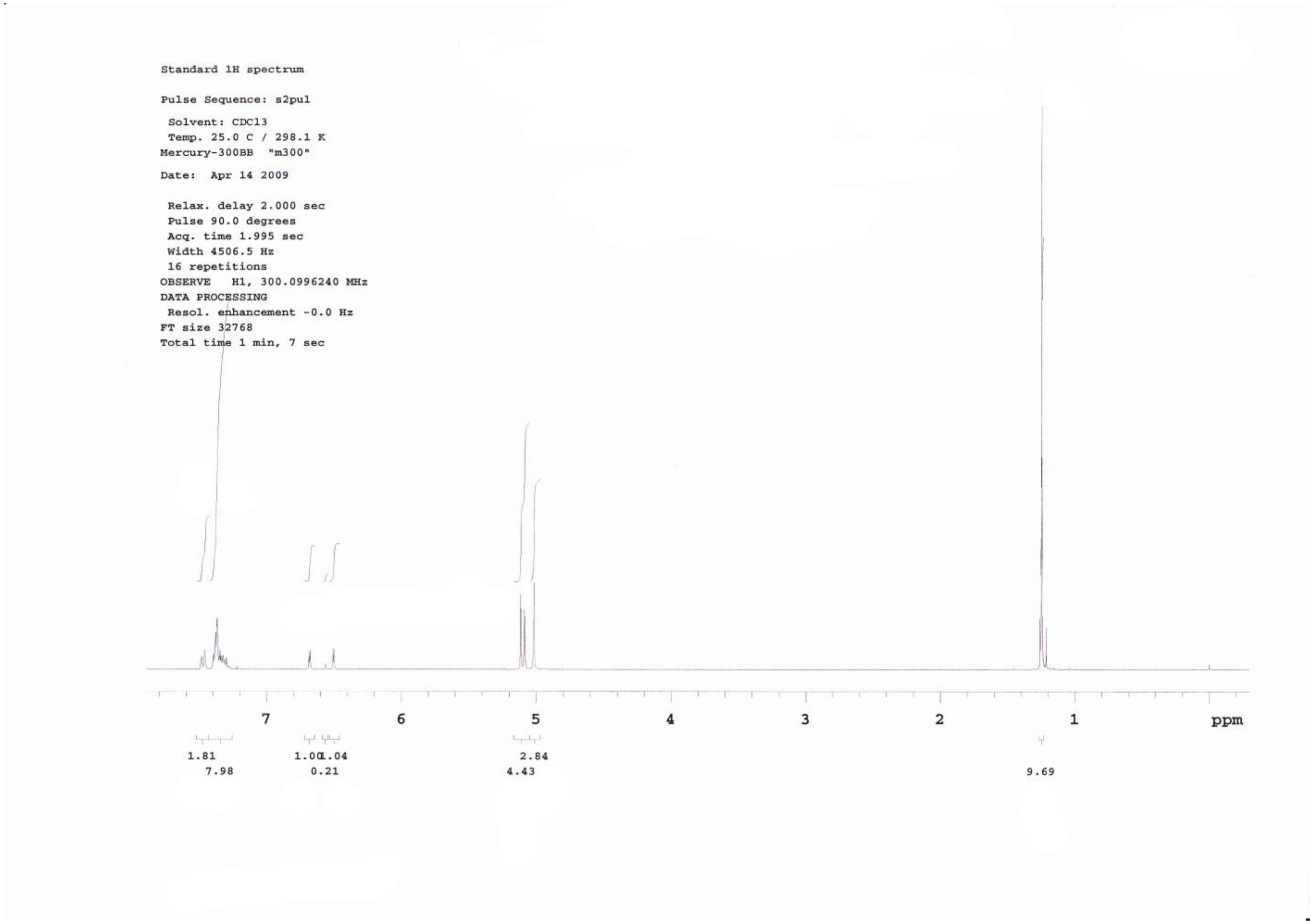
Standard 1H spectrum
Pulse Sequence: s2pul
Solvent: CDCl3
Temp. 25.0 C / 298.1 K
Mercury-300BB "m300"
Date: Apr 14 2009
Relax. delay 2.000 sec
Pulse 90.0 degrees
Acq. time 1.995 sec
Width 4506.5 Hz
16 repetitions
OBSERVE H1, 300.0996240 MHz
DATA PROCESSING
Resol. enhancement -0.0 Hz
FT size 32768
Total time 1 min, 7 sec
7
6
5
4
3
2
1
ppm
1.81
7.98
1.00
1.04
0.21
2.84
4.43
9.69

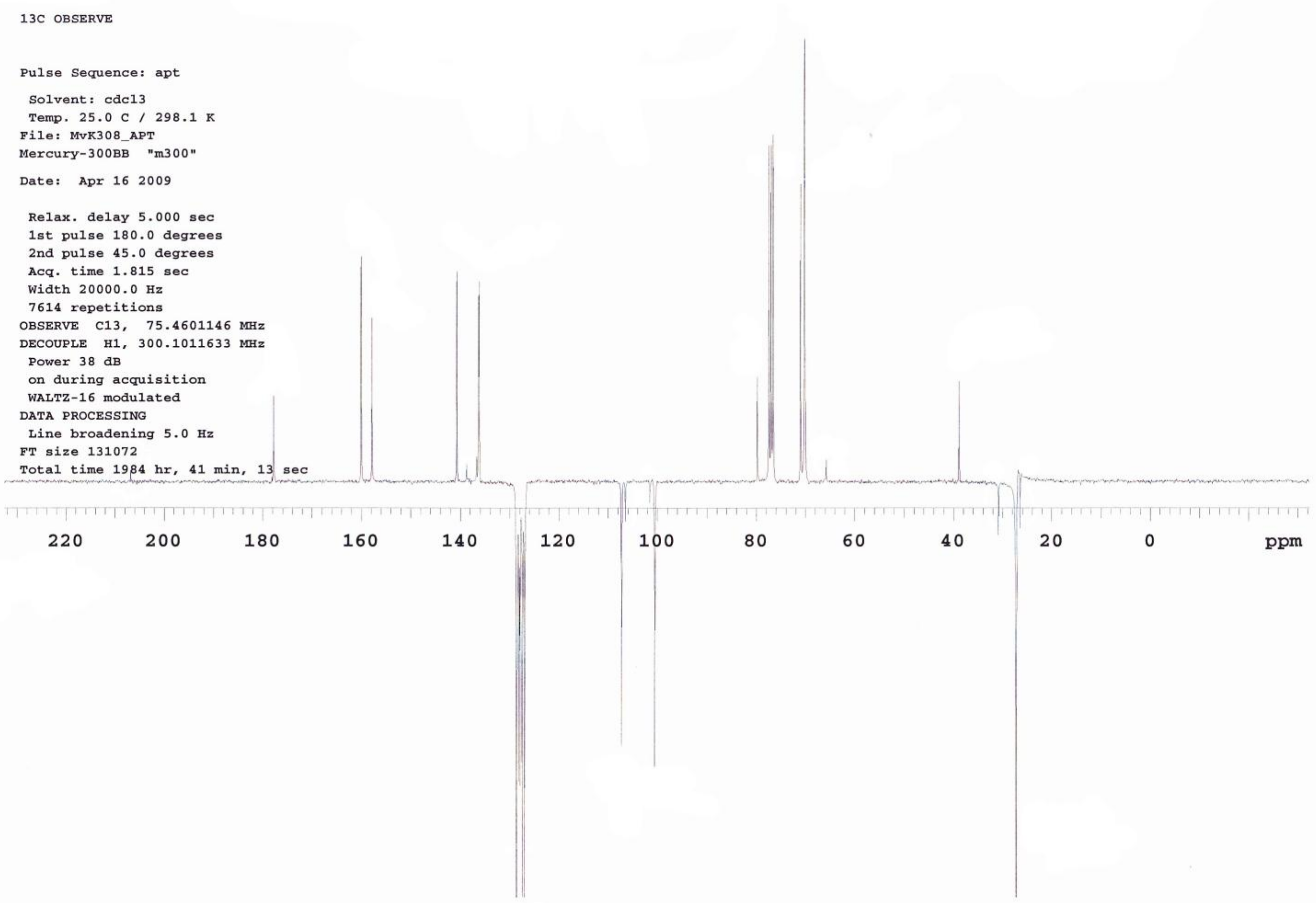
13C OBSERVE
Pulse Sequence: apt
Solvent: cdcl3
Temp. 25.0 C / 298.1 K
File: MvK308_APT
Mercury-300BB "m300"
Date: Apr 16 2009
Relax. delay 5.000 sec
1st pulse 180.0 degrees
2nd pulse 45.0 degrees
Acq. time 1.815 sec
Width 20000.0 Hz
7614 repetitions
OBSERVE C13, 75.4601146 MHz
DECOUPLE H1, 300.1011633 MHz
Power 38 dB
on during acquisition
WALTZ-16 modulated
DATA PROCESSING
Line broadening 5.0 Hz
FT size 131072
Total time 1984 hr, 41 min, 13 sec
220 200 180 160 140 120 100 80 60 40 20 0 ppm

compound **12**

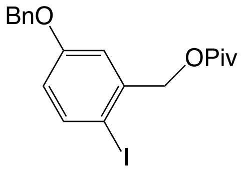
BnO
OPiv
I

13

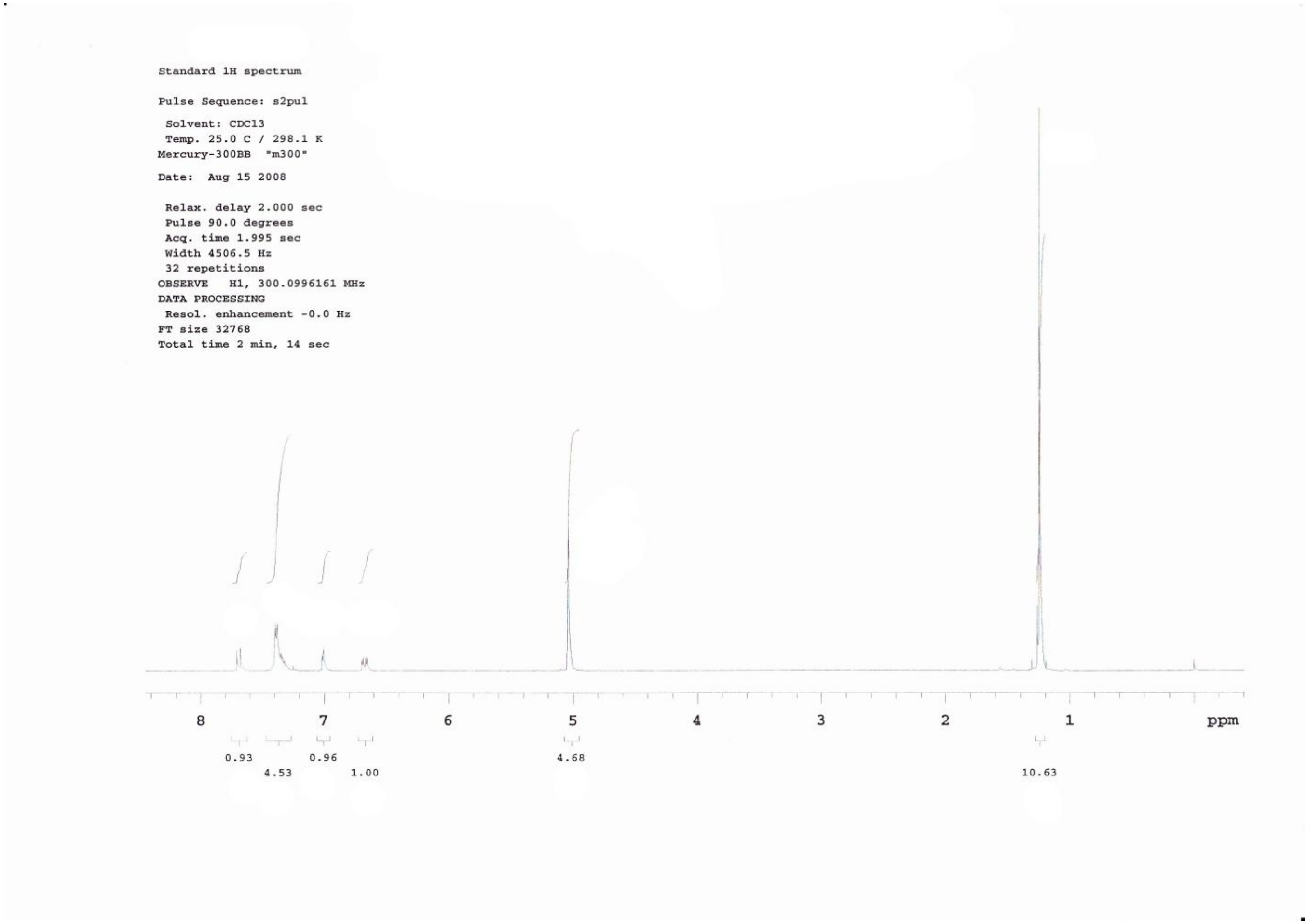
Standard 1H spectrum
Pulse Sequence: s2pul
Solvent: CDCl3
Temp. 25.0 C / 298.1 K
Mercury-300BB "m300"
Date: Aug 15 2008
Relax. delay 2.000 sec
Pulse 90.0 degrees
Acq. time 1.995 sec
Width 4506.5 Hz
32 repetitions
OBSERVE H1, 300.0996161 MHz
DATA PROCESSING
Resol. enhancement -0.0 Hz
FT size 32768
Total time 2 min, 14 sec
8
7
6
5
4
3
2
1
ppm
0.93
4.53
0.96
1.00
4.68
10.63

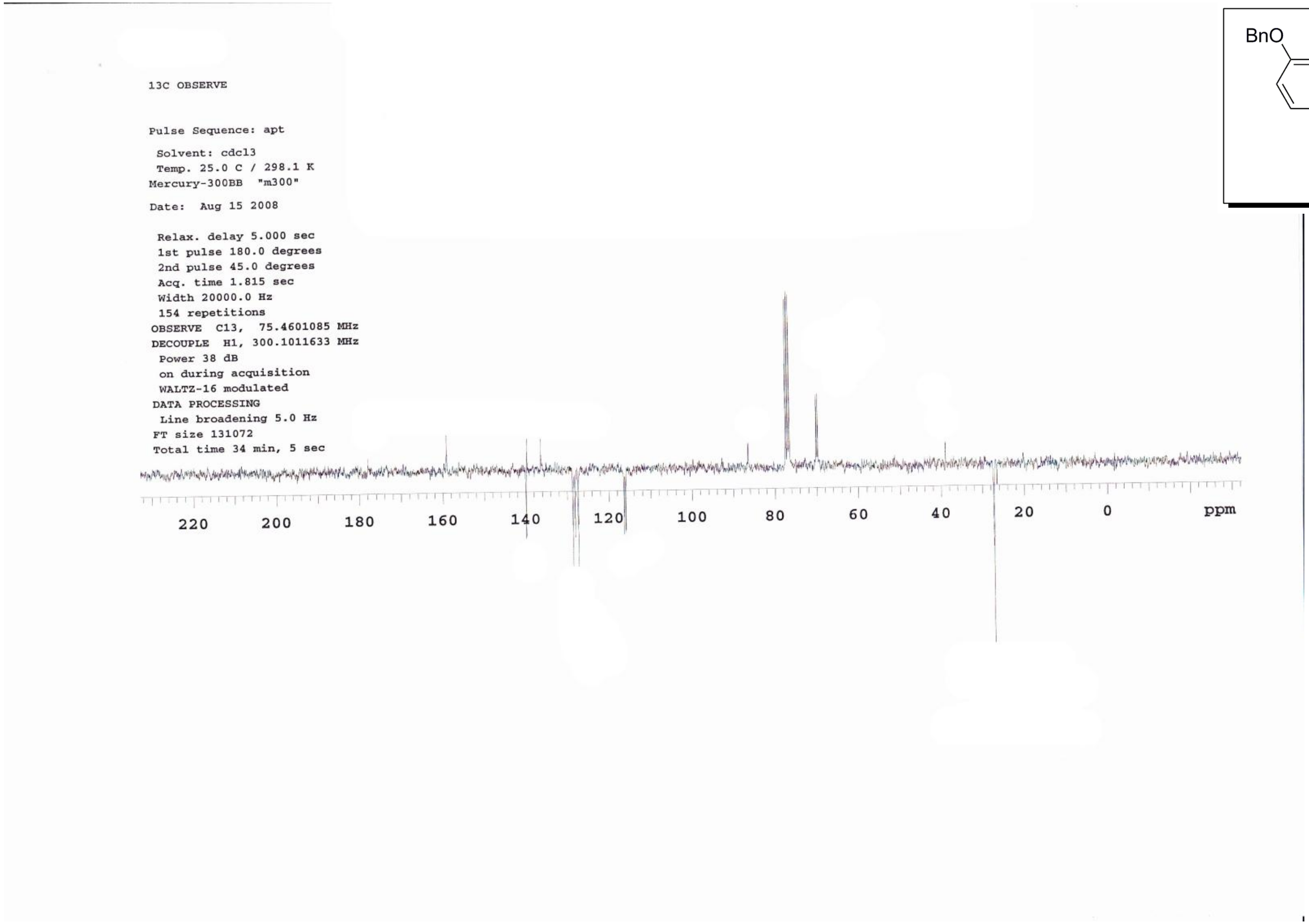

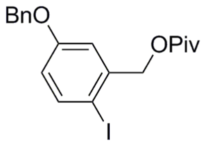

14

compound **13**

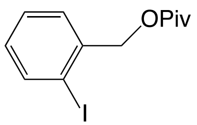
OPiv
I

14

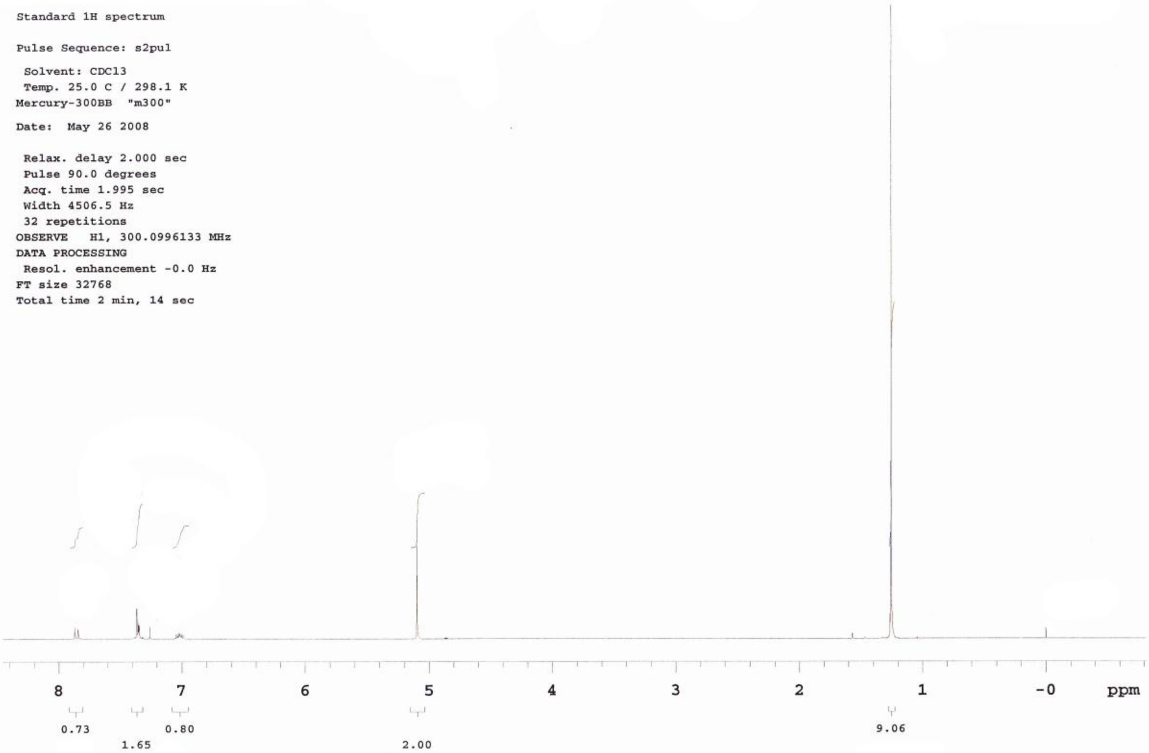
Standard 1H spectrum
Pulse Sequence: s2pul
Solvent: CDCl3
Temp. 25.0 C / 298.1 K
Mercury-300BB "m300"
Date: May 26 2008
Relax. delay 2.000 sec
Pulse 90.0 degrees
Acq. time 1.995 sec
Width 4506.5 Hz
32 repetitions
OBSERVE H1, 300.0996133 MHz
DATA PROCESSING
Resol. enhancement -0.0 Hz
FT size 32768
Total time 2 min, 14 sec
8
7
6
5
4
3
2
1
-0
ppm
0.73
1.65
0.80
2.00
9.06

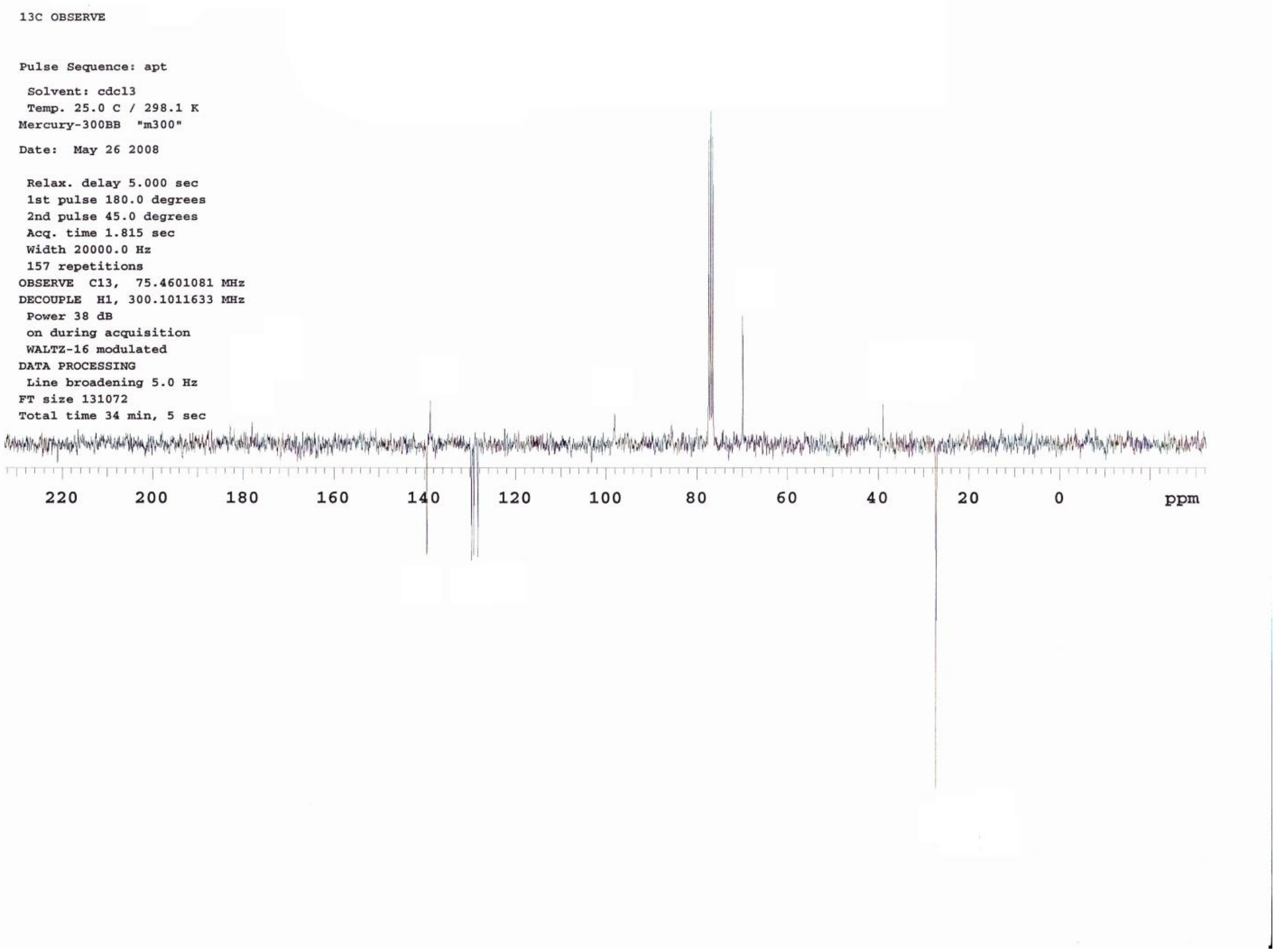
13C OBSERVE
Pulse Sequence: apt
Solvent: cdcl3
Temp. 25.0 C / 298.1 K
Mercury-300BB "m300"
Date: May 26 2008
Relax. delay 5.000 sec
1st pulse 180.0 degrees
2nd pulse 45.0 degrees
Acq. time 1.815 sec
Width 20000.0 Hz
157 repetitions
OBSERVE C13, 75.4601081 MHz
DECOUPLE H1, 300.1011633 MHz
Power 38 dB
on during acquisition
WALTZ-16 modulated
DATA PROCESSING
Line broadening 5.0 Hz
FT size 131072
Total time 34 min, 5 sec
220 200 180 160 140 120 100 80 60 40 20 0 ppm

compound **14**

Standard 1H spectrum

Pulse Sequence: s2pul

Solvent: CDCl3
Temp. 25.0 C / 298.1 K
Mercury-300BB "m300"

Date: Mar 17 2008

Relax. delay 2.000 sec
Pulse 90.0 degrees
Acq. time 1.995 sec
Width 4506.5 Hz
32 repetitions
OBSERVE H1, 300.0996125 MHz
DATA PROCESSING
Resol. enhancement -0.0 Hz
FT size 32768
Total time 2 min, 14 sec

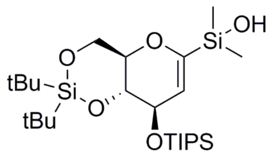

22

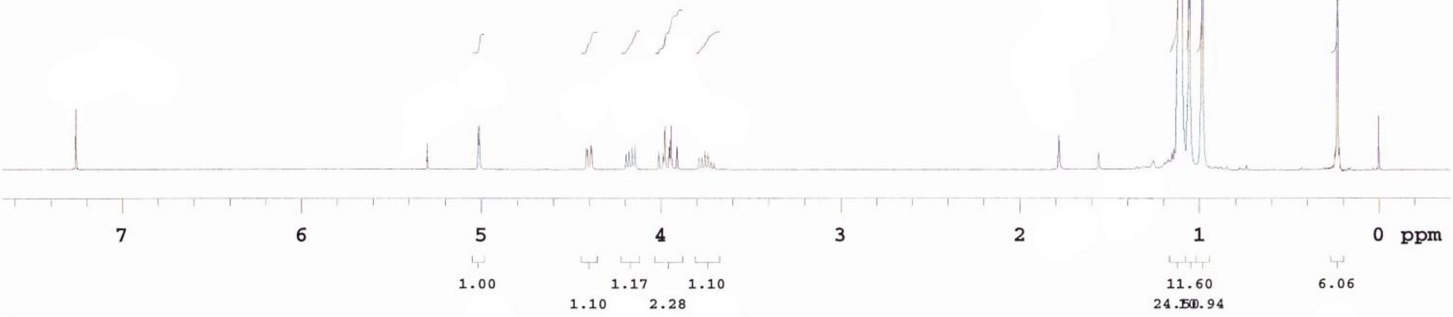

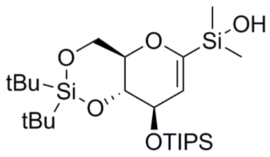

22

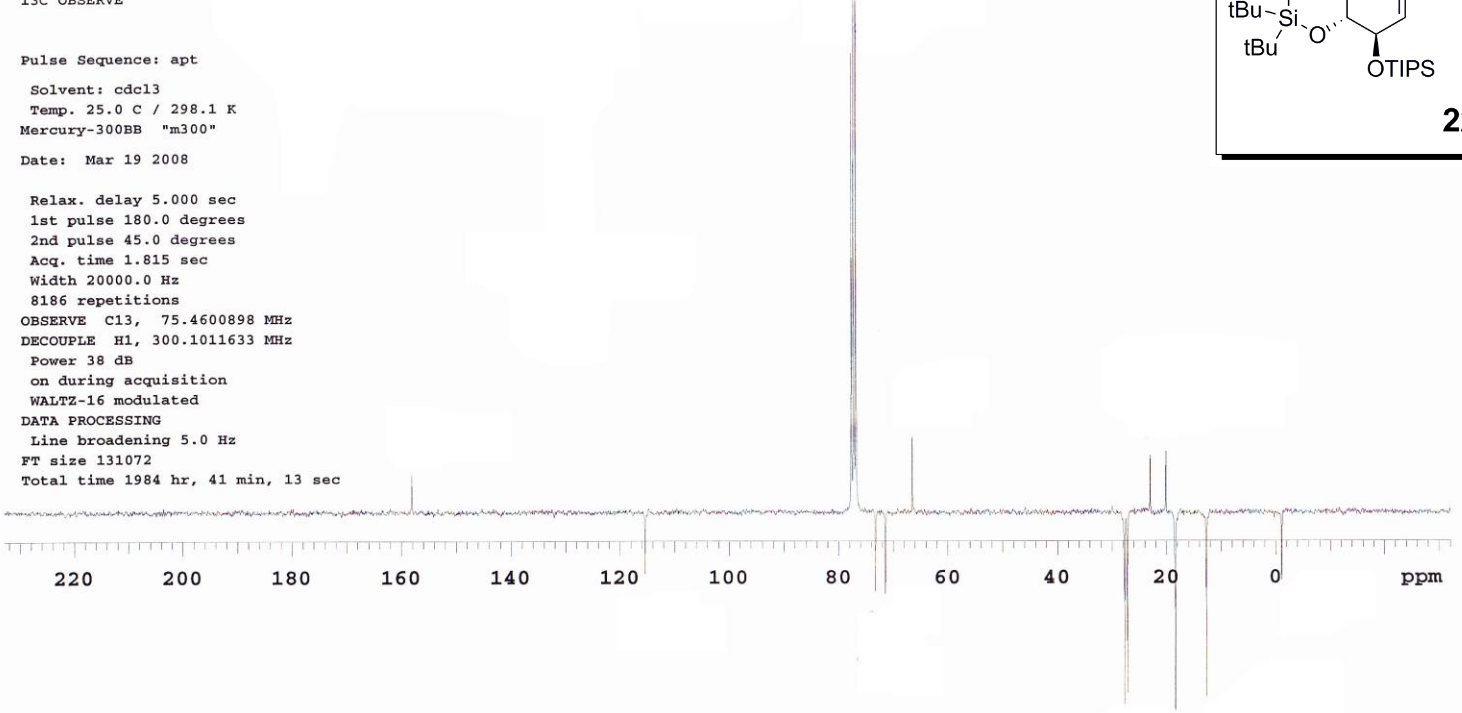
13C OBSERVE
Pulse Sequence: apt
Solvent: cdcl3
Temp. 25.0 C / 298.1 K
Mercury-300BB "m300"
Date: Mar 19 2008
Relax. delay 5.000 sec
1st pulse 180.0 degrees
2nd pulse 45.0 degrees
Acq. time 1.815 sec
Width 20000.0 Hz
8186 repetitions
OBSERVE C13, 75.4600898 MHz
DECOUPLE H1, 300.1011633 MHz
Power 38 dB
on during acquisition
WALTZ-16 modulated
DATA PROCESSING
Line broadening 5.0 Hz
FT size 131072
Total time 1984 hr, 41 min, 13 sec
220
200
180
160
140
120
100
80
60
40
20
0
ppm

Standard 1H spectrum

Pulse Sequence: s2pul

Solvent: CDCl3
Temp. 25.0 C / 298.1 K
Mercury-300BB "m300"

Date: Apr 18 2008

Relax. delay 2.000 sec
Pulse 90.0 degrees
Acq. time 1.995 sec
Width 4506.5 Hz
32 repetitions
OBSERVE H1, 300.0996232 MHz
DATA PROCESSING
Resol. enhancement -0.0 Hz
FT size 32768
Total time 2 min, 14 sec

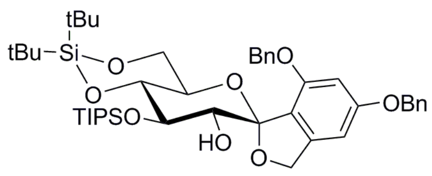

32

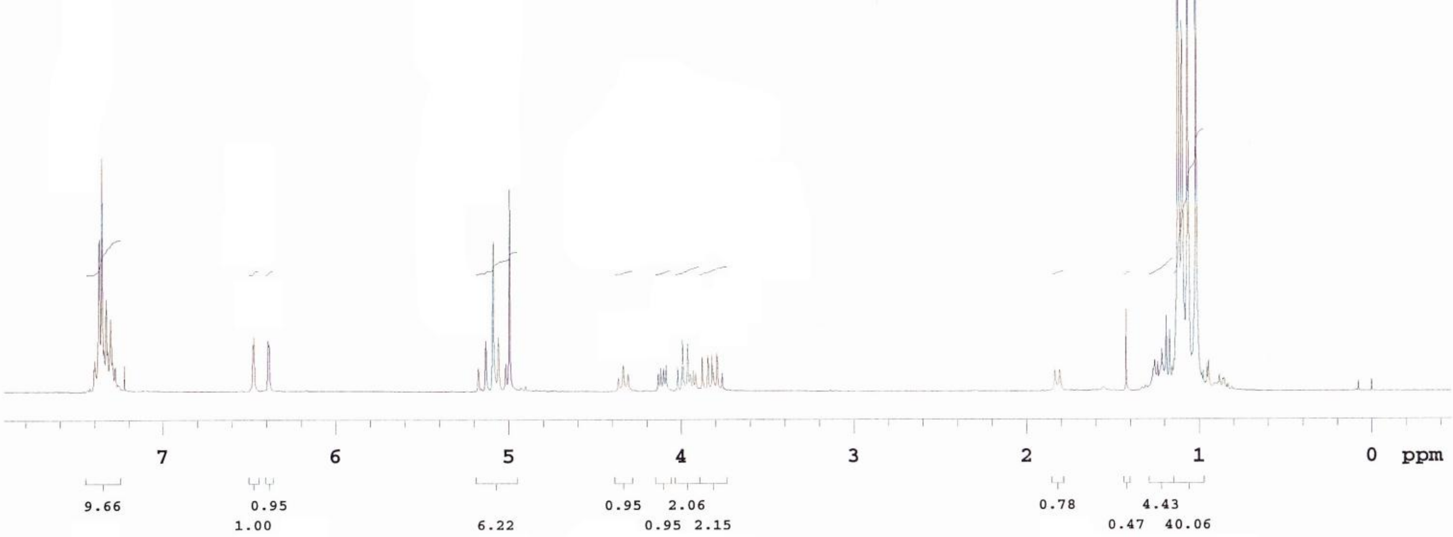

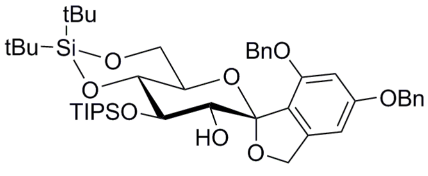
tBu
tBu–Si
O
O
TIPSO
HO
O
BnO
OBn
O

32

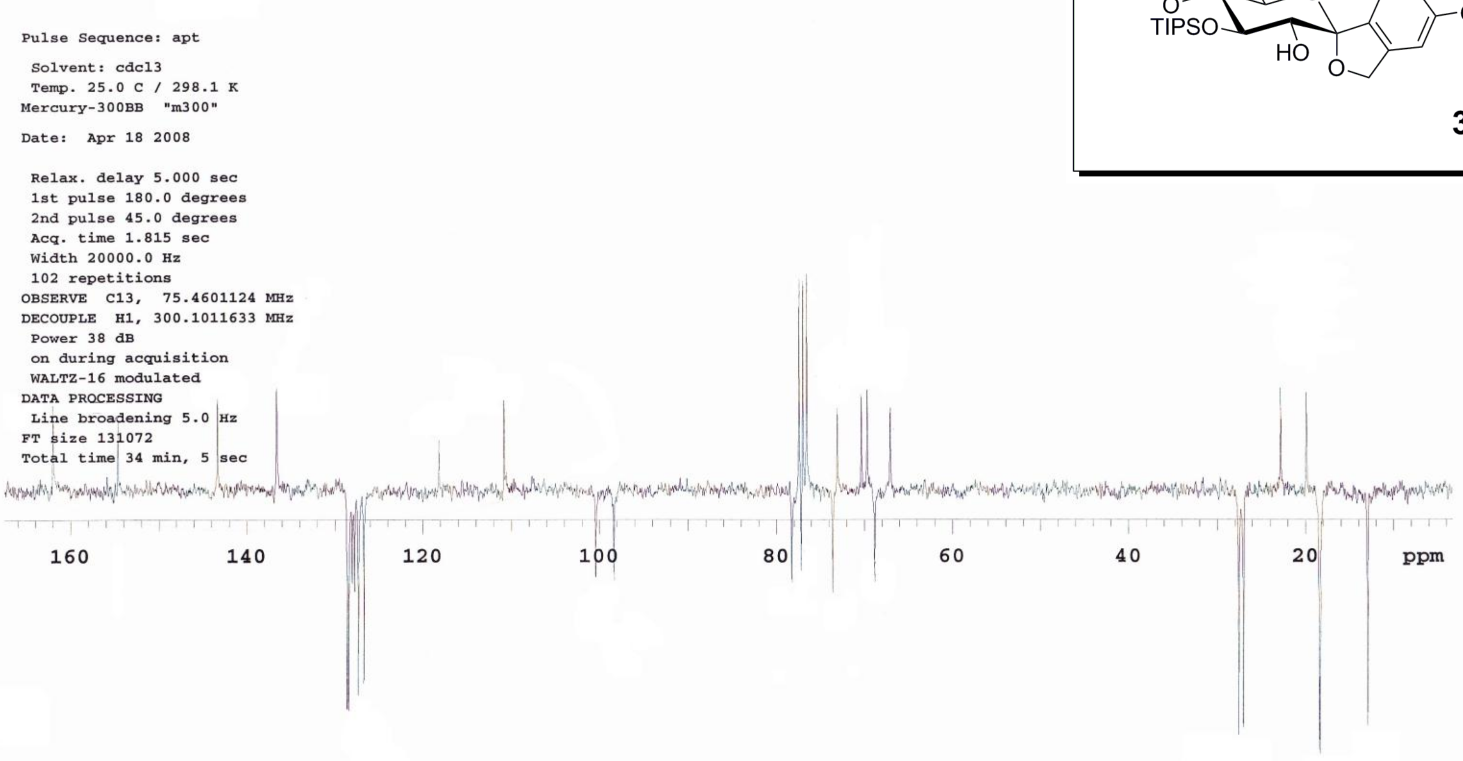
13C OBSERVE
Pulse Sequence: apt
Solvent: cdcl3
Temp. 25.0 C / 298.1 K
Mercury-300BB "m300"
Date: Apr 18 2008
Relax. delay 5.000 sec
1st pulse 180.0 degrees
2nd pulse 45.0 degrees
Acq. time 1.815 sec
Width 20000.0 Hz
102 repetitions
OBSERVE C13, 75.4601124 MHz
DECOUPLE H1, 300.1011633 MHz
Power 38 dB
on during acquisition
WALTZ-16 modulated
DATA PROCESSING
Line broadening 5.0 Hz
FT size 131072
Total time 34 min, 5 sec
160
140
120
100
80
60
40
20
ppm

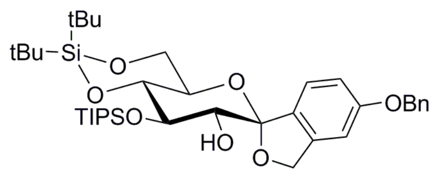

33

Standard 1H spectrum

Pulse Sequence: s2pul

Solvent: CDCl3
Temp. 25.0 C / 298.1 K
Mercury-300BB "m300"

Date: Aug 25 2008

Relax. delay 2.000 sec
Pulse 90.0 degrees
Acq. time 1.995 sec
Width 4506.5 Hz
64 repetitions
OBSERVE H1, 300.0996194 MHz
DATA PROCESSING
Resol. enhancement -0.0 Hz
FT size 32768
Total time 4 min, 28 sec

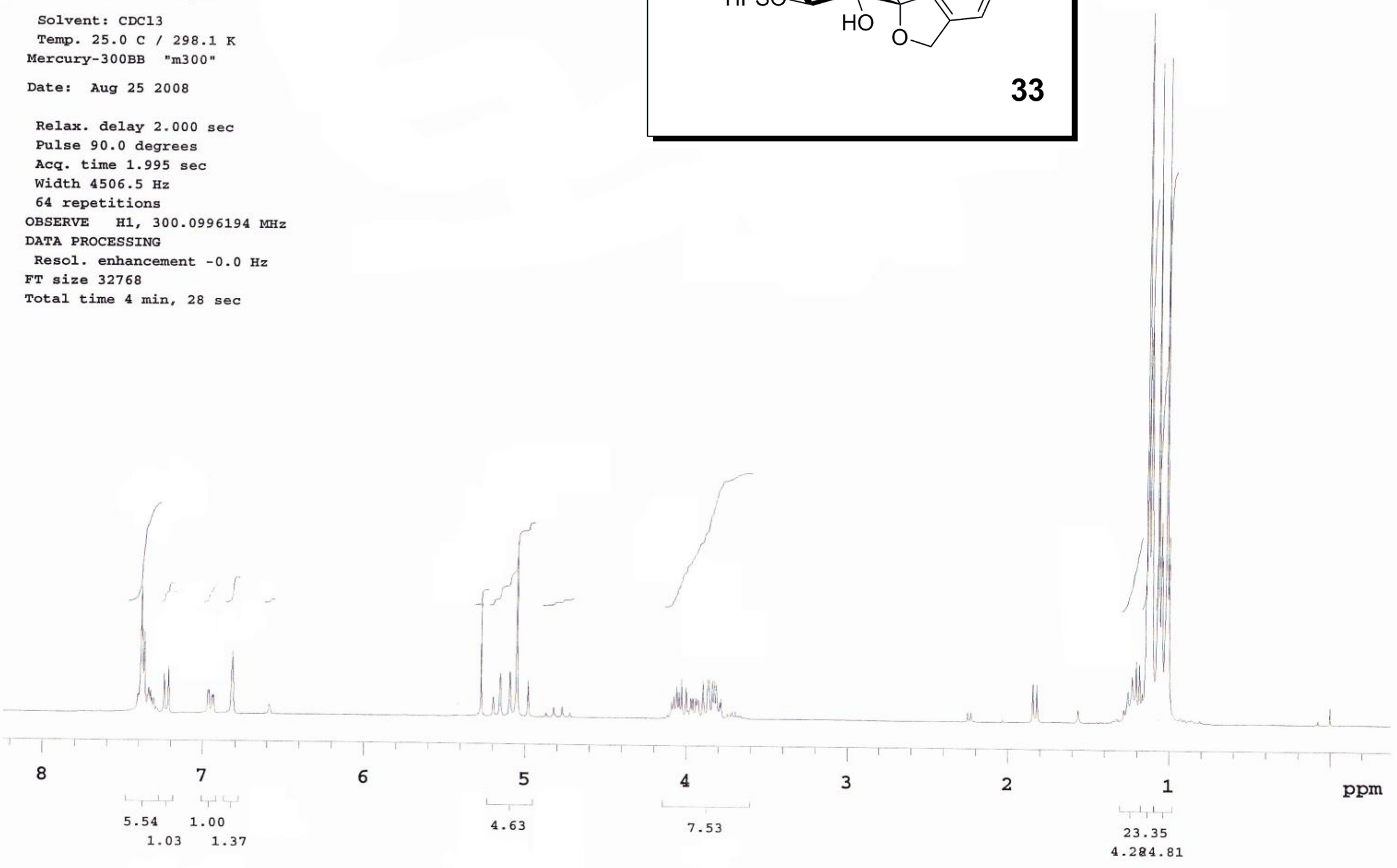

13C OBSERVE

Pulse Sequence: apt

Solvent: cdcl3
Temp. 25.0 C / 298.1 K
Mercury-300BB "m300"

Date: Aug 25 2008

Relax. delay 5.000 sec
1st pulse 180.0 degrees
2nd pulse 45.0 degrees
Acq. time 1.815 sec
Width 20000.0 Hz
989863412 repetitions
OBSERVE C13, 75.4601103 MHz
DECOUPLE H1, 300.1011633 MHz
Power 38 dB
on during acquisition
WALTZ-16 modulated
DATA PROCESSING
Line broadening 5.0 Hz
FT size 131072
Total time 1984686 hr, 56 min, 7 sec

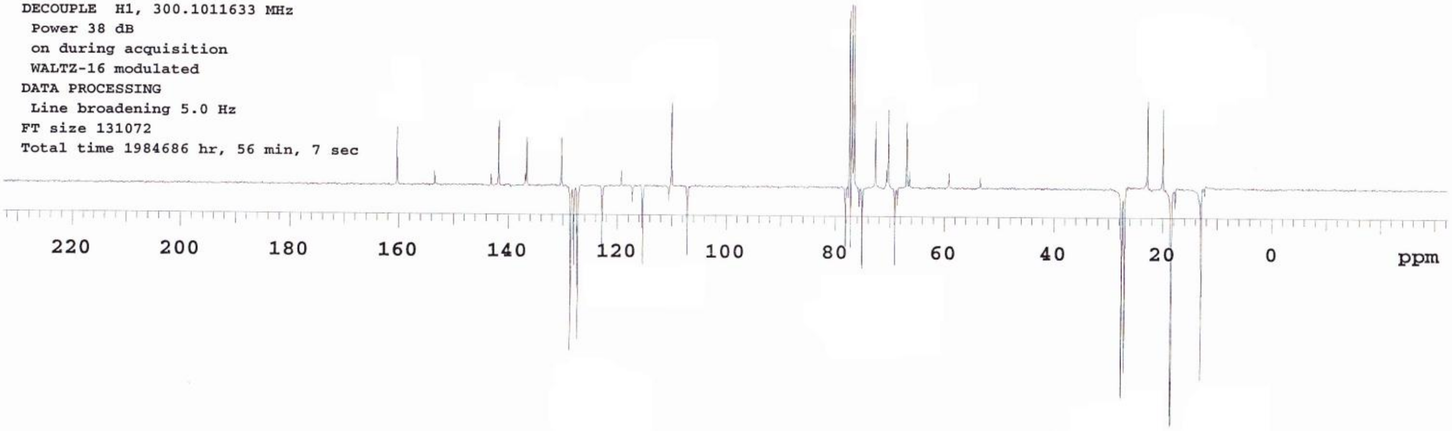

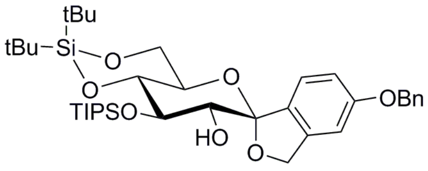

33

Standard 1H spectrum

Pulse Sequence: s2pul

Solvent: CDCl3
Temp. 25.0 C / 298.1 K
Mercury-300BB "m300"

Date: Aug 1 2008

Relax. delay 2.000 sec
Pulse 90.0 degrees
Acq. time 1.995 sec
Width 4506.5 Hz
32 repetitions
OBSERVE H1, 300.0996177 MHz
DATA PROCESSING
Resol. enhancement -0.0 Hz
FT size 32768
Total time 2 min, 14 sec

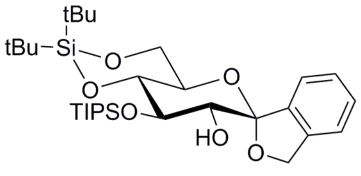

34

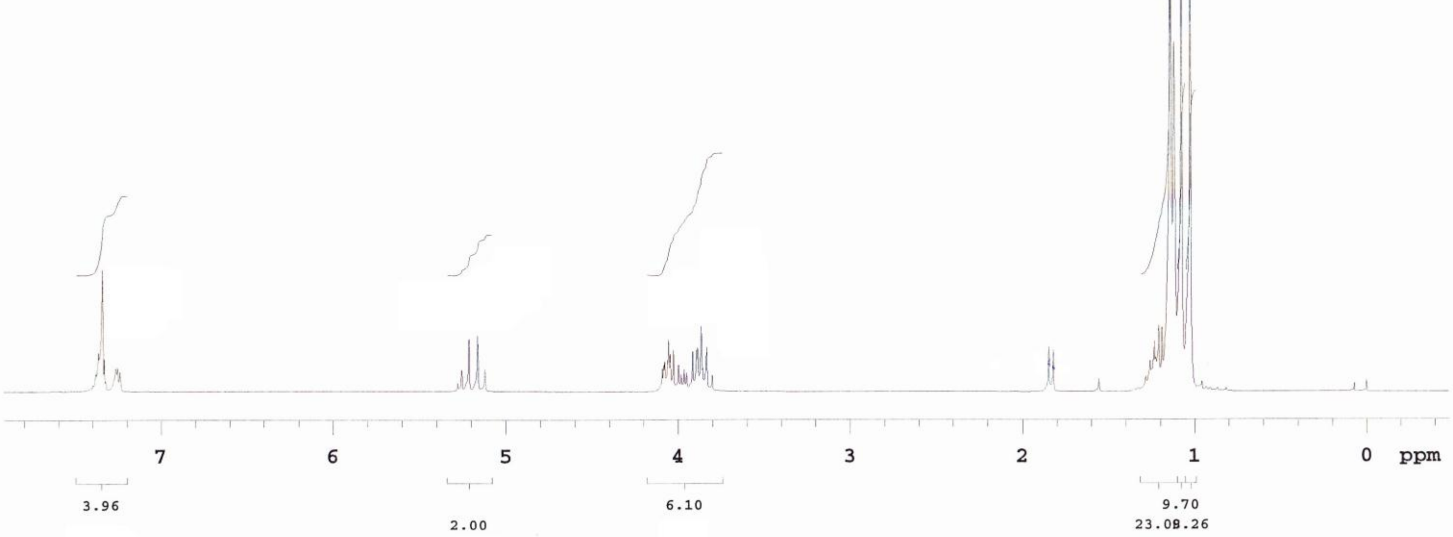

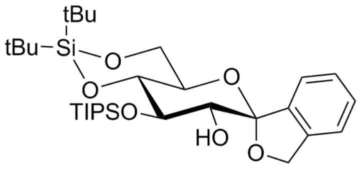
tBu
tBu
Si
O
O
O
TIPSO
HO
O
O

34

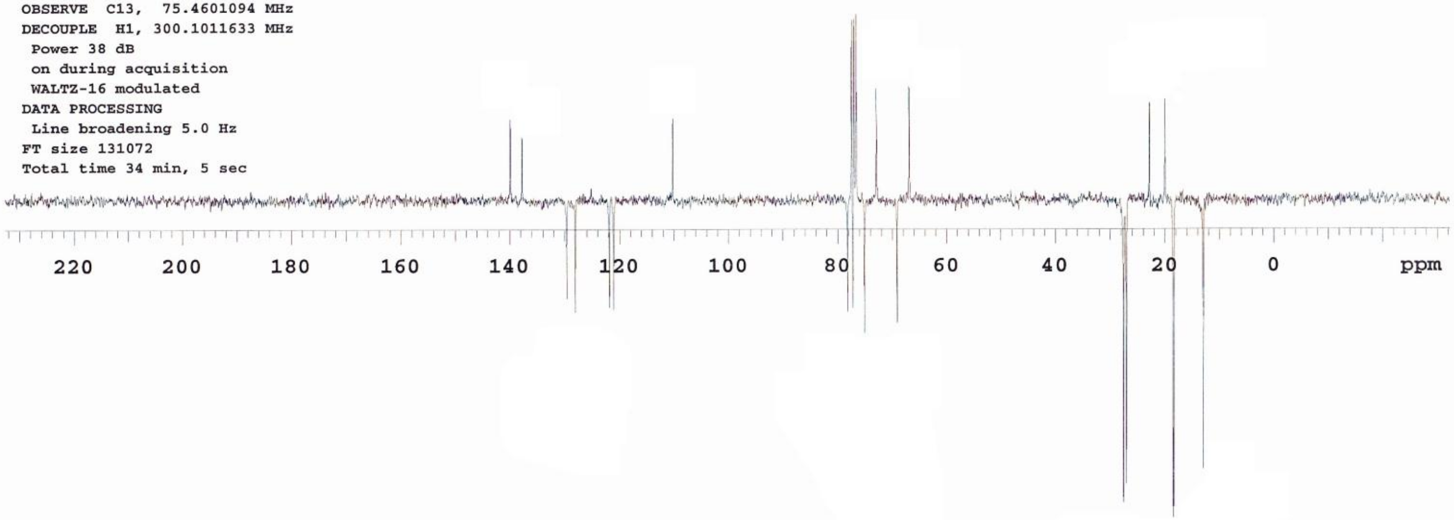
13C OBSERVE
Pulse Sequence: apt
Solvent: cdcl3
Temp. 25.0 C / 298.1 K
Mercury-300BB "m300"
Date: Aug 1 2008
Relax. delay 5.000 sec
1st pulse 180.0 degrees
2nd pulse 45.0 degrees
Acq. time 1.815 sec
Width 20000.0 Hz
193 repetitions
OBSERVE C13, 75.4601094 MHz
DECOUPLE H1, 300.1011633 MHz
Power 38 dB
on during acquisition
WALTZ-16 modulated
DATA PROCESSING
Line broadening 5.0 Hz
FT size 131072
Total time 34 min, 5 sec
220
200
180
160
140
120
100
80
60
40
20
0
ppm

Standard 1H spectrum

Pulse Sequence: s2pul

Solvent: CDCl3
Temp. 25.0 C / 298.1 K
Mercury-300BB "m300"

Date: May 28 2010

Relax. delay 2.000 sec
Pulse 90.0 degrees
Acq. time 1.995 sec
Width 4506.5 Hz
32 repetitions
OBSERVE H1, 300.0996125 MHz
DATA PROCESSING
Resol. enhancement -0.0 Hz
FT size 32768
Total time 2 min, 14 sec

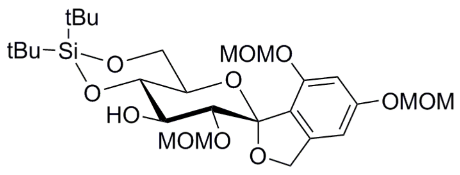

39

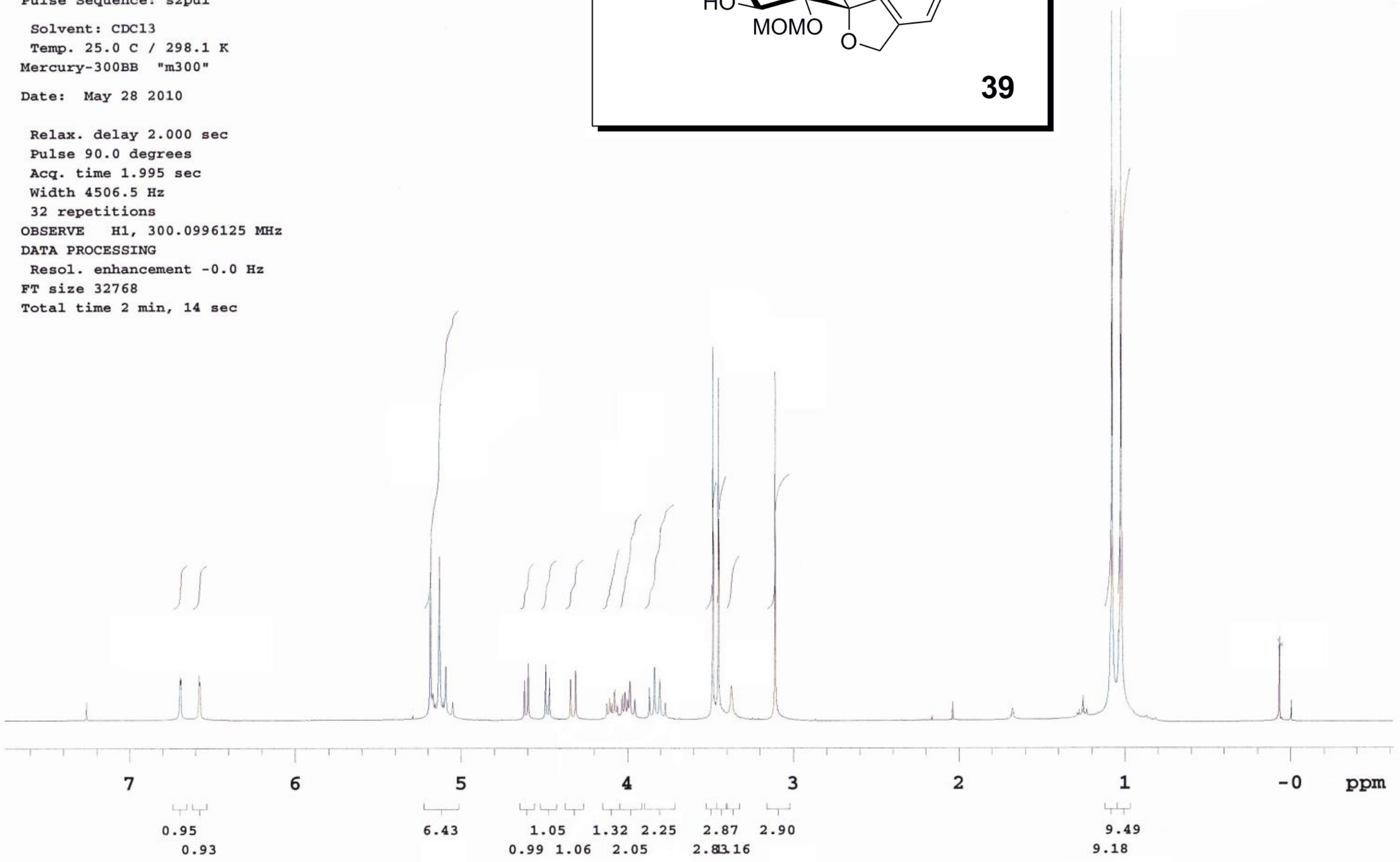

13C OBSERVE

Pulse Sequence: apt

Solvent: cdcl3
Temp. 25.0 C / 298.1 K
Mercury-300BB "m300"

Date: May 28 2010

Relax. delay 5.000 sec
1st pulse 180.0 degrees
2nd pulse 45.0 degrees
Acq. time 1.815 sec
Width 20000.0 Hz
389 repetitions
OBSERVE C13, 75.4601091 MHz
DECOUPLE H1, 300.1011633 MHz
Power 38 dB
on during acquisition
WALTZ-16 modulated
DATA PROCESSING
Line broadening 5.0 Hz
FT size 131072
Total time 221 hr, 54 min, 22 sec

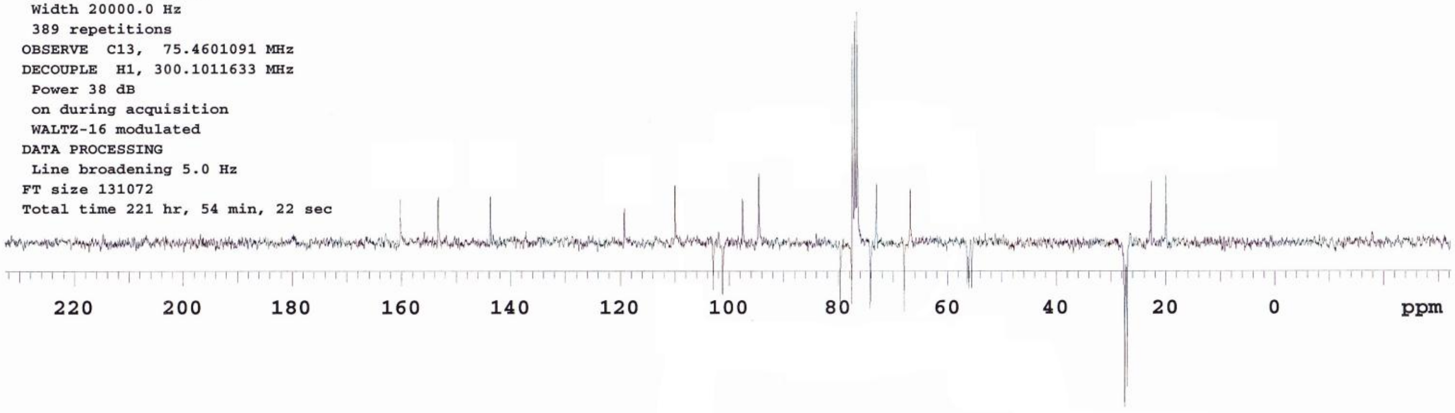

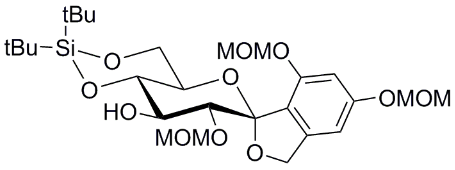

39

Standard 1H spectrum

Pulse Sequence: s2pul

Solvent: CDCl3
Temp. 25.0 C / 298.1 K
Mercury-300BB "m300"

Date: Aug 16 2010

Relax. delay 2.000 sec
Pulse 90.0 degrees
Acq. time 1.995 sec
Width 4506.5 Hz
64 repetitions
OBSERVE H1, 300.0996067 MHz
DATA PROCESSING
Resol. enhancement -0.0 Hz
FT size 32768
Total time 4 min, 28 sec

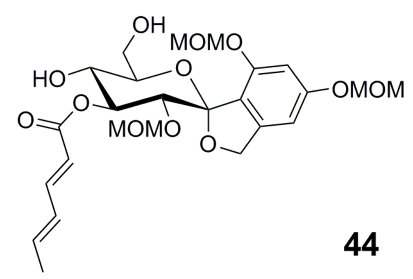

44

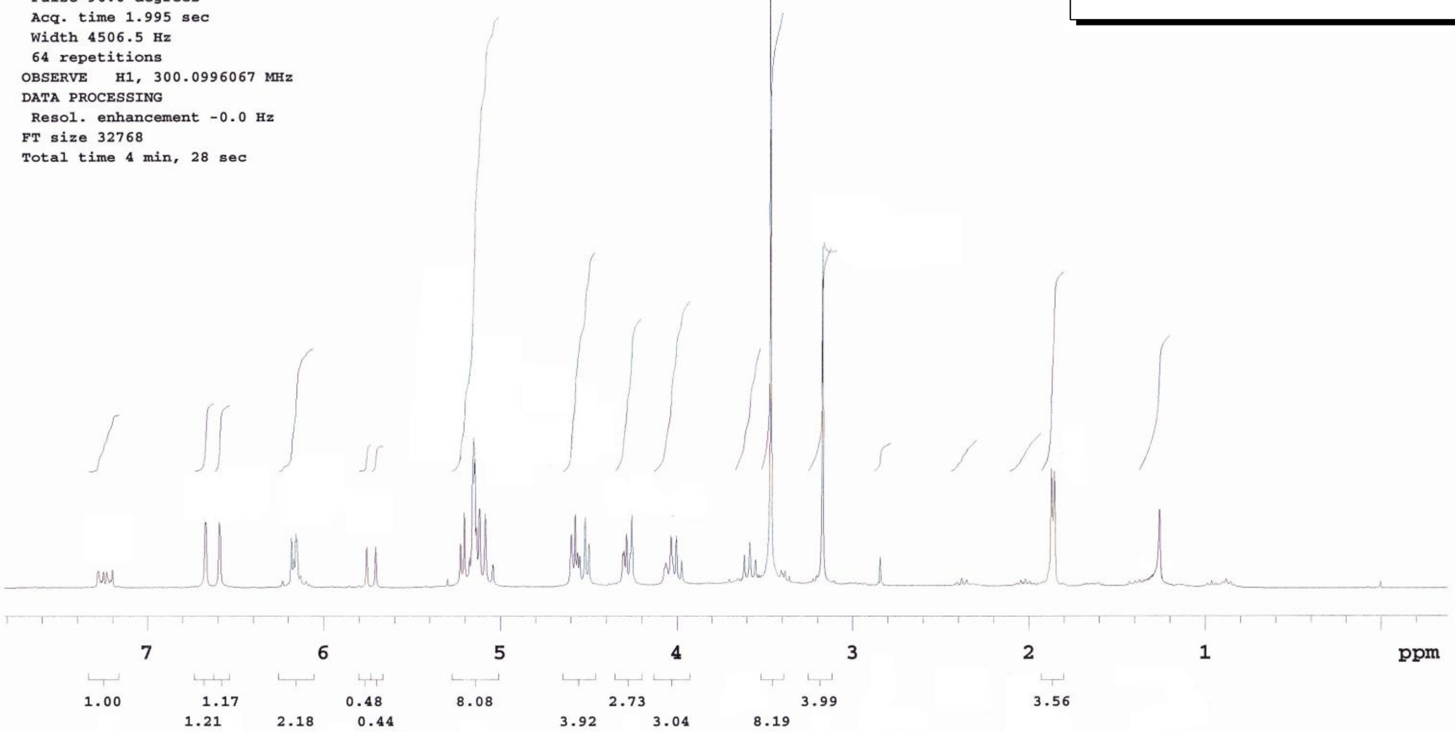

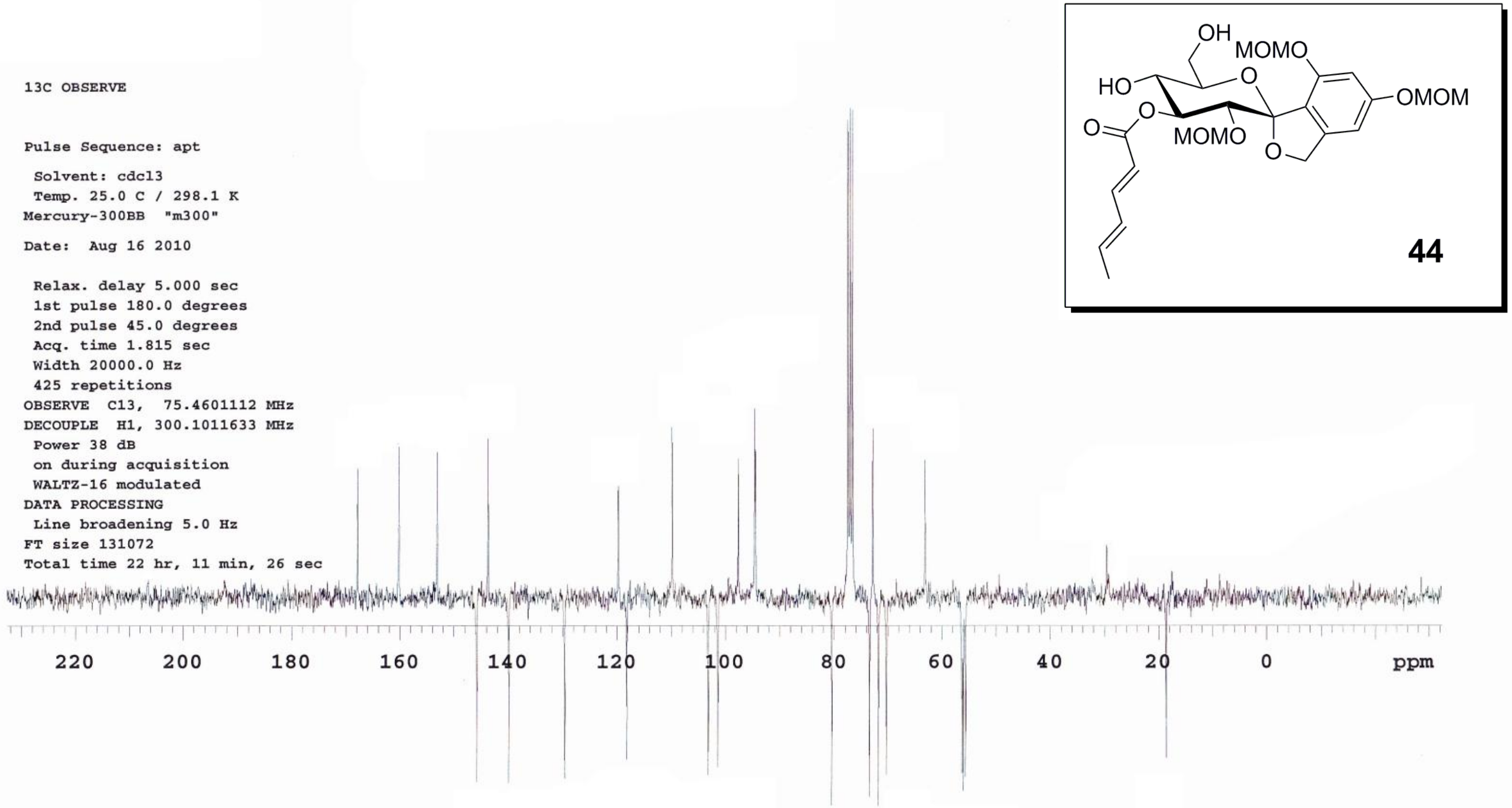

13C OBSERVE
Pulse Sequence: apt
Solvent: cdcl3
Temp. 25.0 C / 298.1 K
Mercury-300BB "m300"
Date: Aug 16 2010
Relax. delay 5.000 sec
1st pulse 180.0 degrees
2nd pulse 45.0 degrees
Acq. time 1.815 sec
Width 20000.0 Hz
425 repetitions
OBSERVE C13, 75.4601112 MHz
DECOUPLE H1, 300.1011633 MHz
Power 38 dB
on during acquisition
WALTZ-16 modulated
DATA PROCESSING
Line broadening 5.0 Hz
FT size 131072
Total time 22 hr, 11 min, 26 sec
220
200
180
160
140
120
100
80
60
40
20
0
ppm
OH
MOMO
HO
O
OMOM
O
O
MOMO
O

44

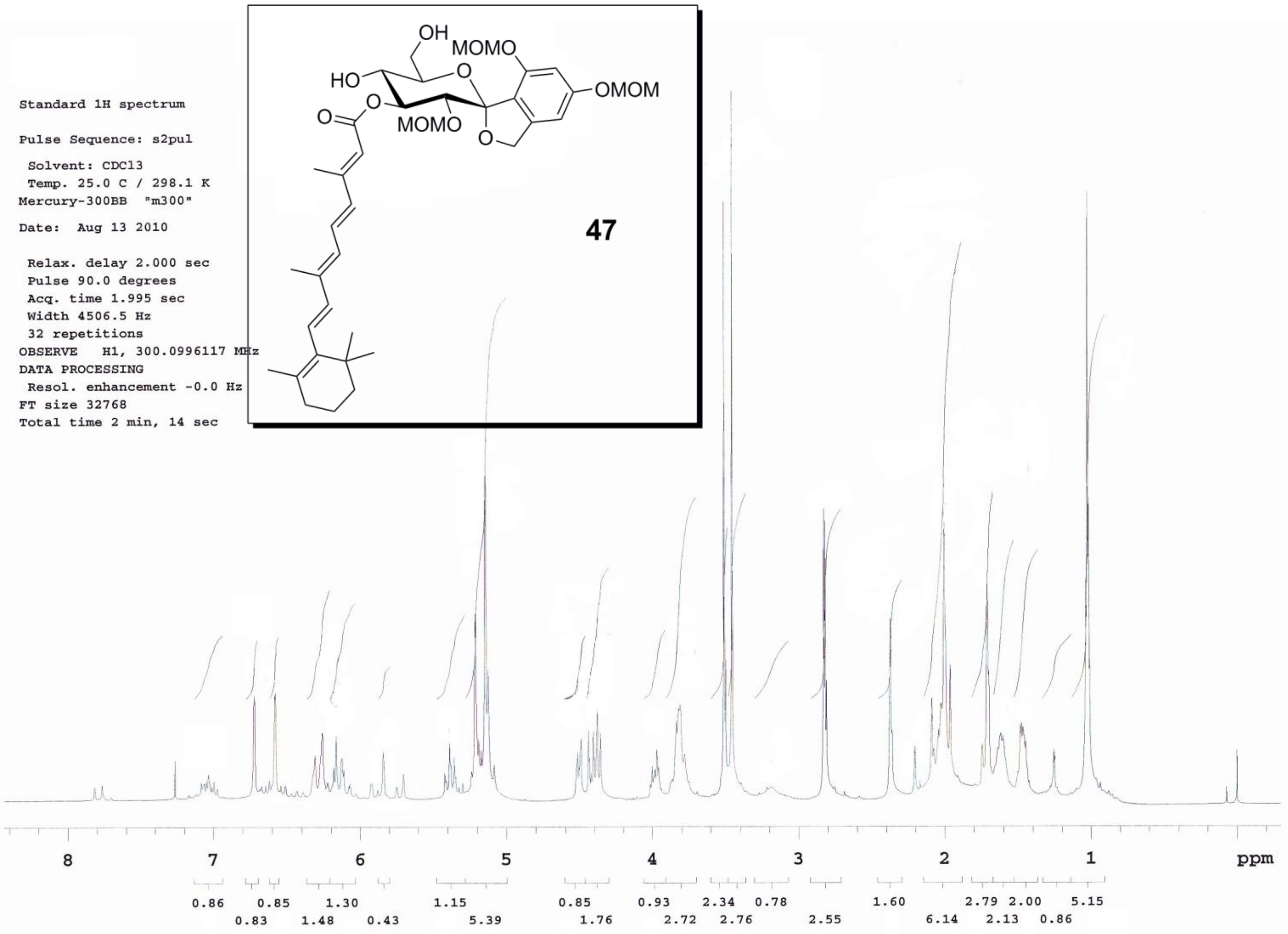
Standard 1H spectrum
Pulse Sequence: s2pul
Solvent: CDCl3
Temp. 25.0 C / 298.1 K
Mercury-300BB "m300"
Date: Aug 13 2010
Relax. delay 2.000 sec
Pulse 90.0 degrees
Acq. time 1.995 sec
Width 4506.5 Hz
32 repetitions
OBSERVE H1, 300.0996117 MHz
DATA PROCESSING
Resol. enhancement -0.0 Hz
FT size 32768
Total time 2 min, 14 sec
OH
MOMO
HO
O
OMOM
MOMO
47
8
7
6
5
4
3
2
1
ppm
0.86 0.85 1.30 1.15 0.85 0.93 2.34 0.78 1.60 2.79 2.00 5.15
0.83 1.48 0.43 5.39 1.76 2.72 2.76 2.55 6.14 2.13 0.86

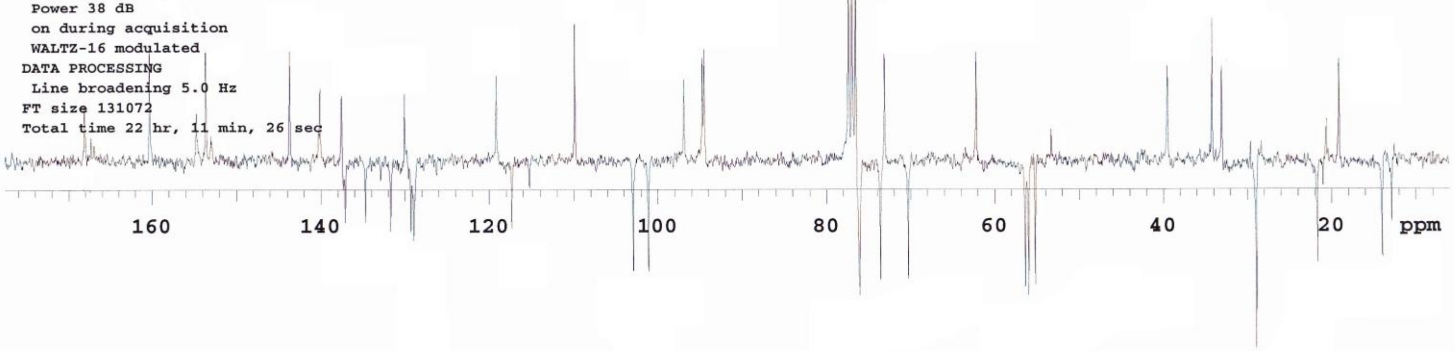
13C OBSERVE
Pulse Sequence: apt
Solvent: cdcl3
Temp. 25.0 C / 298.1 K
Mercury-300BB "m300"
Date: Jul 21 2010
Relax. delay 5.000 sec
1st pulse 180.0 degrees
2nd pulse 45.0 degrees
Acq. time 1.815 sec
Width 20000.0 Hz
401 repetitions
OBSERVE C13, 75.4601121 MHz
DECOUPLE H1, 300.1011633 MHz
Power 38 dB
on during acquisition
WALTZ-16 modulated
DATA PROCESSING
Line broadening 5.0 Hz
FT size 131072
Total time 22 hr, 11 min, 26 sec
160
140
120
100
80
60
40
20
ppm

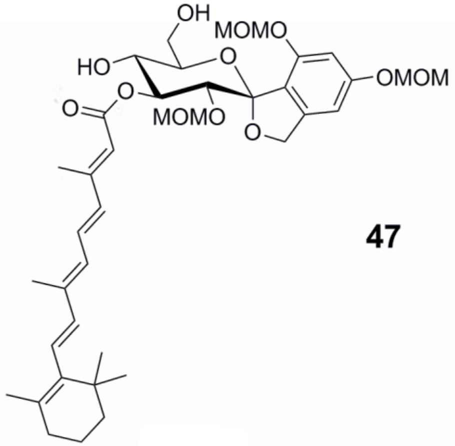
OH
MOMO
HO
O
OMOM
O
MOMO
O

47

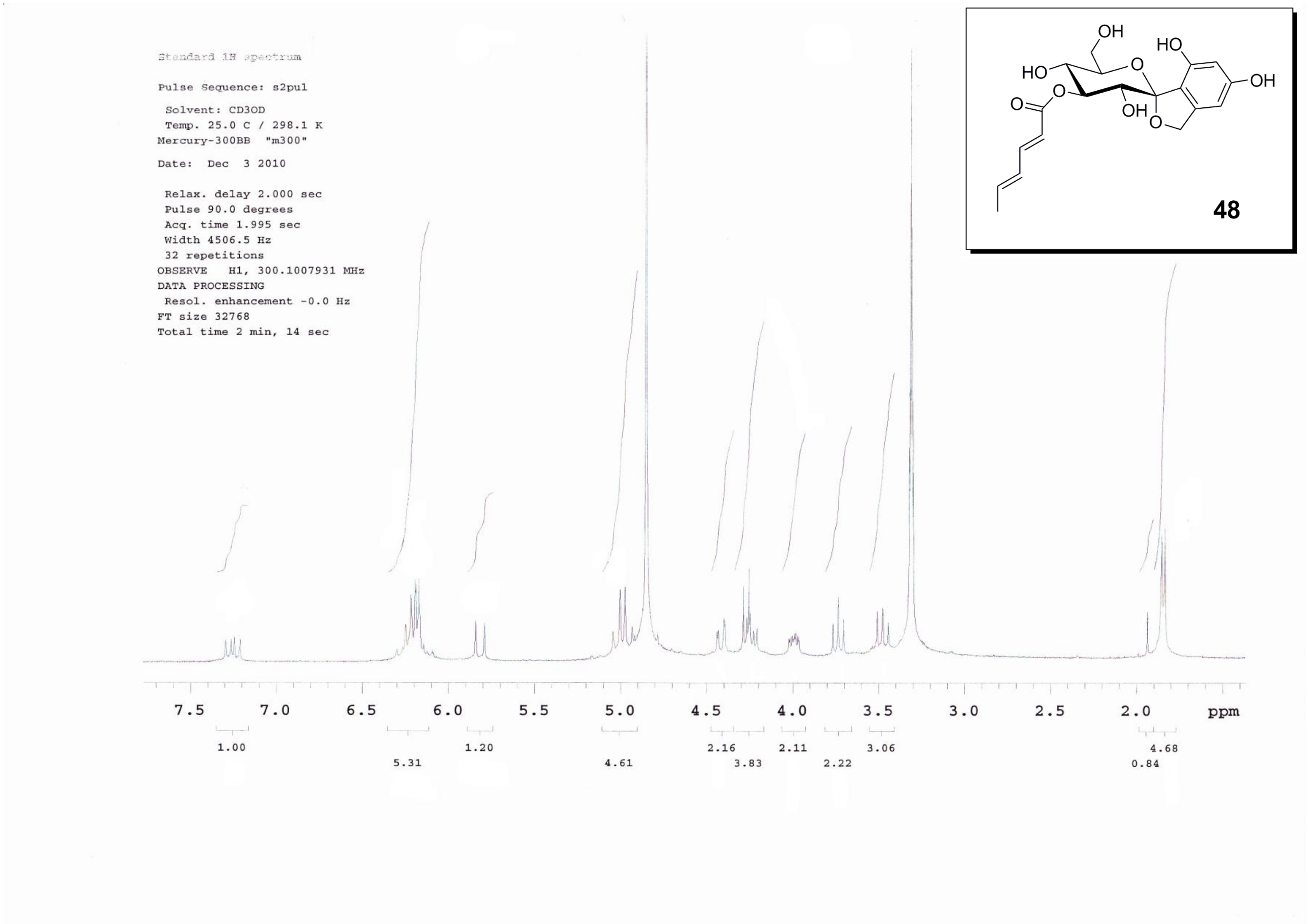
Standard 1H spectrum
Pulse Sequence: s2pul
Solvent: CD3OD
Temp. 25.0 C / 298.1 K
Mercury-300BB "m300"
Date: Dec 3 2010
Relax. delay 2.000 sec
Pulse 90.0 degrees
Acq. time 1.995 sec
Width 4506.5 Hz
32 repetitions
OBSERVE H1, 300.1007931 MHz
DATA PROCESSING
Resol. enhancement -0.0 Hz
FT size 32768
Total time 2 min, 14 sec
OH
HO
HO
O
OH
O
O
OH
O
48
7.5
7.0
6.5
6.0
5.5
5.0
4.5
4.0
3.5
3.0
2.5
2.0
ppm
1.00
5.31
1.20
4.61
2.16
3.83
2.11
2.22
3.06
4.68
0.84

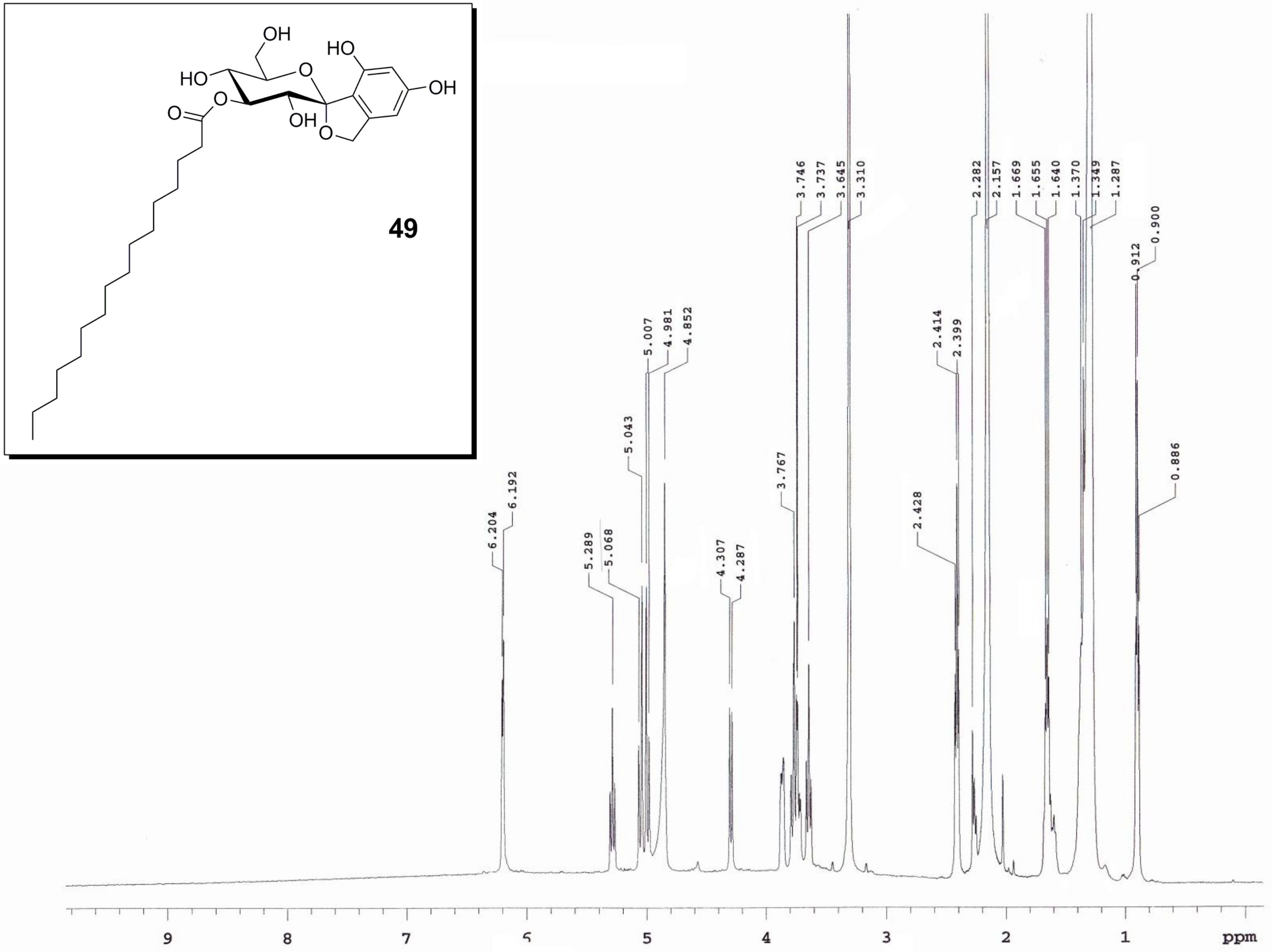
OH
HO
HO
OH
O
O
O
OH
O
6.204
6.192
5.289
5.068
5.043
5.007
4.981
4.852
4.307
4.287
3.767
3.746
3.737
3.645
3.310
2.428
2.414
2.399
2.282
2.157
1.669
1.655
1.640
1.370
1.349
1.287
0.912
0.900
0.886
9
8
7
5
4
3
2
1
ppm

49

```
13C OBSERVE

Pulse Sequence: s2pul

 Solvent: CD3OD
 Temp. 25.0 C / 298.1 K
File: MvK-C13-19102010
Mercury-300BB  "m300"

Date:  Oct 15 2010

 Relax. delay 5.000 sec
 Pulse 45.0 degrees
 Acq. time 1.815 sec
 Width 20000.0 Hz
 10000000 repetitions
OBSERVE  C13,  75.4602982 MHz
DECOUPLE  H1, 300.1023457 MHz
 Power 38 dB
 continuously on
 WALTZ-16 modulated
DATA PROCESSING
 Line broadening 5.0 Hz
FT size 131072
Total time 22141 hr, 14 min, 16 sec
```

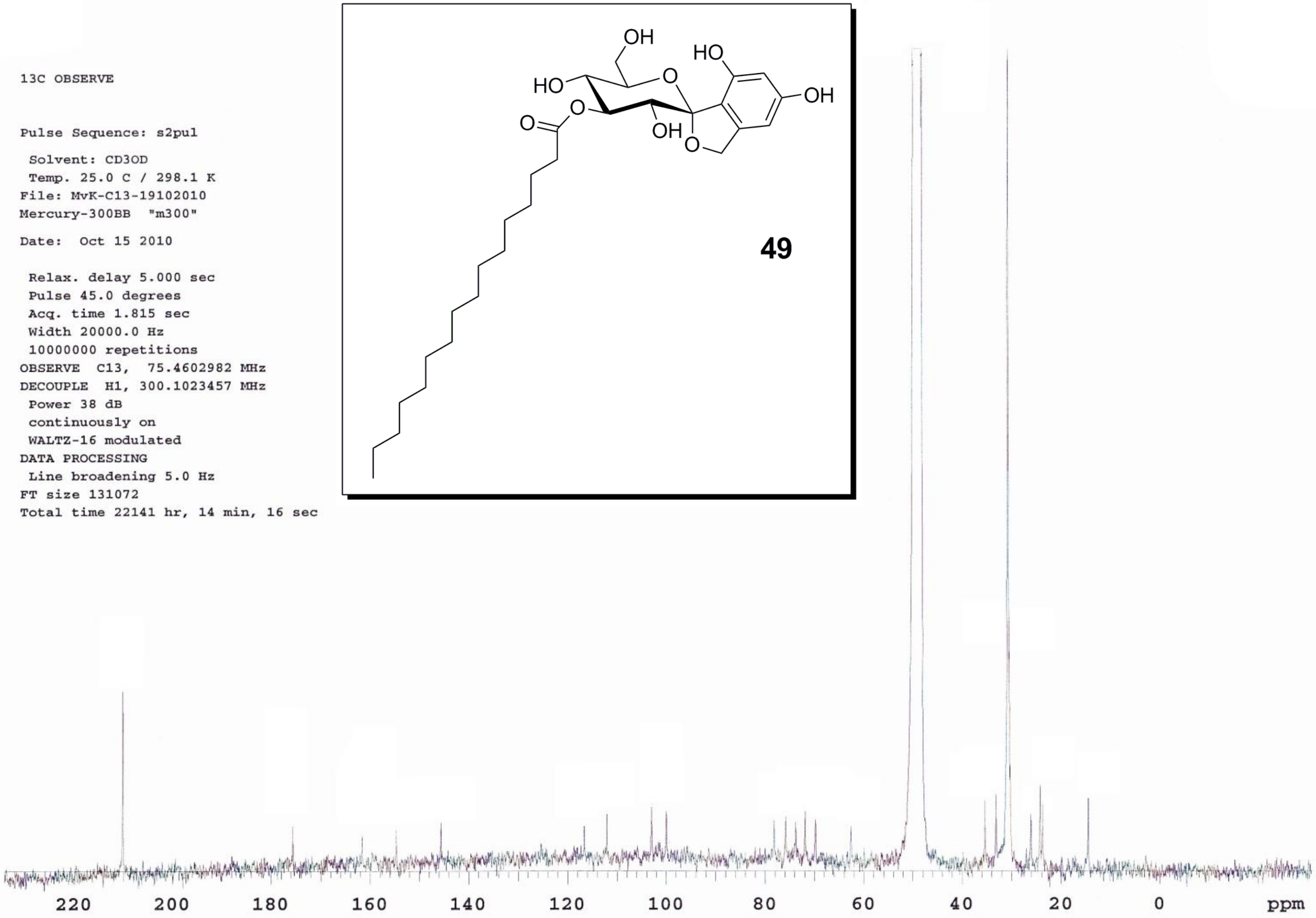

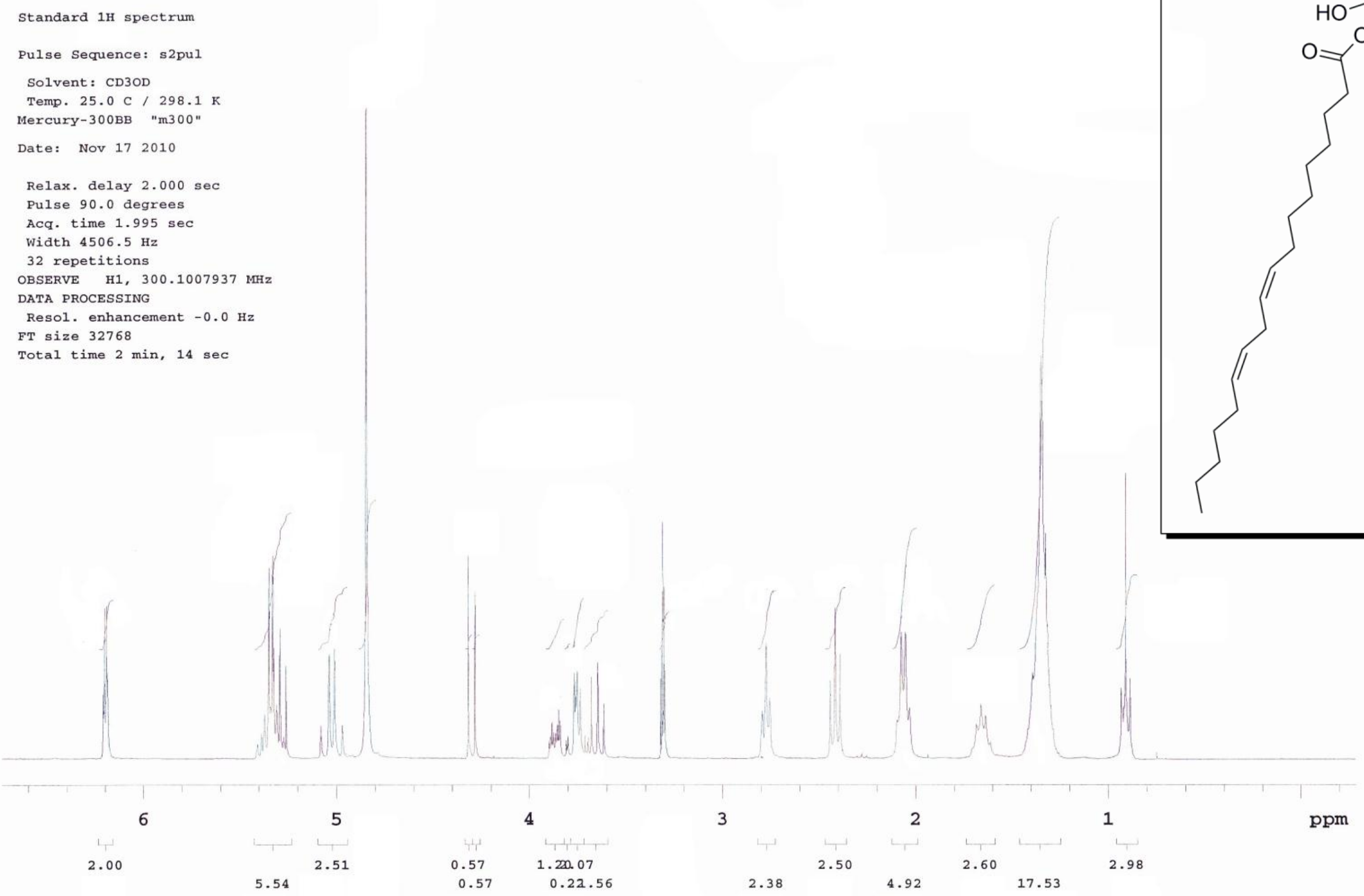
Standard 1H spectrum
Pulse Sequence: s2pul
Solvent: CD3OD
Temp. 25.0 C / 298.1 K
Mercury-300BB "m300"
Date: Nov 17 2010
Relax. delay 2.000 sec
Pulse 90.0 degrees
Acq. time 1.995 sec
Width 4506.5 Hz
32 repetitions
OBSERVE H1, 300.1007937 MHz
DATA PROCESSING
Resol. enhancement -0.0 Hz
FT size 32768
Total time 2 min, 14 sec
6
5
4
3
2
1
ppm
2.00
5.54
2.51
0.57
0.57
1.20.07
0.221.56
2.38
2.50
4.92
2.60
17.53
2.98

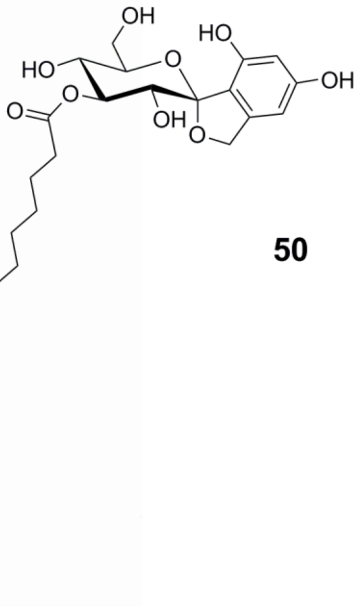
OH
HO
HO
O
OH
O
O
OH
O

50

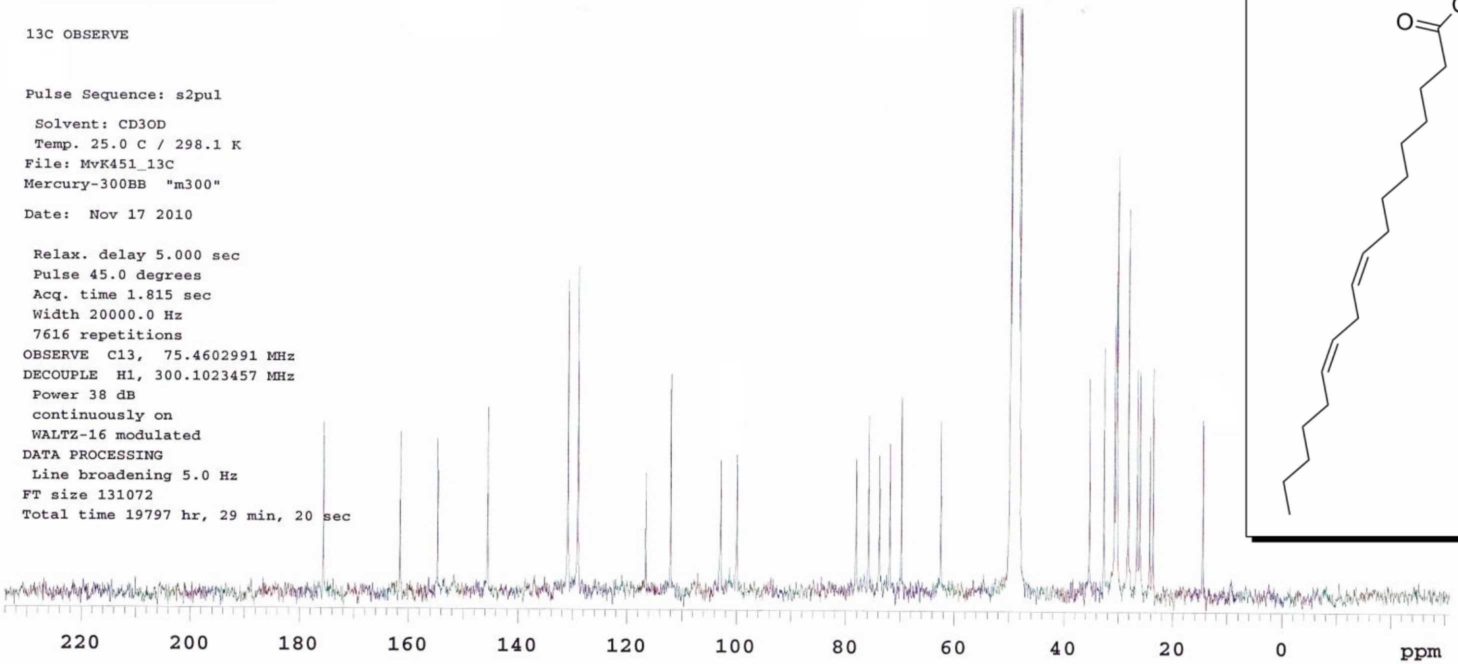
13C OBSERVE
Pulse Sequence: s2pul
Solvent: CD3OD
Temp. 25.0 C / 298.1 K
File: MvK451_13C
Mercury-300BB "m300"
Date: Nov 17 2010
Relax. delay 5.000 sec
Pulse 45.0 degrees
Acq. time 1.815 sec
Width 20000.0 Hz
7616 repetitions
OBSERVE C13, 75.4602991 MHz
DECOUPLE H1, 300.1023457 MHz
Power 38 dB
continuously on
WALTZ-16 modulated
DATA PROCESSING
Line broadening 5.0 Hz
FT size 131072
Total time 19797 hr, 29 min, 20 sec
220 200 180 160 140 120 100 80 60 40 20 0 ppm

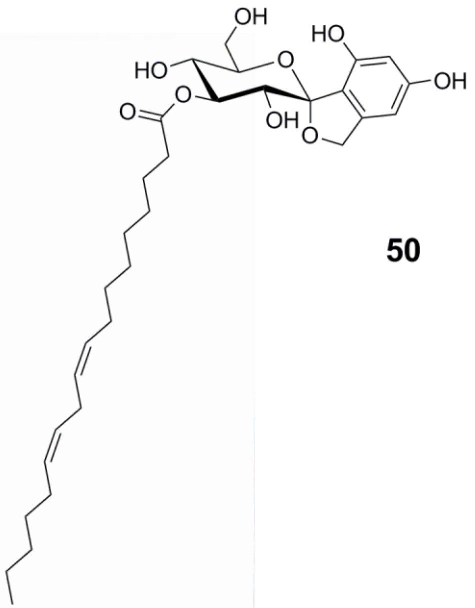
OH
HO
HO
OH
O
O
O
OH
O
O

50

Standard 1H spectrum

Pulse Sequence: s2pul

Solvent: CDCl3
Temp. 25.0 C / 298.1 K
Mercury-300BB "m300"

Date: Aug 20 2008

Relax. delay 2.000 sec
Pulse 90.0 degrees
Acq. time 1.995 sec
Width 4506.5 Hz
32 repetitions
OBSERVE H1, 300.0996130 MHz
DATA PROCESSING
Resol. enhancement -0.0 Hz
FT size 32768
Total time 2 min, 14 sec

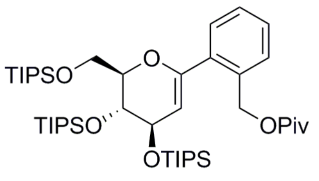

55

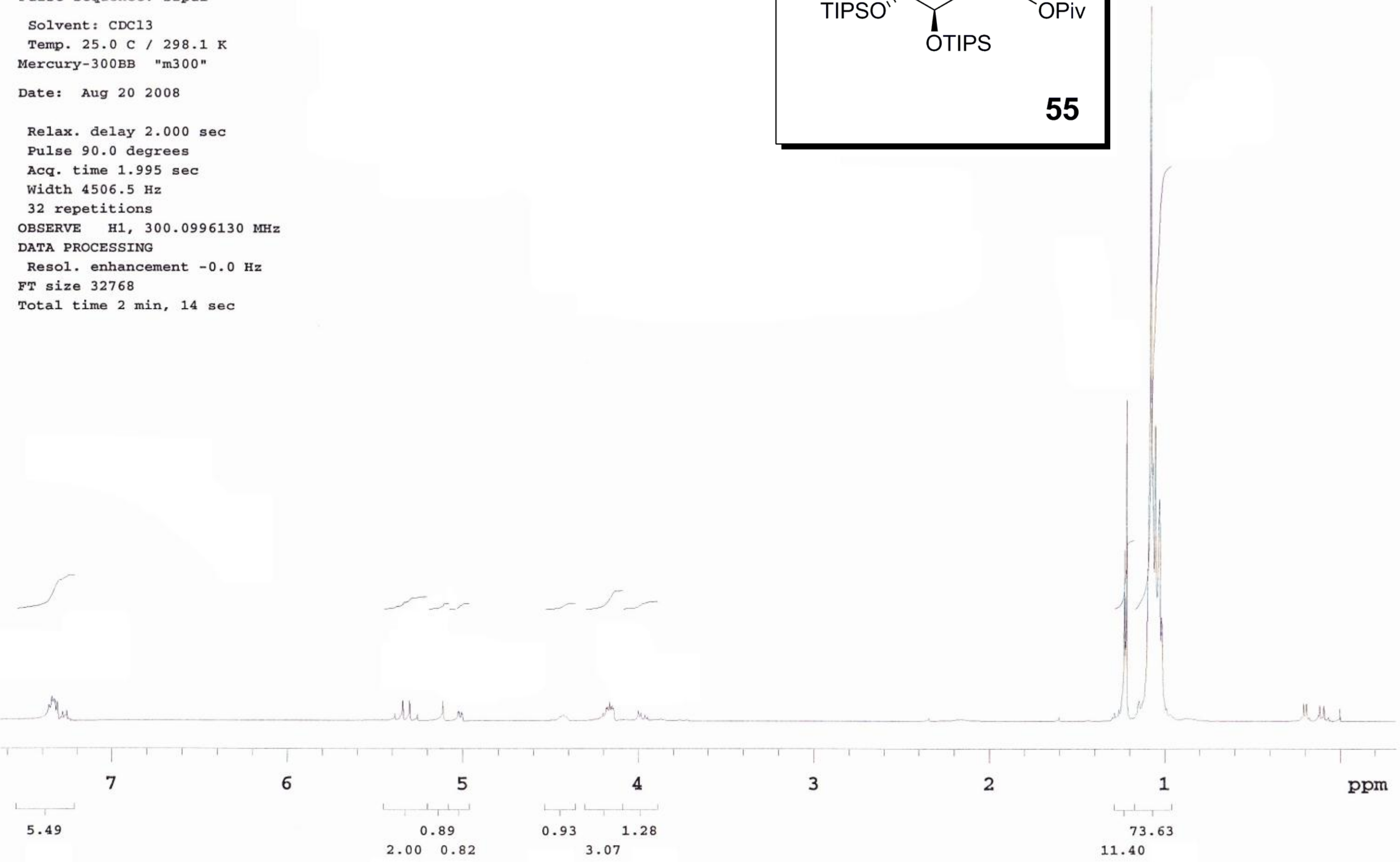

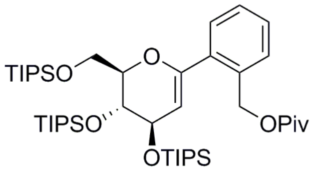

55

13C OBSERVE

Pulse Sequence: apt

Solvent: cdcl3
Temp. 25.0 C / 298.1 K
Mercury-300BB "m300"

Date: Aug 20 2008

Relax. delay 5.000 sec
1st pulse 180.0 degrees
2nd pulse 45.0 degrees
Acq. time 1.815 sec
Width 20000.0 Hz
411 repetitions
OBSERVE C13, 75.4601091 MHz
DECOUPLE H1, 300.1011633 MHz
Power 38 dB
on during acquisition
WALTZ-16 modulated
DATA PROCESSING
Line broadening 5.0 Hz
FT size 131072
Total time 221 hr, 54 min, 22 sec

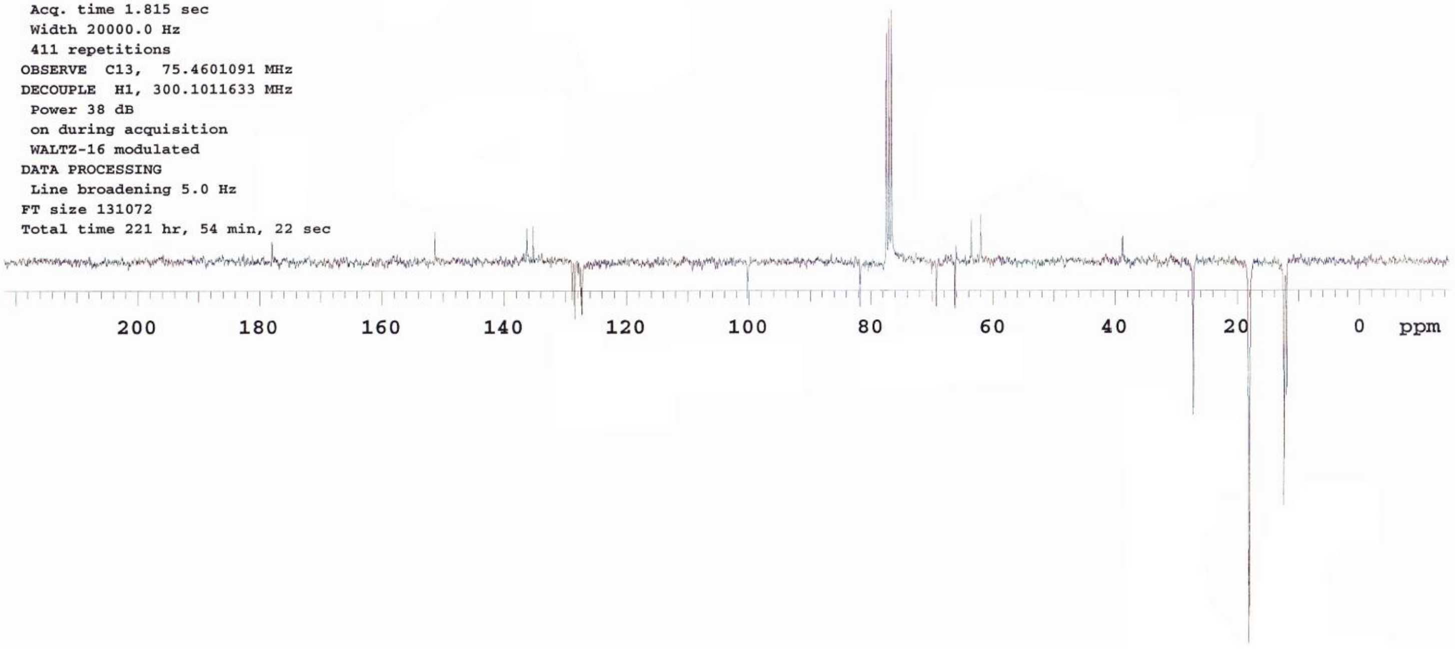

Supplement: File 1 — Synthetic procedures, the biological assay procedure and spectral data. [file Beilstein_J_Org_Chem-08-732-s001.pdf]
